# Pan American League of Associations for Rheumatology treatment recommendations for systemic juvenile idiopathic arthritis

**DOI:** 10.1093/rap/rkaf087

**Published:** 2025-11-11

**Authors:** Lorena Franco, Adriana Rodrigues Fonseca, María L Barzola, Rubén J Cuttica, Raúl Gutierrez-Suárez, Graciela Espada, Clovis Artur Silva, Simone Appenzeller, Juan A Cameto Caffa, Zoilo Morel, Ruth Eraso, Manuel A Ferrándiz, Pilar Guarnizo Zuccardi, Amparo Ibáñez Estrella, Carmen Tineo Rodríguez, Karen Viviana Jiménez Cruz, Rosario M Jurado, Beatriz H León, Cristina N Herrera, Ivonne L Arroyo Rivera, Enrique Faugier Fuentes, Ariana Ringer, Luis A Ramírez Stieben, Lucas R Brun, Nicolas M Marín Zúcaro, Daniel G Fernández-Ávila, María Lorena Brance, Eduardo Talesnik, for the Pan American League of Associations for Rheumatology (PANLAR), Lorena Franco, Adriana Rodrigues Fonseca, María L Barzola, Rubén J Cuttica, Raúl Gutiérrez-Suárez, Graciela Espada, Clovis A Silva, Simone Appenzeller, Juan A Cameto Caffa, Zoilo Morel, Ruth Eraso, Manuel A Ferrándiz, Pilar Guarnizo Zuccardi, Amparo Ibañez Estrella, Carmen Rodriguez Tineo, Karen V Jiménez Cruz, Rosario M Jurado, Beatriz H León, Cristina N Herrera, Ivonne L Arroyo Rivera, Enrique Faugier Fuentes, Ariana Ringer, Luis A Ramirez Stieben, Lucas R Brun, Nicolás M Marín Zúcaro, Daniel G Fernández-Ávila, María L Brance, Eduardo Talesnik

**Affiliations:** Rheumatology, Hospital Infantil Municipal Córdoba, Hospital Privado Universitario, Córdoba, Argentina; Paediatric Rheumatology, Instituto de Puericultura e Pediatria Martagão Gesteira, Universidade Federal do Rio de Janeiro, Rio de Janeiro, Brazil; Rheumatology, Hospital de Niños Ricardo Gutiérrez, Universidad de Buenos Aires, Buenos Aires, Argentina; Rheumatology, Hospital General de Niños Pedro de Elizalde, Buenos Aires, Argentina; Paediatric Rheumatology, Hospital del Niño, Instituto Materno Infantil del Estado de México, Toluca, Mexico; Rheumatology, Hospital de Niños Dr. Ricardo Gutierrez, Buenos Aires, Argentina; Paediatrics, Hospital das Clínicas da Faculdade de Medicina da Universidade de São Paulo, São Paulo, Brazil; Rheumatology Unit, School of Medical Science, Universidade Estadual de Campinas, Campinas, Brazil; Rheumatology, Centro Hospitalario Pereira Rossell, Montevideo, Uruguay; Paediatrics, Universidad Nacional de Asunción, San Lorenzo, Paraguay; Paediatric Rheumatology, Hospital Pablo Tobón Uribe, Medellín, Colombia; Paediatric Rheumatology, Facultad de Medicina, Universidad de Antioquia, Medellín, Colombia; Rheumatology, Instituto Nacional de Salud del Niño, Lima, Perú; Paediatrics, Fundación Cardioinfantil de Bogotá, Bogotá, Colombia; Paediatrics, Instituto Nacional de Salud del Niño Breña, Lima, Peru; Rheumatology, Hospital Cabral y Báez, Santiago, Dominican Republic; Rheumatology, Hospital Infantil Arturo Grullón, Santiago, Dominican Republic; Universidad El Bosque, Clínica Pediátrica, Clínica Colsanitas, Bogotá, Colombia; Paediatrics, Centro Hospitalario Pereira Rossell, Montevideo, Uruguay; Paediatrics, Universidad San Francisco de Quito, Quito, Ecuador; Paediatric Rheumatology, Hospital Roberto Gilbert Elizalde, Guayaquil, Ecuador; Paediatrics, San Juan City Hospital, San Juan, Puerto Rico; Paediatric Rheumatology, Hospital Infantil de México Federico Gomez, Mexico City, Mexico; Rheumatology, Rosario National University, Santa Fe, Argentina; Bone Biology Laboratory, Rosario National University, Santa Fe, Argentina; Bone Biology Laboratory, Rosario National University, Santa Fe, Argentina; Rheumatology Unit, Italian Hospital, Buenos Aires, Argentina; Paediatrics, Pontificia Universidad Javeriana, Bogota, Colombia; Bone Biology Laboratory, Rosario National University, Santa Fe, Argentina; Paediatric Infectious Diseases and Immunology, School of Medicine, Pontificia Universidad Católica de Chile, Santiago, Chile

**Keywords:** JIA, systemic, treatment, recommendations, PANLAR

## Abstract

We aimed to develop evidence-based Pan American League of Associations for Rheumatology recommendations for the pharmacological treatment of systemic juvenile idiopathic arthritis (sJIA). An expert panel of paediatric rheumatologists from Latin America generated clinically meaningful research questions structured using the Population, Intervention, Comparator and Outcome (PICO) format, adhering to Grading of Recommendations Assessment, Development, and Evaluation methodology. A team of methodologists conducted a systematic literature review, extracted and summarized intervention effect estimates and graded the evidence quality. The JIA expert panel voted on each research question structured using the PICO format, requiring a minimum agreement of 70% among the voting members to formulate recommendations. Four evidence-based recommendations were developed, addressing the two most common phenotypes of sJIA: with predominantly systemic features and with predominantly active synovitis. The optimal therapeutic approach emphasizes the early initiation of IL-1 or IL-6 pathway inhibition, coupled with a short-course corticosteroid regimen. For sJIA patients with predominantly systemic features and high disease activity, high-dose i.v. methylprednisolone (‘pulse therapy’) is advised. These recommendations highlight the importance of limiting glucocorticoid therapy to the lowest effective dose for the shortest possible duration, with gradual tapering and discontinuation within a maximum period of 6 months. These recommendations provide guidance on strategies for the use of pharmacological agents for sJIA patients.

## Introduction

Systemic juvenile idiopathic arthritis (sJIA) occurs across all geographic regions and ethnicities, with a peak incidence between 1 and 5 years of age, without sex predominance [1]. In European studies, sJIA accounts for 4–9% of all JIA cases, while Asian epidemiological studies report a higher proportion of this subtype [2]. Unfortunately, there is a scarcity of data regarding the incidence or prevalence of sJIA in Latin American countries. The Epidemiology, treatment and Outcome of Childhood Arthritis (EPOCA) study, a multinational, cross-sectional and observational cohort study, found that sJIA was more prevalent in Southeast Asia, Latin America, Africa and the Middle East compared with other regions [3].

Unlike other subtypes, sJIA is not a classic autoimmune disease, characterized by extra-articular symptoms such as fever, lymphadenopathy, rash and serositis [1, 2, 4]. This subtype is considered the most severe, presenting substantial challenges in diagnosis, treatment and management. Complications such as macrophage activation syndrome (MAS) and interstitial lung disease further increase morbidity and mortality risks [4, 5]. In addition to this, sJIA can manifest in two main phenotypes, which may be explained by its pathogenesis: with predominantly systemic features, with a central role for the expression and signalling of IL-1β, as well as elevated expression of IL-18 and S100 proteins; and with predominantly active synovitis, mediated by further activation of the adaptive immune system, with participation of TNF-α [1, 4, 6, 7]. Moreover, the balance of Treg cells and Th17 cells is regarded as a determinant of joint-related outcomes and might have a role in the chronic arthritis stage of sJIA [8]. More than 50% of sJIA patients experience a persistent disease course, while 15–40% develop a monophasic course, which is generally associated with a more favourable prognosis [1, 4, 9–11].

The ILAR classification criteria are currently under review. The PRINTO has proposed new criteria that are undergoing prospective international validation [12]. According to the ILAR criteria, the presence of arthritis is required to confirm the diagnosis, however, studies indicate that ≈40% of cases may not present with arthritis at disease onset [1, 11, 13]. Since there is no specific diagnostic test, the diagnosis is made by excluding other alternative diagnoses such as malignancies, infectious diseases, other immune-mediated inflammatory diseases and monogenic autoinflammatory disorders [14].

Advances in the understanding of pathogenesis have led to significant progress in targeted therapies, such as biologic DMARDs (bDMARDs), which enhance the potential for earlier disease activity control and achievement of remission with reduced damage and disability [15]. However, in Latin American countries, access to expensive drugs, particularly bDMARDs, remains limited. Additionally, several distinct factors hinder treatment access, including delayed referral to appropriate comprehensive care, the limited number of paediatric rheumatologists and insufficient public health coverage [16–19].

The objective of these recommendations is to provide an evidence-based framework to guide healthcare providers in Latin America in managing children and adolescents with the primary phenotypes of sJIA. These are the first Pan American League of Associations for Rheumatology (PANLAR) guidelines for this condition. They emphasize the use of accessible and effective therapies within the regional context and advocate for improved access to bDMARDs and other targeted treatments to enhance the health-related quality of life of individuals with sJIA. They are not intended to replace clinical judgement or shared decision-making, nor to impose limitations on medication coverage or healthcare budgets.

These recommendations do not address the diagnosis or other key aspects of sJIA management, such as immunizations, clinical monitoring, rehabilitation and disease complications, nor treatment-related issues such as glucocorticoid-induced osteoporosis prevention/treatment. The management of complications specific to sJIA, including MAS, as well as other disease-related complications, will be addressed separately in a future recommendation.

These recommendations are intended for healthcare providers involved in managing sJIA patients, including rheumatologists, paediatricians, internists, primary care providers, specialist pharmacists and other physicians. They may also be useful for patients with sJIA.

## Methods

Full methodological details are included in Supplementary Data S1, available at *Rheumatology Advances in Practice* online.

### Stakeholder involvement

These recommendations have been made and endorsed by the PANLAR and were developed using the Grading of Recommendations Assessment, Development, and Evaluation (GRADE) [20] methodology (www.gradeworkinggroup.org) in a process led by a GRADE methodologist group and working with an expert panel of paediatric rheumatologists from Latin America. This panel defined the scope of the recommendations and generated a series of clinically meaningful research questions structured using the Population, Intervention, Comparison and Outcome (PICO) format [21] related to systemic pharmacological treatment.

### Literature search

A systematic literature search for published randomized controlled trials, non-randomized trials, cohort studies, post hoc analyses and pooled analyses was conducted in MEDLINE/PubMed (https://pubmed.ncbi.nlm.nih.gov/), the Cochrane Library (https://www.cochranelibrary.com/) and Embase (https://www.elsevier.com/products/embase/) from the beginning of each database to February 2022. Grey literature was also evaluated.

### Study selection

Rayyan software (https://rayyan-prod.qcri.org/) was used to screen literature search results. Duplicate screening of titles and abstracts was performed by two independent reviewers, with a third reviewer resolving conflicts. Eligible articles underwent full-text screening by two independent reviewers and were matched to research questions structured using the PICO format.

### Data extraction and analysis

Pooling the data for statistical analysis was done using Review Manager (RevMan) version 5.4.1 (Cochrane Collaboration, Copenhagen, Denmark). The quality of each randomized clinical trial study was assessed using the Cochrane risk of bias tool (http://handbook.cochrane.org/) while the data from observational cohort studies were analysed using the Newcastle–Ottawa Scale.

### Evidence report formulation

RevMan files were exported to GRADEpro GDT software (https://gradepro.org/) to create the GRADE profile for each research question structured using the PICO format (Appendix 5). Two independent reviewers assessed the quality of evidence for each outcome using GRADE criteria, with conflicts resolved through discussion with a third reviewer. The GRADE methodology classifies evidence into four levels (high, moderate, low, very low) based on confidence in the effect estimate [22]. Recommendations made based on expert consensus in the absence of evidence were graded as very low quality. The summary of evidence and GRADE profiles are in Supplementary Data S2 and S3, available at *Rheumatology Advances in Practice* online, respectively.

### Rigour of development

These guidelines were developed using GRADE methodology and follow the Appraisal of Guidelines, Research and Evaluation reporting checklist to ensure transparency and completeness. The sJIA expert panel made recommendations for clinical questions, requiring 70% agreement at the voting stage. Recommendations considered the risk:benefit ratio and quality of evidence for each intervention. Recommendations were classified as either strong or conditional. Strong recommendations indicated high confidence that the intervention favourably balances benefits and risks for most patients, while conditional recommendations reflected less confidence, often due to low-quality evidence.

### Disclosures and management of conflicts of interest (COIs)

Relevant COIs were those occurring within 12 months before and during the development of these recommendations. Individuals primarily employed (>51% of work time) by companies that manufacture or sell therapeutics or diagnostics were not eligible to participate.

### Data sharing

Information about these recommendations will be available on the PANLAR website (https://www.panlar.org/). Materials for patients will also be developed and made available on the PANLAR website. PANLAR plans to update these recommendations on a regular basis.

#### Outcome measures and definitions used in the treatment recommendations for sJIA

For the current recommendations, the expert panel defined sJIA patients based on clinical phenotype rather than the ILAR classification category. The sJIA was thus defined as arthritis in one or more joints with or preceded by fever of at least 2 weeks duration that is documented to be daily (‘quotidian’) for at least 3 days and accompanied by one or more of the following: evanescent rash, generalized lymphadenopathy, enlargement of the liver or spleen and serositis (pleural or pericardial) [13].

The expert panel considered two main clinical phenotypes to develop the recommendations: sJIA with predominantly systemic features and sJIA with predominantly active synovitis. These clinical phenotypes should be re-evaluated at each visit, as they may evolve or change over time.

The expert panel defined high disease activity as high fever, serositis (pericarditis), laboratory markers suggestive of imminent MAS, increased ferritin and elevated aspartate aminotransferase [23, 24].

The same panel considered that a treat-to-target approach should be implemented, adapting treatments according to disease activity, with the therapeutic target as complete response, given that multiple long-term outcome studies demonstrate that most patients (60–80%) can achieve disease remission or minimal disease activity [23, 25].

Several disease activity scores have been used in clinical trials or longitudinal observational studies, such as the ACR pediatric response and the Systemic Juvenile Arthritis Disease Activity Score (sJADAS). A specific version of the sJADAS has recently been validated [26]. This version incorporates systemic symptoms through a modified Systemic Manifestations Score in addition to the four components of the JADAS [27]. However, cut-off points for the 10-joint sJADAS (sJADAS10), which distinguish between inactive disease, minimal disease activity, moderate disease activity and high disease activity, were evaluated in only one cohort, with results published only a few months ago [26].

Although the expert panel recognizes the potential of sJADAS as an outcome measure, it decided not to adopt the sJADAS10 and the recently proposed cut-off points in these treatment recommendations. This decision was due to limited daily experience with this new outcome measure, a lack of longitudinal studies associating it with various outcomes, its absence of validation in other cohorts and a lack of association with validated Wallace criteria for remission [28].

The expert panel defined a composite outcome measure based on the JADAS10 along with simple, available and relevant clinical and laboratory criteria, taking into account as a rationale the existing recommendations proposed by the ACR [25], by a German group [11], the EULAR/Pediatric Rheumatology European Society (PReS) [23] and the outcome measures adopted in the pivotal anti-IL-1 [29–33] and anti-IL-6 trials [34, 35]. Therefore, the expert panel provides the following treatment response definitions:

- Complete response is defined as a JADAS10 score ≤1, plus fever resolution, plus a return of acute phase reactant (ESR, CRP and/or ferritin) levels to normal.
- Lack of response to treatment is defined as a JADAS10 score greater than or equal to the previously specified value for any intervention or treatment modification, along with no improvement in the fever curve and serum ferritin levels.
- Insufficient treatment response is defined as a JADAS10 score that does not significantly decrease from the previous value, based on the clinical judgement of the treating physician, despite any intervention or treatment modification. It also includes the absence of fever resolution and normalization of serum ferritin levels.
- Refractory sJIA is defined as the presence of active systemic or arthritis feature manifestations despite treatment with anti-IL-1 or anti-IL-6 therapy or the need for ongoing treatment with long-term glucocorticoids (>6 months) [4].

In these cases, it is mandatory to initiate a new treatment approach or dose modification, according to the specific algorithms proposed in these recommendations.

Additionally, the panel defined bDMARD failure as either a lack of or insufficient response to treatment, or the occurrence of adverse effects. Two types of bDMARD failure are distinguished based on the timing of onset: primary failure, which occurs within 3 months of initiating bDMARD therapy, and secondary failure, which develops after an initial period of effectiveness (>3 months) or due to the development of side effects over time. While measuring anti-drug antibodies is recommended to assess the safety and effectiveness of biologic therapies, this practice is not routinely performed in most Latin American centres.

## Recommendations

**Figure 1.**
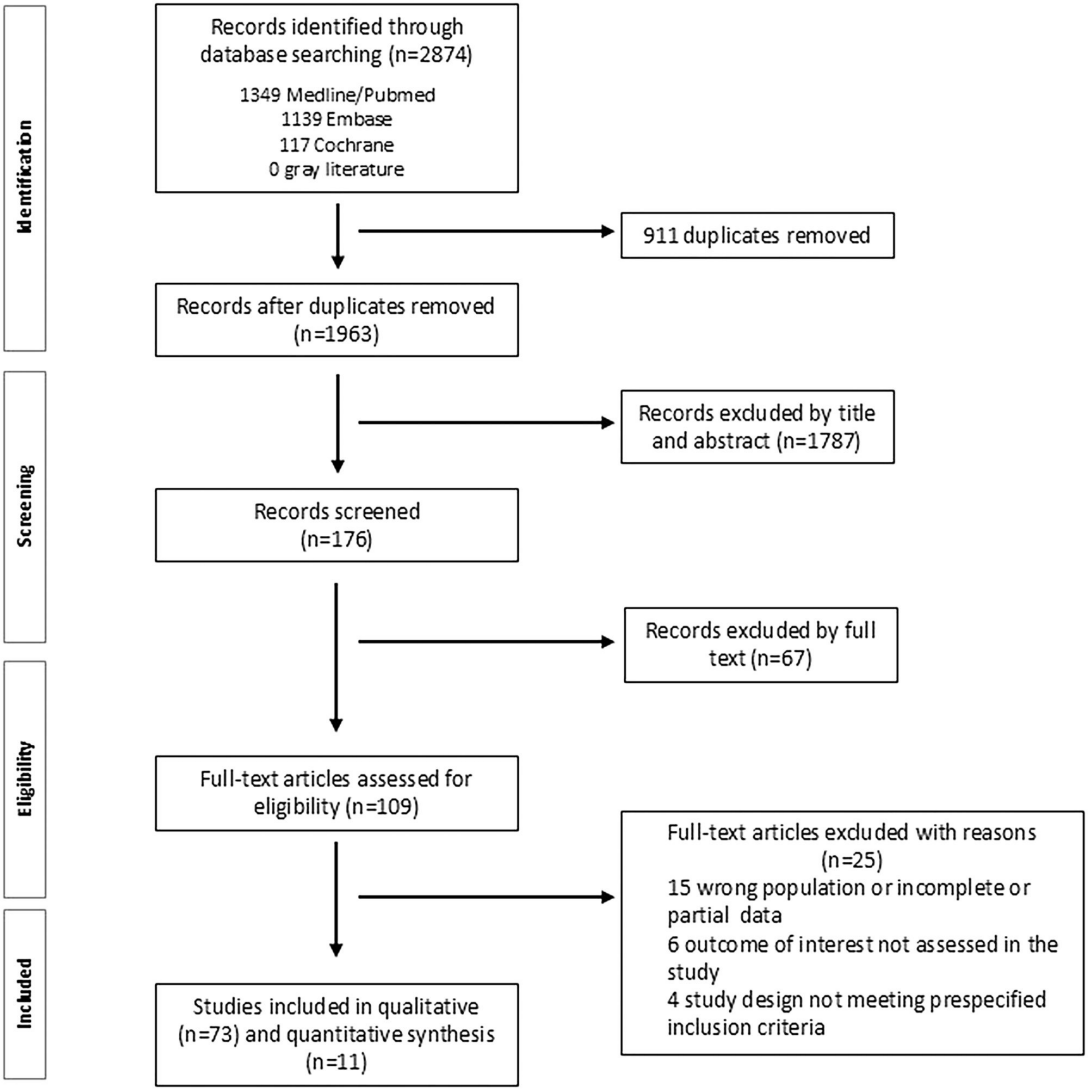
Literature search ﬂow chart

**Figure 2.**
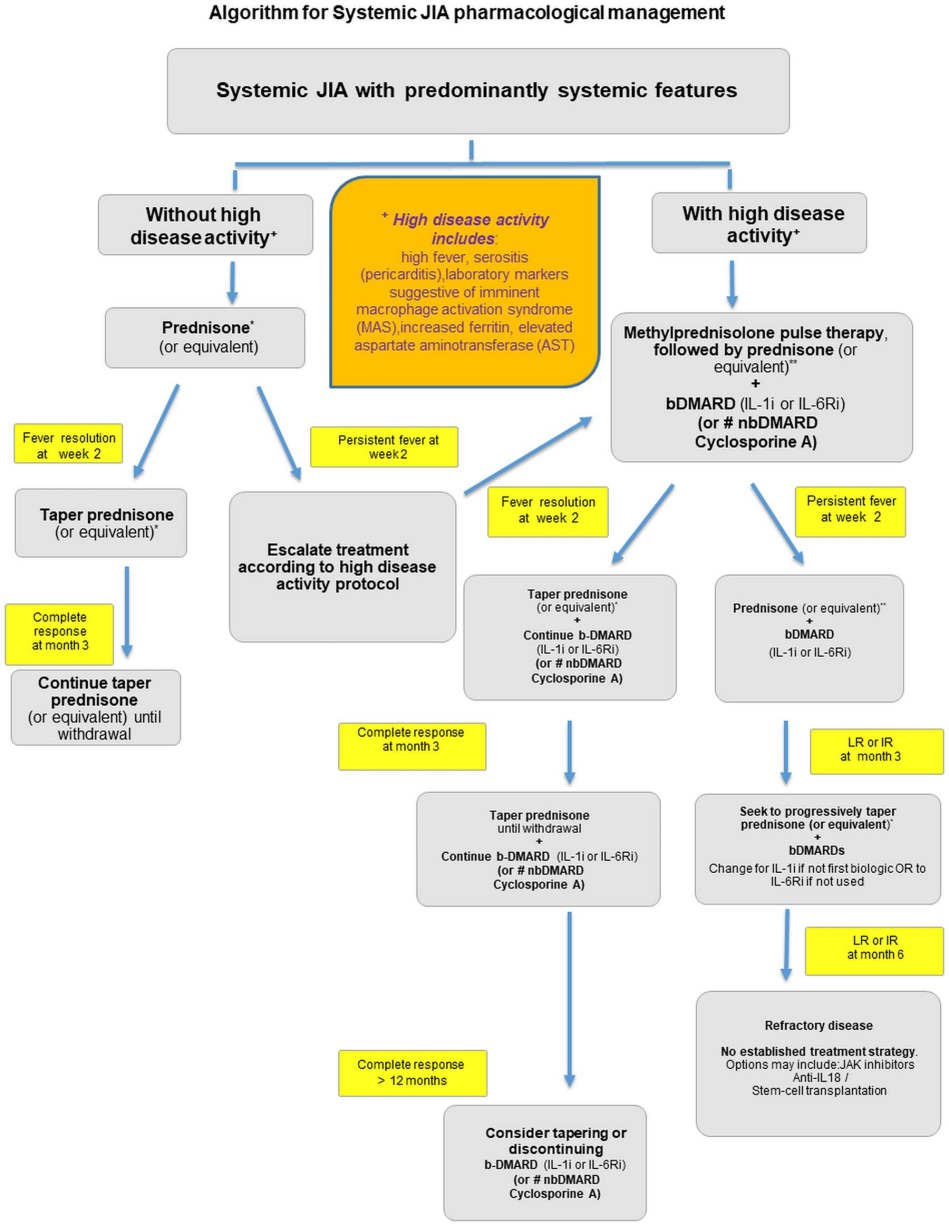
Algorithm proposed for the pharmacological management of sJIA with predominantly systemic features. Patients are classified according to the presence or absence of high disease activity, which should be reassessed at each visit based on treatment response and disease severity. If LR (Low Response) or IR (Insufficient Response) is observed, treatment should be adjusted. *Prednisone: 0.5–1 mg/kg/day (maximum 60 mg/day). **High-dose i.v. methylprednisolone: 10–30 mg/kg/dose (maximum 1 g/day) for 3–5 consecutive days. ^#^nbDMARD: non-biologic DMARDS (cyclosporine A). bDMARDs: IL-1i: IL-1 inhibitors, which inhibit IL-1 signalling via cytokine neutralization or receptor blockade; IL-6Ri: IL-6 receptor inhibitor. Refractory disease: consider targeting IL-18, IFN-γ, JAK pathways or using a bispecific antibody to IL-1β/IL-18. Stem cell transplantation may also be considered

**Figure 3.**
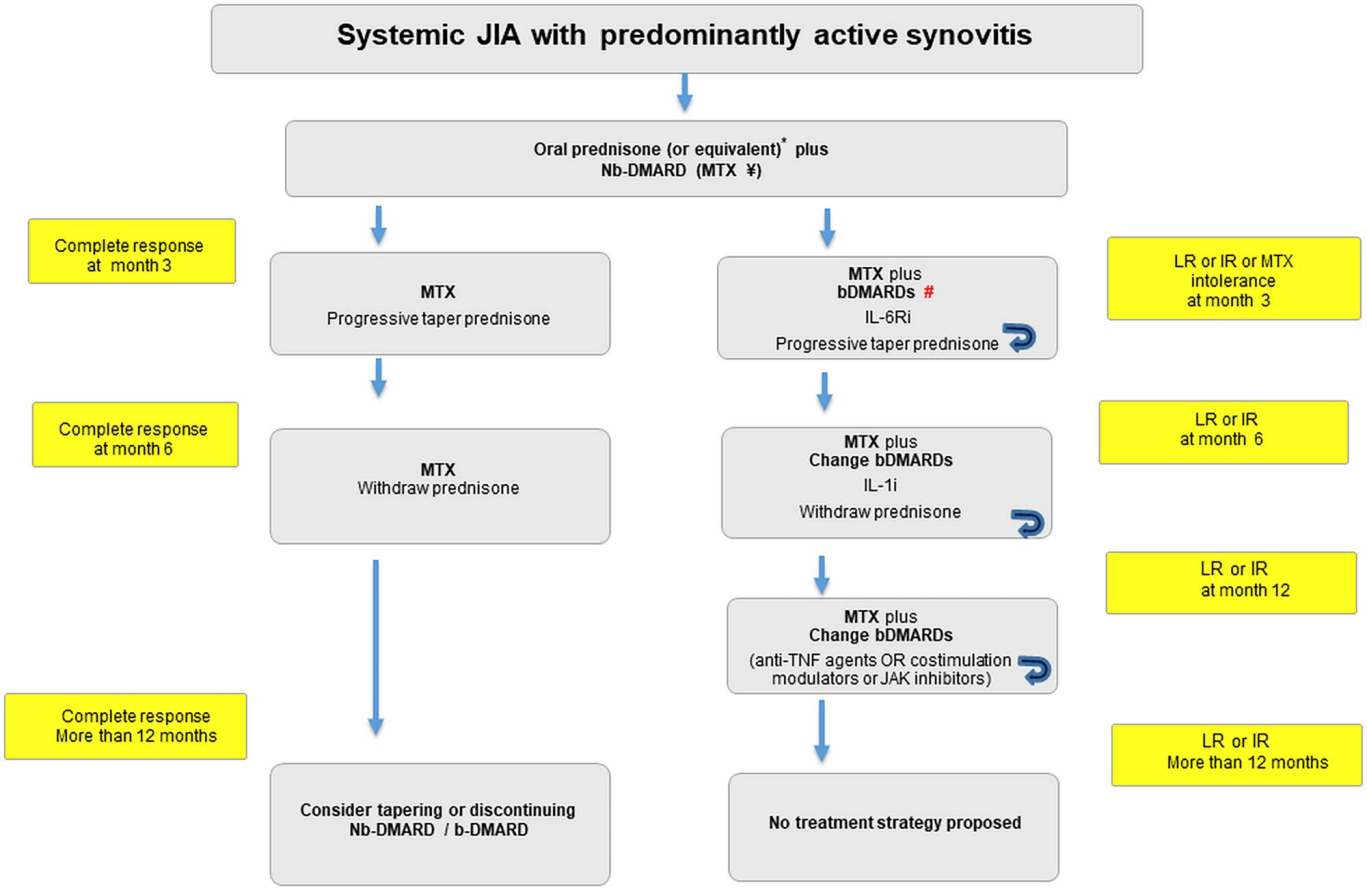
Algorithm proposed for pharmacological management of sJIA in patients with predominantly active synovitis. *Prednisone (or equivalent): 0.5–1 mg/kg/day (maximum 60 mg/day) for the lowest dose and duration possible. Associate a non-biologic DMARD: MTX oral dose: 10–20 mg/m^2^/week (maximum 15 mg). If a higher dose is required, switch to intramuscular (i.m.) or subcutaneous (s.c.) route (maximum 25 mg). Treatment adjustment: if low response (LR) or insufficient response (IR) is observed after 2–3 months of non-biologic DMARD therapy, initiate a bDMARD. bDMARDs: IL-6Ri: IL-6 receptor inhibitor. Preferred option among bDMARDs for sJIA with predominantly active synovitis. IL-1i: IL-1 inhibitor, which inhibit IL-1 signalling via cytokine neutralization or receptor blockade. If LR/IR persists, switch to anti-TNF-α (infliximab, etanercept, adalimumab) or co-stimulation modulators (abatacept). In the event of clinical improvement, the patient may transition to the treatment strategy on the left-hand side, including the gradual tapering and possible discontinuation of glucocorticoids and conventional DMARDs, as appropriate

A total of nine research questions structured using the PICO format were generated (Supplementary Data S1, available at *Rheumatology Advances in Practice* online). The initial literature search identified 2874 manuscripts. Of these, 84 were selected for inclusion in the evidence report (Fig. 1). Four recommendations were developed and are detailed in Table 1. Figs 2 and 3 summarize the main treatment recommendations for patients with sJIA. The references for each recommendation are detailed in Supplementary Data S4, available at *Rheumatology Advances in Practice* online. Drugs and doses are described in Supplementary Data S5, available at *Rheumatology Advances in Practice* online.

**Table 1.** PANLAR treatment recommendations for sJIA.

Recommendation	Recommendation strength	Level of evidence	Level of agreement (%)
1. For patients with sJIA who present predominantly with systemic features and have high disease activity, initiate high-dose i.v. methylprednisolone (pulse therapy) rather than oral prednisone at 1 mg/kg/day.	Strong	Low to moderate	(84)
2. For patients with newly diagnosed sJIA presenting predominantly with systemic features and high disease activity, initiate treatment with a bDMARD targeting the IL-1 pathway (anakinra, canakinumab or rilonacept) or the IL-6 receptor (tocilizumab) as first-line therapy.	Strong	Very low to moderate	Canakinumab (100), Rilonacept (100), Anakinra (84), Tocilizumab (78)
3. For patients with newly diagnosed sJIA presenting predominantly with active synovitis, initiate treatment with MTX.	Strong	Low to moderate	(100)
4. For patients with sJIA characterized by predominantly active synovitis and an inadequate response to non-biologic DMARDs, initiate treatment with a bDMARD targeting the IL-6 receptor, such as tocilizumab.	Strong	Very low to low	(78)

### Treatment recommendations for sJIA

Given that sJIA is a severe illness, associated with high morbidity and mortality, and patients may experience adverse events, drug allergies and complications such as interstitial lung disease and MAS, it is recommended to consult a paediatric rheumatologist for management.

#### Recommendation 1

For patients with sJIA who present predominantly with systemic features and have high disease activity, initiate high-dose i.v. methylprednisolone (pulse therapy) rather than oral prednisone at 1 mg/kg/day. High-dose i.v. methylprednisolone (‘pulse therapy’) may be needed for very sick patients (high persistent fever, severe anaemia, myocarditis, pericarditis or MAS), due to its rapid onset of action. The recommended dose is 10–30 mg/kg/dose (maximum dose 1 g) for 3–5 consecutive days.

In sJIA patients with predominantly systemic features but without high disease activity, treatment with oral prednisone or equivalent, in a dose of 0.5–1 mg/kg/day (maximum dose 60 mg/day) may be considered, with tapering as soon as partial response is achieved and further withdrawal, based on a case-by-case evaluation, as shown in Fig. 2 (algorithm for sJIA pharmacological treatment for patients with predominantly systemic features).

#### Recommendation 2

For patients with newly diagnosed sJIA presenting predominantly with systemic features and high disease activity, initiate treatment with a bDMARD targeting the IL-1 pathway (anakinra, canakinumab or rilonacept) or the IL-6 receptor (tocilizumab) as first-line therapy.

Glucocorticoid therapy should be limited to the lowest effective dose for the shortest duration. To achieve a complete response and reduce steroid-related damage, adding a bDMARD is recommended.

Evidence shows that targeting the IL-1 pathway in new-onset sJIA with predominant systemic features leads to better outcomes, enabling lower glucocorticoid doses, shorter treatment durations and improved long-term outcomes. Initiating IL-1-targeted therapy as first-line treatment has shown particular effectiveness in untreated, new-onset cases. Both anakinra and canakinumab have demonstrated favourable outcomes with low systemic adverse effects, although cases of MAS have been reported with canakinumab. Few trials have compared rilonacept with placebo. It is worth mentioning that rilonacept is unavailable in most Latin American countries. Moreover, in Latin America, access to IL-1-targeted therapies is often limited due to cost and regulatory issues.

Similarly, studies on tocilizumab report significant improvements in sJIA patients, including reduced disease activity, fever resolution and long-term efficacy. Many patients discontinued glucocorticoids with sustained improvement, especially when treatment began early in the disease course. Subcutaneous tocilizumab offers similar efficacy to i.v. administration, with added convenience for patients and caregivers, particularly for home use.

However, when bDMARDs are unavailable, the use of a non-biologic DMARD may be considered. Although the expert panel did not develop a specific research questions structured using the PICO format for this drug, ciclosporin A may be an option in this scenario; however, its slower onset of action should be considered. It has demonstrated favourable outcomes, especially fever control and a significant steroid-sparing effect.

In contrast, no preferred agent has been defined between IL-1- and IL-6-targeted therapies, due to the lack of direct comparisons. The choice depends on availability, physician experience and patient/caregiver preference. Switching between these therapies is appropriate in cases of inefficacy or poor tolerability.

#### Recommendation 3

For patients with newly diagnosed sJIA presenting predominantly with active synovitis, initiate treatment with MTX.

Current and previous studies highlight a potential ‘window of opportunity’ for patients with sJIA. Early intervention with cytokine antagonists has proven effective in preventing the expansion of arthritis-inducing T cell populations. However, in settings where bDMARDs are not readily available due to cost or regulatory constraints, MTX remains a viable alternative as a first-line non-biologic DMARD. Although MTX may offer some degree of disease control, its efficacy in sJIA is limited due to the unique pathophysiological characteristics of this subtype compared with other forms of JIA.

Low-dose oral MTX *vs* placebo was studied by Woo *et al*. [36], who evaluated children with sJIA and extended oligoarthritis, with persistent arthritis for >1 year, many of whom had signs of active systemic disease. When combining data from both subgroups, a significant improvement in disease activity was observed based on assessments by physicians, parents and patients. There were notable improvements in CRP, ESR, haemoglobin, platelet count, white blood cell count, total protein and IgG levels. However, due to the characteristics of sJIA, this trial did not show a significant difference between MTX and placebo in joint scores or systemic features, nor did it lead to a reduction in steroid doses, suggesting that its utility in sJIA may be limited.

Based on the available evidence, for patients with predominantly active synovitis, if bDMARDs are not available, a combination of MTX (10–20 mg/m^2^, up to a maximum dose of 25 mg/week) and oral prednisone or its equivalent at an initial dose of 0.5–1 mg/kg/day (maximum 60 mg/day) is recommended. Ideally, until the third month the steroid dose should be tapered off gradually to a dose of 0.1–0.2 mg/kg/day and discontinued by the sixth month, as shown in Fig. 3 (algorithm for sJIA pharmacological treatment for patients with predominantly active synovitis).

#### Recommendation 4

For patients with sJIA characterized by predominantly active synovitis and an inadequate response to non-biologic DMARDs, initiate treatment with a bDMARD targeting the IL-6 receptor, such as tocilizumab. Targeting IL-6 receptors with tocilizumab is an effective therapeutic option for the treatment of sJIA with polyarticular involvement. Previous studies have demonstrated the effectiveness of tocilizumab in improving joint involvement and radiographic outcomes in children with sJIA, particularly those with a polyarticular disease course.

Medical literature also suggests that tocilizumab may improve radiographic joint damage in children with sJIA. An observational study demonstrated radiographic improvement in large, damaged joints of children treated with tocilizumab.

Additionally, a post hoc analysis of two randomized controlled trials indicated that tocilizumab led to significant improvements in growth and joint remodelling. It may also offer benefits in reducing radiographic progression in sJIA, as well as in JIA with a polyarticular course.

Moreover, the phase 3 study conducted by De Benedetti *et al.* [34] provided significant evidence that treatment with tocilizumab in patients with sJIA can lead to notable catch-up growth and improvements in disease activity. During treatment, patients experienced growth velocities exceeding normal rates, suggesting a substantial recovery in growth.

As experts, we acknowledge the lack of comparative studies supporting the use of IL-6 receptor–targeted therapy as a first-line treatment. Nevertheless, the decision to recommend targeting the IL-6 receptor as part of the treatment for sJIA, particularly in patients with polyarticular involvement, is primarily based on expert opinion. This recommendation is driven by promising observational data and the general consensus on the potential benefits of IL-6 receptor–targeted therapy in this population.

Additionally, new agents are being developed, studied and even used for compassionate purposes, including biologic products targeting IL-18, IFN-γ or both IL-1β and IL-18 simultaneously, as well as small molecules such as Janus kinase (JAK) inhibitors and stem cell transplantation. However, at the time of writing of these recommendations, there were no controlled studies or systematic reviews on these drugs.

Fig. 2 shows the algorithm proposed for JIA with predominantly systemic features and Fig. 3 shows the algorithm for sJIA with predominantly active synovitis.

### Special consideration from experts for MAS

It is essential to consider MAS as a potential complication in patients with sJIA. MAS should be suspected in the presence of persistent, unexplained fever, hepatosplenomegaly, cytopenia, liver dysfunction and neurological symptoms. If undiagnosed or untreated, MAS can lead to haemodynamic instability, multi-organ failure and death.

Distinguishing MAS from disease flares, infection or sepsis can be challenging. Additionally, assessing its association with interstitial pulmonary disease and allergy to biologics should also be considered.

Sequential laboratory assessments are more valuable than single time-point values. CRP is universally elevated, however, despite systemic inflammation, ESR paradoxically decreases due to consumptive coagulopathy. Elevated ferritin is a key biomarker, while other abnormalities include hypertriglyceridaemia, high D-dimer, elevated transaminases and lactate dehydrogenase and low or declining fibrinogen. Bone marrow findings often reveal haemophagocytic macrophages, although their presence is not always required, and this study is not routinely performed [24, 37].

Given the severity of MAS, early recognition and a multidisciplinary approach are essential for optimal diagnosis and management.

In 2016, the Classification Criteria for MAS Complicating sJIA, developed through a collaborative initiative by the EULAR, ACR and PRINTO, established diagnostic criteria to support clinicians in identifying this serious complication [24].

## Discussion

### Challenges

Undoubtedly, the treatment of sJIA is full of challenges, which are even more pronounced in Latin American countries, where the region’s specific characteristics complicate effective management and compromise long-term outcomes. These challenges arise from both the nature of the disease and the economic and structural limitations of healthcare systems in the region [16–18].

sJIA is one of the most severe and complex forms of JIA due to its unique pathogenesis, which requires early and aggressive intervention to prevent potentially life-threatening complications such as MAS [1, 2, 4, 5, 10, 11]. Despite notable advances in the treatment of sJIA over the past 2 decades, particularly with the introduction of biologic therapies targeting key cytokines, such as IL-1 and IL-6 inhibitors, access to these medications, especially IL-1 inhibitors, remains limited in many Latin American countries due to their high costs, which vary across the region [4, 19].

In addition to the cost of medications, families of patients often encounter significant challenges, including transportation difficulties to specialized centres, loss of income due to the need for continuous care and indirect expenses associated with long-term treatment. These economic burdens can lead to interruptions in treatment, with the consequent risk of disease exacerbations and severe complications.

Managing sJIA requires specialized care due to its complexity and the risks associated with treatment. However, in many areas of Latin America there is a limited number of paediatric rheumatologists, which means that patients are often treated by physicians without the specific training required, leading to misdiagnoses, inadequate or delayed treatments and worse outcomes for patients [16–19]. Although other international guidelines for managing sJIA have been published previously, such as those from the ACR, EULAR/PReS and the German group, among others [11, 23, 25], these are often based on different realities, hindering their feasibility in the Latin American context. The main differences in these proposed recommendations lie in their adaptation to the challenges encountered in real-life practice scenarios in underdeveloped Latin American countries.

### NSAIDs

Although the panel did not develop a specific PICO question for NSAID use, some studies propose a short trial of NSAIDs, as a small proportion of sJIA patients may respond favourably. However, since steroids are recommended for these patients, no specific recommendation for NSAIDs was made due to the risk of adverse events associated with the combined use of these drugs [38].

### Comparisons with existing guidelines

The PANLAR sJIA recommendations differ in several key respects from the 2021 ACR guidelines [25]. First, the ACR recommendations consider sJIA patients with or without MAS at the onset of the disease. Second, the ACR guidelines strongly advise against using conventional synthetic DMARDs as initial monotherapy for sJIA. In contrast, the PANLAR recommendations categorize sJIA into two main clinical phenotypes: sJIA with predominantly systemic features and with predominantly active synovitis. Additionally, due to limited availability, delayed access to IL-6 receptor inhibitors (in some Latin American countries) and especially to IL-1 inhibitors (in most Latin American countries), the PANLAR expert panel recommended MTX as first-line therapy for sJIA patients with predominantly active synovitis. This recommendation also takes into account MTX’s demonstrated efficacy in other categories of JIA.

For the phenotype with predominantly systemic features, ciclosporin A may be considered in situations with limited access to bDMARDs. This is due to its favourable effects on fever control and its steroid-sparing properties, although it has a longer onset of action.

The EULAR/PReS recommendations published in 2024 were not based on clinical sJIA phenotypes, but rather on the similarities with adult-onset Still disease and complications. The optimal therapeutic strategy relies on early use of interleukin (IL-1- or IL-6-targeted therapies) associated with a short course of glucocorticoids. As in the PANLAR proposed recommendations, EULAR/PReS also emphasized the key role of expert centres for difficult-to-treat patients [23].

## Conclusions

The first PANLAR JIA treatment recommendations provide evidence-based recommendations for healthcare providers treating patients with sJIA in Latin America.

## Supplementary Material

rkaf087_Supplementary_Data

## Data Availability

The data underlying this article are available in the article and in its online supplementary material.
