# Supplementary material for: Pan American League of Associations for Rheumatology treatment recommendations for systemic juvenile idiopathic arthritis
Source: Rheumatol Adv Pract. 2025 Nov 11;9(4):rkaf087. doi: 10.1093/rap/rkaf087 (PMC12607261; doi:10.1093/rap/rkaf087)
Supplement: rkaf087_Supplementary_Data [file rkaf087_supplementary_data.zip › Supplementary Data 3. GRADE Profile Systemic JIA.pdf]

Juvenile idiopathic arthritis (JIA): Systemic

GRADE Profile

Methylprednisolone (pulse) compared to Prednisone (1 mg/kg/day) for systemic JIA

Bibliography: Picco P, Gattorno M, Buoncompagni A, Pistoia V, Borrone C. 6-methylprednisolone 'mini-pulses': a new modality of glucocorticoid treatment in systemic onset juvenile chronic arthritis. Scand J Rheumatol. 1996;25(1):24-7. doi: 10.3109/03009749609082663. PMID: 8774551.

Certainty assessment							Summary of findings				
Participants (studies) Follow-up	Risk of bias	Inconsistency	Indirectness	Imprecision	Publication bias	Overall certainty of evidence	Study event rates (%)		Relative effect (95% CI)	Anticipated absolute effects	
							With Prednisone (1 mg/kg/day)	With Methylprednisolone (pulse)		Risk with Prednisone (1 mg/kg/day)	Risk difference with Methylprednisolone (pulse)
**CRP month 6**											
40 (1 RCT)	not serious	not serious	not serious	serious[a]	none	⊕⊕⊕◯ Moderate	20	20	-	The mean CRP month 6 was **0**	MD **1.4 lower** (2.03 lower to 0.77 lower)
**ERS month 6**											
40 (1 RCT)	not serious	not serious	not serious	serious[b]	none	⊕⊕⊕◯ Moderate	20	20	-	The mean ERS month 6 was **0**	MD **9.4 higher** (4.9 lower to 23.7 higher)
**Cumulative steroid dosage**											
40 (1 RCT)	not serious	not serious	not serious	serious[c]	none	⊕⊕⊕◯ Moderate	20	20	-	The mean cumulative steroid dosage was **0**	MD **0.4 lower** (0.54 lower to 0.26 lower)

CI: confidence interval; **MD:** mean difference

Explanations

a,b,c. Only one study.

MTX compared to placebo for systemic JIA

Bibliography: Woo P, Southwood TR, Prieur AM, Doré CJ, Grainger J, David J, Ryder C, Hasson N, Hall A, Lemelle I. Randomized, placebo-controlled, crossover trial of low-dose oral methotrexate in children with extended oligoarticular or systemic arthritis. Arthritis Rheum. 2000 Aug;43(8):1849-57. doi: 10.1002/1529-0131(200008)43:8<1849::AID-ANR22>3.0.CO;2-F. PMID: 10943876.

Certainty assessment							Summary of findings				
Participants (studies) Follow-up	Risk of bias	Inconsistency	Indirectness	Imprecision	Publication bias	Overall certainty of evidence	Study event rates (%)		Relative effect (95% CI)	Anticipated absolute effects	
							With placebo	With MTX		Risk with placebo	Risk difference with MTX
**Physician's assessmentof disease activity improvement**											
70 (1 RCT)	not serious	not serious	not serious	very serious[a]	none	⊕⊕◯◯ Low	17/36 (47.2%)	12/34 (35.3%)	**RR 0.75** (0.42 to 1.32)	472 per 1000	**118 fewer per 1000** (from 274 fewer to 151 more)
**Parent's assessmentof disease activity improvement**											
66 (1 RCT)	not serious	not serious	not serious	very serious[b]	none	⊕⊕◯◯ Low	14/34 (41.2%)	12/32 (37.5%)	**RR 0.91** (0.50 to 1.66)	412 per 1000	**37 fewer per 1000** (from 206 fewer to 272 more)
**ESR improvement**											
63 (1 RCT)	not serious	not serious	not serious	serious	none	⊕⊕⊕◯ Moderate[c]	16/31 (51.6%)	7/32 (21.9%)	**RR 0.42** (0.20 to 0.89)	516 per 1000	**299 fewer per 1000** (from 413 fewer to 57 fewer)
**Joint score improvement**											

MTX compared to placebo for systemic JIA

Bibliography: Woo P, Southwood TR, Prieur AM, Doré CJ, Grainger J, David J, Ryder C, Hasson N, Hall A, Lemelle I. Randomized, placebo-controlled, crossover trial of low-dose oral methotrexate in children with extended oligoarticular or systemic arthritis. Arthritis Rheum. 2000 Aug;43(8):1849-57. doi: 10.1002/1529-0131(200008)43:8<1849::AID-ANR22>3.0.CO;2-F. PMID: 10943876.

Certainty assessment							Summary of findings				
70 (1 RCT)	not serious	not serious	not serious	very serious[d]	none	⊕⊕◯◯ Low	16/33 (48.5%)	18/37 (48.6%)	**RR 1.00** (0.62 to 1.63)	485 per 1000	**0 fewer per 1000** (from 184 fewer to 305 more)
**Overall clinical improvement**											
88 (1 RCT)	not serious	not serious	not serious	very serious[e]	none	⊕⊕◯◯ Low	11/44 (25.0%)	7/44 (15.9%)	**RR 0.64** (0.27 to 1.49)	250 per 1000	**90 fewer per 1000** (from 183 fewer to 123 more)
**Gastrointestinal upset**											
88 (1 RCT)	not serious	not serious	not serious	very serious[f]	none	⊕⊕◯◯ Low	13/44 (29.5%)	14/44 (31.8%)	**RR 1.08** (0.57 to 2.02)	295 per 1000	**24 more per 1000** (from 127 fewer to 301 more)
**Abnormal aspartate transaminanse levels**											
88 (1 RCT)	not serious	not serious	not serious	very serious[g]	none	⊕⊕◯◯ Low	3/44 (6.8%)	6/44 (13.6%)	**RR 2.00** (0.53 to 7.50)	68 per 1000	**68 more per 1000** (from 32 fewer to 443 more)

CI: confidence interval; **RR:** risk ratio

Explanations

a,b,d,e,f,g. Only one study. 95% CI is wide.

c. Only one study.

Anakinra compared to placebo for systemic JIA

Bibliography: Quartier P, Allantaz F, Cimaz R, Pillet P, Messiaen C, Bardin C, Bossuyt X, Boutten A, Bienvenu J, Duquesne A, Richer O, Chaussabel D, Mogenet A, Banchereau J, Treluyer JM, Landais P, Pascual V. A multicentre, randomised, double-blind, placebo-controlled trial with the interleukin-1 receptor antagonist anakinra in patients with systemic-onset juvenile idiopathic arthritis (ANAJIS trial). Ann Rheum Dis. 2011 May;70(5):747-54. doi: 10.1136/ard.2010.134254. Epub 2010 Dec 20. PMID: 21173013; PMCID: PMC3070271.

Certainty assessment							Summary of findings				
							Study event rates (%)			Anticipated absolute effects	
Participants (studies) Follow-up	Risk of bias	Inconsistency	Indirectness	Imprecision	Publication bias	Overall certainty of evidence	With placebo	With Anakinra	Relative effect (95% CI)	Risk with placebo	Risk difference with Anakinra
**Modified ACR 30 at 1 month**											
24 (1 RCT)	not serious	not serious	not serious	very serious[a]	none	⊕⊕◯◯ Low	1/12 (8.3%)	8/12 (66.7%)	**RR 8.00** (1.17 to 54.50)	83 per 1000	**583 more per 1000** (from 14 more to 1000 more)
**Modified ACR 50 at 1 month**											
24 (1 RCT)	not serious	not serious	not serious	very serious[b]	none	⊕⊕◯◯ Low	0/12 (0.0%)	7/12 (58.3%)	**RR 15.00** (0.95 to 236.42)	0 per 1000	**0 fewer per 1000** (from 0 fewer to 0 fewer)
**Modified ACR 70 at 1 month**											
24 (1 RCT)	not serious	not serious	not serious	very serious[c]	none	⊕⊕◯◯ Low	0/12 (0.0%)	5/12 (41.7%)	**RR 11.00** (0.67 to 179.29)	0 per 1000	**0 fewer per 1000** (from 0 fewer to 0 fewer)
**Modified ACR 90 at 1 month**											
24 (1 RCT)	not serious	not serious	not serious	serious[d]	none	⊕⊕⊕◯ Moderate	0/12 (0.0%)	0/12 (0.0%)	not estimable	0 per 1000	
**Systemic symptoms responder at 1 month**											

Anakinra compared to placebo for systemic JIA

Bibliography: Quartier P, Allantaz F, Cimaz R, Pillet P, Messiaen C, Bardin C, Bossuyt X, Boutten A, Bienvenu J, Duquesne A, Richer O, Chaussabel D, Mogenet A, Banchereau J, Treluyer JM, Landais P, Pascual V. A multicentre, randomised, double-blind, placebo-controlled trial with the interleukin-1 receptor antagonist anakinra in patients with systemic-onset juvenile idiopathic arthritis (ANAJIS trial). Ann Rheum Dis. 2011 May;70(5):747-54. doi: 10.1136/ard.2010.134254. Epub 2010 Dec 20. PMID: 21173013; PMCID: PMC3070271.

Certainty assessment							Summary of findings				
24 (1 RCT)	not serious	not serious	not serious	very serious[e]	none	⊕⊕◯◯ Low	1/12 (8.3%)	8/12 (66.7%)	**RR 8.00** (1.17 to 54.50)	83 per 1000	**583 more per 1000** (from 14 more to 1000 more)
**ACR 30 responders at 1 month**											
24 (1 RCT)	not serious	not serious	not serious	very serious[f]	none	⊕⊕◯◯ Low	7/12 (58.3%)	11/12 (91.7%)	**RR 1.57** (0.95 to 2.61)	583 per 1000	**333 more per 1000** (from 29 fewer to 939 more)
**ACR 30 and no fever responders at 1 month**											
24 (1 RCT)	not serious	not serious	not serious	very serious[g]	none	⊕⊕◯◯ Low	6/12 (50.0%)	11/12 (91.7%)	**RR 1.83** (1.02 to 3.31)	500 per 1000	**415 more per 1000** (from 10 more to 1000 more)
**ACR 30, no fever and CRP <15 mg/L responders at 1 month**											
24 (1 RCT)	not serious	not serious	not serious	very serious[h]	none	⊕⊕◯◯ Low	3/12 (25.0%)	10/12 (83.3%)	**RR 3.33** (1.21 to 9.17)	250 per 1000	**583 more per 1000** (from 52 more to 1000 more)
**Pain to injection at 1 month**											
24 (1 RCT)	not serious	not serious	not serious	very serious[i]	none	⊕⊕◯◯ Low	6/12 (50.0%)	8/12 (66.7%)	**RR 1.33** (0.67 to 2.67)	500 per 1000	**165 more per 1000** (from 165 fewer to 835 more)

Anakinra compared to placebo for systemic JIA

Bibliography: Quartier P, Allantaz F, Cimaz R, Pillet P, Messiaen C, Bardin C, Bossuyt X, Boutten A, Bienvenu J, Duquesne A, Richer O, Chaussabel D, Mogenet A, Banchereau J, Treluyer JM, Landais P, Pascual V. A multicentre, randomised, double-blind, placebo-controlled trial with the interleukin-1 receptor antagonist anakinra in patients with systemic-onset juvenile idiopathic arthritis (ANAJIS trial). Ann Rheum Dis. 2011 May;70(5):747-54. doi: 10.1136/ard.2010.134254. Epub 2010 Dec 20. PMID: 21173013; PMCID: PMC3070271.

Certainty assessment							Summary of findings				
**Post/injection erythema at 1 month**											
24<br>(1 RCT)	not serious	not serious	not serious	very serious[j]	none	⨁⨁◯◯<br>Low	1/12<br>(8.3%)	3/12<br>(25.0%)	**RR 3.00**<br>(0.36 to 24.92)	83 per 1000	**167 more per 1000**<br>(from 53 fewer to 1000 more)

CI: confidence interval; **RR:** risk ratio

Explanations

a,b,c,e,f,g,h,i,j. Only one study. 95% is wide.

d. Only one study.

Anakinra (first line) compared to Anakinra (second line) for systemic JIA

Bibliography: Atemnkeng Ntam V, Klein A, Horneff G. Safety and efficacy of anakinra as first-line or second-line therapy for systemic onset juvenile idiopathic arthritis - data from the German BIKER registry. Expert Opin Drug Saf. 2021 Jan;20(1):93-100. doi: 10.1080/14740338.2021.1843631. Epub 2020 Nov 18. PMID: 33148061.

Certainty assessment							Summary of findings				
Participants (studies) Follow-up	Risk of bias	Inconsistency	Indirectness	Imprecision	Publication bias	Overall certainty of evidence	Study event rates (%)		Relative effect (95% CI)	Anticipated absolute effects	
							With Anakinra (second line)	With Anakinra (first line)		Risk with Anakinra (second line)	Risk difference with Anakinra (first line)
**JADAS MDA**											
51 (1 observational study)	not serious	not serious	not serious	serious[a]	none	⨁◯◯◯ Very low	25/40 (62.5%)	7/11 (63.6%)	**RR 1.02** (0.61 to 1.69)	625 per 1000	**13 more per 1000** (from 244 fewer to 431 more)
**JADAS remission**											
51 (1 observational study)	not serious	not serious	not serious	very serious[b]	none	⨁◯◯◯ Very low	18/40 (45.0%)	6/11 (54.5%)	**RR 1.21** (0.64 to 2.30)	450 per 1000	**94 more per 1000** (from 162 fewer to 585 more)
**Inactive disease**											
51 (1 observational study)	not serious	not serious	not serious	very serious[c]	none	⨁◯◯◯ Very low	28/40 (70.0%)	6/11 (54.5%)	**RR 0.78** (0.44 to 1.39)	700 per 1000	**154 fewer per 1000** (from 392 fewer to 273 more)
**CHAQ,change**											

Anakinra (first line) compared to Anakinra (second line) for systemic JIA

Bibliography: Atemnkeng Ntam V, Klein A, Horneff G. Safety and efficacy of anakinra as first-line or second-line therapy for systemic onset juvenile idiopathic arthritis - data from the German BIKER registry. Expert Opin Drug Saf. 2021 Jan;20(1):93-100. doi: 10.1080/14740338.2021.1843631. Epub 2020 Nov 18. PMID: 33148061.

Certainty assessment							Summary of findings				
51 (1 observational study)	not serious	not serious	not serious	serious[d]	none	⊕◯◯◯ Very low	40	11	-	The mean cHAQ,change was **0**	MD **0.2 higher** (0.39 lower to 0.79 higher)

Serious AE

51 (1 observational study)	not serious	not serious	not serious	very serious[e]	none	⊕◯◯◯ Very low	1/40 (2.5%)	0/11 (0.0%)	**RR 1.14** (0.05 to 26.19)	25 per 1000	**3 more per 1000** (from 24 fewer to 630 more)

Discontinuation

51 (1 observational study)	not serious	not serious	not serious	serious[f]	none	⊕◯◯◯ Very low	20/40 (50.0%)	9/11 (81.8%)	**RR 1.64** (1.08 to 2.48)	500 per 1000	**320 more per 1000** (from 40 more to 740 more)

CI: confidence interval; **MD:** mean difference; **RR:** risk ratio

Explanations

a,b,c,e. Only one study. 95% is wide.

d,f. Only one study.

Tocilizumab compared to placebo for systemic JIA

Bibliography: De Benedetti F, Brunner HI, Ruperto N, Kenwright A, Wright S, Calvo I, Cuttica R, Ravelli A, Schneider R, Woo P, Wouters C, Xavier R, Zemel L, Baildam E, Burgos-Vargas R, Dolezalova P, Garay SM, Merino R, Joos R, Grom A, Wulffraat N, Zuber Z, Zulian F, Lovell D, Martini A; PRINTO; PRCSG. Randomized trial of tocilizumab in systemic juvenile idiopathic arthritis. N Engl J Med. 2012 Dec 20;367(25):2385-95. doi: 10.1056/NEJMoa1112802.

Yokota S, Imagawa T, Mori M, Miyamae T, Aihara Y, Takei S, Iwata N, Umebayashi H, Murata T, Miyoshi M, Tomiita M, Nishimoto N, Kishimoto T. Efficacy and safety of tocilizumab in patients with systemic-onset juvenile idiopathic arthritis: a randomised, double-blind, placebo-controlled, withdrawal phase III trial. Lancet. 2008 Mar 22;371(9617):998-1006. doi: 10.1016/S0140-6736(08)60454-7. PMID: 18358927.

Certainty assessment							Summary of findings				
Participants (studies) Follow-up	Risk of bias	Inconsistency	Indirectness	Imprecision	Publication bias	Overall certainty of evidence	Study event rates (%)		Relative effect (95% CI)	Anticipated absolute effects	
							With placebo	With Tocilizumab		Risk with placebo	Risk difference with Tocilizumab
**JIA ACR30 response at week 12**											
155 (2 RCTs)	not serious	not serious	not serious	serious[a]	none	⨁⨁⨁◯ Moderate	13/60 (21.7%)	84/95 (88.4%)	**RR 3.93** (2.42 to 6.40)	217 per 1000	**635 more per 1000** (from 308 more to 1000 more)
**JIA ACR50 response at week 12**											
155 (2 RCTs)	not serious	not serious	not serious	serious[b]	none	⨁⨁⨁◯ Moderate	8/60 (13.3%)	80/95 (84.2%)	**RR 6.54** (3.31 to 12.92)	133 per 1000	**739 more per 1000** (from 308 more to 1000 more)
**JIA ACR70 response at week 12**											
155 (2 RCTs)	not serious	not serious	not serious	serious[c]	none	⨁⨁⨁◯ Moderate	6/60 (10.0%)	68/95 (71.6%)	**RR 7.50** (3.38 to 16.65)	100 per 1000	**650 more per 1000** (from 238 more to 1000 more)
**JIA ACR90 response at week 12**											

Tocilizumab compared to placebo for systemic JIA

Bibliography: De Benedetti F, Brunner HI, Ruperto N, Kenwright A, Wright S, Calvo I, Cuttica R, Ravelli A, Schneider R, Woo P, Wouters C, Xavier R, Zemel L, Baildam E, Burgos-Vargas R, Dolezalova P, Garay SM, Merino R, Joos R, Grom A, Wulffraat N, Zuber Z, Zulian F, Lovell D, Martini A; PRINTO; PRCSG. Randomized trial of tocilizumab in systemic juvenile idiopathic arthritis. N Engl J Med. 2012 Dec 20;367(25):2385-95. doi: 10.1056/NEJMoa1112802.

Yokota S, Imagawa T, Mori M, Miyamae T, Aihara Y, Takei S, Iwata N, Umebayashi H, Murata T, Miyoshi M, Tomiita M, Nishimoto N, Kishimoto T. Efficacy and safety of tocilizumab in patients with systemic-onset juvenile idiopathic arthritis: a randomised, double-blind, placebo-controlled, withdrawal phase III trial. Lancet. 2008 Mar 22;371(9617):998-1006. doi: 10.1016/S0140-6736(08)60454-7. PMID: 18358927.

Certainty assessment							Summary of findings				
112 (1 RCT)	not serious	not serious	not serious	serious[d]	none	⊕⊕⊕◯ Moderate	2/37 (5.4%)	28/75 (37.3%)	**RR 6.91** (1.74 to 27.44)	54 per 1000	**319 more per 1000** (from 40 more to 1000 more)

Patients with fever due to sJIA at baseline but with no fever at week 12

112 (1 RCT)	not serious	not serious	not serious	serious[e]	none	⊕⊕⊕◯ Moderate	8/37 (21.6%)	64/75 (85.3%)	**RR 3.95** (2.12 to 7.34)	216 per 1000	**638 more per 1000** (from 242 more to 1000 more)

Patients with elevated CRP at baseline and normal CRP at week 12,

112 (1 RCT)	not serious	not serious	not serious	serious[f]	none	⊕⊕⊕◯ Moderate	2/37 (5.4%)	74/75 (98.7%)	**RR 18.25** (4.74 to 70.28)	54 per 1000	**932 more per 1000** (from 202 more to 1000 more)

Patients with anemia at baseline and normal Hb level at week 12

112 (1 RCT)	not serious	not serious	not serious	serious[g]	none	⊕⊕⊕◯ Moderate	1/37 (2.7%)	66/75 (88.0%)	**RR 32.56** (4.70 to 225.47)	27 per 1000	**853 more per 1000** (from 100 more to 1000 more)

Patients with rash characteristic of sJIA at baseline but no rash at week 12

Tocilizumab compared to placebo for systemic JIA

Bibliography: De Benedetti F, Brunner HI, Ruperto N, Kenwright A, Wright S, Calvo I, Cuttica R, Ravelli A, Schneider R, Woo P, Wouters C, Xavier R, Zemel L, Baildam E, Burgos-Vargas R, Dolezalova P, Garay SM, Merino R, Joos R, Grom A, Wulffraat N, Zuber Z, Zulian F, Lovell D, Martini A; PRINTO; PRCSG. Randomized trial of tocilizumab in systemic juvenile idiopathic arthritis. N Engl J Med. 2012 Dec 20;367(25):2385-95. doi: 10.1056/NEJMoa1112802.

Yokota S, Imagawa T, Mori M, Miyamae T, Aihara Y, Takei S, Iwata N, Umebayashi H, Murata T, Miyoshi M, Tomiita M, Nishimoto N, Kishimoto T. Efficacy and safety of tocilizumab in patients with systemic-onset juvenile idiopathic arthritis: a randomised, double-blind, placebo-controlled, withdrawal phase III trial. Lancet. 2008 Mar 22;371(9617):998-1006. doi: 10.1016/S0140-6736(08)60454-7. PMID: 18358927.

Certainty assessment							Summary of findings				
112 (1 RCT)	not serious	not serious	not serious	serious[h]	none	⊕⊕⊕◯ Moderate	4/37 (10.8%)	48/75 (64.0%)	**RR 5.92** (2.31 to 15.17)	108 per 1000	**532 more per 1000** (from 142 more to 1000 more)

Patients with minimally important improvement in CHAQ-DI by week 12

112 (1 RCT)	not serious	not serious	not serious	serious[i]	none	⊕⊕⊕◯ Moderate	3/37 (8.1%)	60/75 (80.0%)	**RR 9.87** (3.32 to 29.36)	81 per 1000	**719 more per 1000** (from 188 more to 1000 more)

Patients with thrombocytosis at baseline and normal platelet count at week 12

112 (1 RCT)	not serious	not serious	not serious	serious[j]	none	⊕⊕⊕◯ Moderate	2/37 (5.4%)	68/75 (90.7%)	**RR 16.77** (4.35 to 64.69)	54 per 1000	**852 more per 1000** (from 181 more to 1000 more)

Patients with leukocytosis at baseline and normal total WBC count at week 12

112 (1 RCT)	not serious	not serious	not serious	serious[k]	none	⊕⊕⊕◯ Moderate	3/37 (8.1%)	56/75 (74.7%)	**RR 9.21** (3.09 to 27.46)	81 per 1000	**666 more per 1000** (from 169 more to 1000 more)

Patients with minimally important improvement in CHAQ-DI by week 12

Tocilizumab compared to placebo for systemic JIA

Bibliography: De Benedetti F, Brunner HI, Ruperto N, Kenwright A, Wright S, Calvo I, Cuttica R, Ravelli A, Schneider R, Woo P, Wouters C, Xavier R, Zemel L, Baildam E, Burgos-Vargas R, Dolezalova P, Garay SM, Merino R, Joos R, Grom A, Wulffraat N, Zuber Z, Zulian F, Lovell D, Martini A; PRINTO; PRCSG. Randomized trial of tocilizumab in systemic juvenile idiopathic arthritis. N Engl J Med. 2012 Dec 20;367(25):2385-95. doi: 10.1056/NEJMoa1112802.

Yokota S, Imagawa T, Mori M, Miyamae T, Aihara Y, Takei S, Iwata N, Umebayashi H, Murata T, Miyoshi M, Tomiita M, Nishimoto N, Kishimoto T. Efficacy and safety of tocilizumab in patients with systemic-onset juvenile idiopathic arthritis: a randomised, double-blind, placebo-controlled, withdrawal phase III trial. Lancet. 2008 Mar 22;371(9617):998-1006. doi: 10.1016/S0140-6736(08)60454-7. PMID: 18358927.

Certainty assessment							Summary of findings				
112 (1 RCT)	not serious	not serious	not serious	serious[l]	none	⊕⊕⊕◯ Moderate	7/37 (18.9%)	58/75 (77.3%)	**RR 4.09** (2.07 to 8.05)	189 per 1000	**585 more per 1000** (from 202 more to 1000 more)
**Serious adverse event at week 12**											
112 (1 RCT)	not serious	not serious	not serious	serious[m]	none	⊕⊕⊕◯ Moderate	0/37 (0.0%)	4/75 (5.3%)	**RR 4.50** (0.25 to 81.43)	0 per 1000	**0 fewer per 1000** (from 0 fewer to 0 fewer)
**Infection at week 12**											
112 (1 RCT)	not serious	not serious	not serious	serious[n]	none	⊕⊕⊕◯ Moderate	15/37 (40.5%)	60/75 (80.0%)	**RR 1.97** (1.31 to 2.96)	405 per 1000	**393 more per 1000** (from 126 more to 795 more)
**CHAQ at week 12**											
112 (1 RCT)	not serious	not serious	not serious	not serious	none	⊕⊕⊕⊕ High	37	75	-	The mean CHAQ at week 12 was **0**	MD **0.23 lower** (0.59 lower to 0.13 higher)

CI: confidence interval; **MD:** mean difference; **RR:** risk ratio

Explanations

a-n. 95% is wide.

Tocilizumab (Q2W) compared to Tocilizumab (Q4W) for systemic JIA

Bibliography: Kostik MM, Dubko MF, Masalova VV, Snegireva LS, Kornishina TL, Chikova IA, Isupova EA, Kuchinskaya EM, Glebova NI, Buchinskaya NV, Kalashnikova OV, Chasnyk VG. Successful treatment with tocilizumab every 4 weeks of a low disease activity group who achieve a drug-free remission in patients with systemic-onset juvenile idiopathic arthritis. Pediatr Rheumatol Online J. 2015 Jan 23;13:4. doi: 10.1186/1546-0096-13-4. PMID: 25685108; PMCID: PMC4328569.

Certainty assessment							Summary of findings				
Participants (studies) Follow-up	Risk of bias	Inconsistency	Indirectness	Imprecision	Publication bias	Overall certainty of evidence	Study event rates (%)		Relative effect (95% CI)	Anticipated absolute effects	
							With Tocilizumab (Q4W)	With Tocilizumab (Q2W)		Risk with Tocilizumab (Q4W)	Risk difference with Tocilizumab (Q2W)
**Corticosteroids discontinuation**											
26 (1 observational study)	not serious	not serious	not serious	serious[a]	strong association	⨁⨁◯◯ Low	19/19 (100.0%)	2/7 (28.6%)	**RR 0.32** (0.11 to 0.90)	1000 per 1000	**680 fewer per 1000** (from 890 fewer to 100 fewer)
**Methotrexate discontinuation due to remission of SoJIA**											
32 (1 observational study)	not serious	not serious	not serious	very serious	none	⨁◯◯◯ Very low[b]	8/24 (33.3%)	1/8 (12.5%)	**RR 0.38** (0.06 to 2.55)	333 per 1000	**207 fewer per 1000** (from 313 fewer to 517 more)
**SoJIA relapses**											
35 (1 observational study)	not serious	not serious	not serious	very serious[c]	none	⨁◯◯◯ Very low	2/24 (8.3%)	5/11 (45.5%)	**RR 5.45** (1.25 to 23.88)	83 per 1000	**371 more per 1000** (from 21 more to 1000 more)
**Inactive disease**											

Tocilizumab (Q2W) compared to Tocilizumab (Q4W) for systemic JIA

Bibliography: Kostik MM, Dubko MF, Masalova VV, Snegireva LS, Kornishina TL, Chikova IA, Isupova EA, Kuchinskaya EM, Glebova NI, Buchinskaya NV, Kalashnikova OV, Chasnyk VG. Successful treatment with tocilizumab every 4 weeks of a low disease activity group who achieve a drug-free remission in patients with systemic-onset juvenile idiopathic arthritis. Pediatr Rheumatol Online J. 2015 Jan 23;13:4. doi: 10.1186/1546-0096-13-4. PMID: 25685108; PMCID: PMC4328569.

Certainty assessment							Summary of findings				
25 (1 observational study)	not serious	not serious	not serious	serious[d]	none	⨁◯◯◯ Very low	12/25 (48.0%)	0/0	not estimable	480 per 1000	

CI: confidence interval; **RR:** risk ratio

Explanations

a,d. Only one study.

b,c. Only one study. 95% CI is wide.

Canakinumab compared to placebo for systemic JIA

Bibliography: Ruperto N, Brunner HI, Quartier P, Constantin T, Wulffraat N, Horneff G, Brik R, McCann L, Kasapcopur O, Rutkowska-Sak L, Schneider R, Berkun Y, Calvo I, Erguven M, Goffin L, Hofer M, Kallinich T, Oliveira SK, Uziel Y, Viola S, Nistala K, Wouters C, Cimaz R, Ferrandiz MA, Flato B, Gamir ML, Kone-Paut I, Grom A, Magnusson B, Ozen S, Sztajnbok F, Lheritier K, Abrams K, Kim D, Martini A, Lovell DJ; PRINTO; PRCSG. Two randomized trials of canakinumab in systemic juvenile idiopathic arthritis. N Engl J Med. 2012 Dec 20;367(25):2396-406. doi: 10.1056/NEJMoa1205099. PMID: 23252526.

Certainty assessment							Summary of findings				
							Study event rates (%)			Anticipated absolute effects	
Participants (studies) Follow-up	Risk of bias	Inconsistency	Indirectness	Imprecision	Publication bias	Overall certainty of evidence	With placebo	With Canakinumab	Relative effect (95% CI)	Risk with placebo	Risk difference with Canakinumab
**ACR30**											
84 (1 RCT)	not serious	not serious	not serious	very serious[a]	none	⊕⊕◯◯ Low	4/41 (9.8%)	36/43 (83.7%)	**RR 8.58** (3.35 to 21.97)	98 per 1000	**740 more per 1000** (from 229 more to 1000 more)
**ACR50**											
84 (1 RCT)	not serious	not serious	not serious	very serious[b]	none	⊕⊕◯◯ Low	2/41 (4.9%)	29/43 (67.4%)	**RR 13.83** (3.52 to 54.27)	49 per 1000	**626 more per 1000** (from 123 more to 1000 more)
**ACR70**											
84 (1 RCT)	not serious	not serious	not serious	very serious[c]	none	⊕⊕◯◯ Low	1/41 (2.4%)	26/43 (60.5%)	**RR 24.79** (3.52 to 174.41)	24 per 1000	**580 more per 1000** (from 61 more to 1000 more)
**ACR90**											

Canakinumab compared to placebo for systemic JIA

Bibliography: Ruperto N, Brunner HI, Quartier P, Constantin T, Wulffraat N, Horneff G, Brik R, McCann L, Kasapcopur O, Rutkowska-Sak L, Schneider R, Berkun Y, Calvo I, Erguven M, Goffin L, Hofer M, Kallinich T, Oliveira SK, Uziel Y, Viola S, Nistala K, Wouters C, Cimaz R, Ferrandiz MA, Flato B, Gamir ML, Kone-Paut I, Grom A, Magnusson B, Ozen S, Sztajnbok F, Lheritier K, Abrams K, Kim D, Martini A, Lovell DJ; PRINTO; PRCSG. Two randomized trials of canakinumab in systemic juvenile idiopathic arthritis. N Engl J Med. 2012 Dec 20;367(25):2396-406. doi: 10.1056/NEJMoa1205099. PMID: 23252526.

Certainty assessment							Summary of findings				
84 (1 RCT)	not serious	not serious	not serious	very serious[d]	none	⨁⨁◯◯ Low	0/41 (0.0%)	18/43 (41.9%)	**RR 35.32** (2.20 to 567.60)	0 per 1000	**0 fewer per 1000** (from 0 fewer to 0 fewer)
**Inactive Disease**											
84 (1 RCT)	not serious	not serious	not serious	serious[e]	none	⨁⨁⨁◯ Moderate	0/41 (0.0%)	14/43 (32.6%)	**RR 27.68** (1.70 to 449.48)	0 per 1000	**0 fewer per 1000** (from 0 fewer to 0 fewer)
**Adverse Events**											
84 (1 RCT)	not serious	not serious	not serious	serious[f]	none	⨁⨁⨁◯ Moderate	16/41 (39.0%)	24/43 (55.8%)	**RR 1.43** (0.90 to 2.28)	390 per 1000	**168 more per 1000** (from 39 fewer to 500 more)

CI: confidence interval; **RR:** risk ratio

Explanations

a-f. Only one study. 95% CI is wide.

Canakinumab compared to tapering for systemic JIA

Bibliography: Quartier P, Alexeeva E, Constantin T, Chasnyk V, Wulffraat N, Palmblad K, Wouters C, I Brunner H, Marzan K, Schneider R, Horneff G, Martini A, Anton J, Wei X, Slade A, Ruperto N, Abrams K; Paediatric Rheumatology International Trials Organisation and the Pediatric Rheumatology Collaborative Study Group. Tapering Canakinumab Monotherapy in Patients With Systemic Juvenile Idiopathic Arthritis in Clinical Remission: Results From a Phase IIIb/IV Open-Label, Randomized Study. Arthritis Rheumatol. 2021 Feb;73(2):336-346. doi: 10.1002/art.41488. Epub 2020 Dec 11. PMID: 32783351; PMCID: PMC7898684.

Certainty assessment							Summary of findings				
							Study event rates (%)			Anticipated absolute effects	
Participants (studies) Follow-up	Risk of bias	Inconsistency	Indirectness	Imprecision	Publication bias	Overall certainty of evidence	With tapering	With Canakinumab	Relative effect (95% CI)	Risk with tapering	Risk difference with Canakinumab
**Clinical remission week 27**											
75 (1 RCT)	not serious	not serious	not serious	serious[a]	none	⨁⨁⨁◯ Moderate	31/37 (83.8%)	27/38 (71.1%)	**RR 0.85** (0.66 to 1.09)	838 per 1000	**126 fewer per 1000** (from 285 fewer to 75 more)
**Step 1 (every 4 weeks tapering)**											
75 (1 RCT)	not serious	not serious	not serious	serious[b]	none	⨁⨁⨁◯ Moderate	31/37 (83.8%)	27/38 (71.1%)	**RR 0.85** (0.66 to 1.09)	838 per 1000	**126 fewer per 1000** (from 285 fewer to 75 more)
**Step 2 (every 8 weeks tapering)**											
75 (1 RCT)	not serious	not serious	not serious	serious[c]	none	⨁⨁⨁◯ Moderate	30/37 (81.1%)	26/38 (68.4%)	**RR 0.84** (0.65 to 1.10)	811 per 1000	**130 fewer per 1000** (from 284 fewer to 81 more)
**Step 3 (every 12 weeks tapering)**											

Canakinumab compared to tapering for systemic JIA

Bibliography: Quartier P, Alexeeva E, Constantin T, Chasnyk V, Wulffraat N, Palmblad K, Wouters C, I Brunner H, Marzan K, Schneider R, Horneff G, Martini A, Anton J, Wei X, Slade A, Ruperto N, Abrams K; Paediatric Rheumatology International Trials Organisation and the Pediatric Rheumatology Collaborative Study Group. Tapering Canakinumab Monotherapy in Patients With Systemic Juvenile Idiopathic Arthritis in Clinical Remission: Results From a Phase IIIb/IV Open-Label, Randomized Study. Arthritis Rheumatol. 2021 Feb;73(2):336-346. doi: 10.1002/art.41488. Epub 2020 Dec 11. PMID: 32783351; PMCID: PMC7898684.

Certainty assessment							Summary of findings				
75 (1 RCT)	not serious	not serious	not serious	serious[d]	none	⨁⨁⨁◯ Moderate	8/37 (21.6%)	17/38 (44.7%)	**RR 2.07** (1.02 to 4.20)	216 per 1000	**231 more per 1000** (from 4 more to 692 more)

CI: confidence interval; **RR:** risk ratio

Explanations

a-d. Only one study. 95% CI is wide.

Canakinumab (naive) compared to switching to Canakinumab from Tocilizumab for systemic JIA

Bibliography: Alexeeva E, Dvoryakovskaya T, Isaeva K, et alFRI0553 CANAKINUMAB AS A FIRST-LINE AND SECOND-LINE BIOLOGIC FOR TREATMENT OF SYSTEMIC JUVENILE IDIOPATHIC ARTHRITIS IN CHILDREN UNDER 4 YEARS OF AGEAnnals of the Rheumatic Diseases 2019;78:972.

Certainty assessment							Summary of findings				
							Study event rates (%)			Anticipated absolute effects	
Participants (studies) Follow-up	Risk of bias	Inconsistency	Indirectness	Imprecision	Publication bias	Overall certainty of evidence	With switching to Canakinumab from Tocilizumab	With Canakinumab (naive)	Relative effect (95% CI)	Risk with switching to Canakinumab from Tocilizumab	Risk difference with Canakinumab (naive)
**ACR90**											
17 (1 observational study)	not serious	not serious	not serious	very serious[a]	none	⨁◯◯◯ Very low	4/8 (50.0%)	5/9 (55.6%)	**RR 1.11** (0.45 to 2.75)	500 per 1000	**55 more per 1000** (from 275 fewer to 875 more)
**Inactive Disease**											
17 (1 observational study)	not serious	not serious	not serious	very serious[b]	none	⨁◯◯◯ Very low	1/8 (12.5%)	1/9 (11.1%)	**RR 0.89** (0.07 to 12.00)	125 per 1000	**14 fewer per 1000** (from 116 fewer to 1000 more)

CI: confidence interval; **RR:** risk ratio

Explanations

a,b Only one study. 95% CI is wide.

Rilonacept compared to placebo for systemic JIA

Bibliography: Lovell DJ, Giannini EH, Reiff AO, Kimura Y, Li S, Hashkes PJ, Wallace CA, Onel KB, Foell D, Wu R, Biedermann S, Hamilton JD, Radin AR. Long-term safety and efficacy of rilonacept in patients with systemic juvenile idiopathic arthritis. Arthritis Rheum. 2013 Sep;65(9):2486-96. doi: 10.1002/art.38042. PMID: 23754188.

Ilowite NT, Prather K, Lokhnygina Y, Schanberg LE, Elder M, Milojevic D, Verbsky JW, Spalding SJ, Kimura Y, Imundo LF, Punaro MG, Sherry DD, Tarvin SE, Zemel LS, Birmingham JD, Gottlieb BS, Miller ML, O'Neil K, Ruth NM, Wallace CA, Singer NG, Sandborg CI. Randomized, double-blind, placebo-controlled trial of the efficacy and safety of rilonacept in the treatment of systemic juvenile idiopathic arthritis. Arthritis Rheumatol. 2014 Sep;66(9):2570-9. doi: 10.1002/art.38699. PMID: 24839206; PMCID: PMC4314719.

Certainty assessment							Summary of findings				
							Study event rates (%)			Anticipated absolute effects	
Participants (studies) Follow-up	Risk of bias	Inconsistency	Indirectness	Imprecision	Publication bias	Overall certainty of evidence	With placebo	With Rilonacept	Relative effect (95% CI)	Risk with placebo	Risk difference with Rilonacept
**ACR pedi 30 at 4 weeks**											
92 (2 RCTs)	not serious	not serious	not serious	not serious	none	⨁⨁⨁⨁ High	15/40 (37.5%)	32/52 (61.5%)	**RR 1.77** (1.14 to 2.76)	375 per 1000	**289 more per 1000** (from 52 more to 660 more)
**ACR pedi 50 at 4 weeks**											
92 (2 RCTs)	not serious	not serious	not serious	not serious	none	⨁⨁⨁⨁ High	11/40 (27.5%)	26/52 (50.0%)	**RR 1.99** (1.13 to 3.50)	275 per 1000	**272 more per 1000** (from 36 more to 688 more)
**ACR pedi 70 at 4 weeks**											
92 (2 RCTs)	not serious	not serious	not serious	not serious	none	⨁⨁⨁⨁ High	5/40 (12.5%)	17/52 (32.7%)	**RR 2.77** (1.14 to 6.75)	125 per 1000	**221 more per 1000** (from 17 more to 719 more)
**Inactive disease at 4 weeks**											

Rilonacept compared to placebo for systemic JIA

Bibliography: Lovell DJ, Giannini EH, Reiff AO, Kimura Y, Li S, Hashkes PJ, Wallace CA, Onel KB, Foell D, Wu R, Biedermann S, Hamilton JD, Radin AR. Long-term safety and efficacy of rilonacept in patients with systemic juvenile idiopathic arthritis. Arthritis Rheum. 2013 Sep;65(9):2486-96. doi: 10.1002/art.38042. PMID: 23754188.

Ilowite NT, Prather K, Lokhnygina Y, Schanberg LE, Elder M, Milojevic D, Verbsky JW, Spalding SJ, Kimura Y, Imundo LF, Punaro MG, Sherry DD, Tarvin SE, Zemel LS, Birmingham JD, Gottlieb BS, Miller ML, O'Neil K, Ruth NM, Wallace CA, Singer NG, Sandborg CI. Randomized, double-blind, placebo-controlled trial of the efficacy and safety of rilonacept in the treatment of systemic juvenile idiopathic arthritis. Arthritis Rheumatol. 2014 Sep;66(9):2570-9. doi: 10.1002/art.38699. PMID: 24839206; PMCID: PMC4314719.

Certainty assessment							Summary of findings				
70<br>(1 RCT)	not serious	not serious	not serious	very serious[a]	none	⊕⊕◯◯<br>Low	0/34<br>(0.0%)	2/36<br>(5.6%)	**RR 4.73**<br>(0.24 to 95.09)	0 per 1000	**0 fewer per 1000**<br>(from 0 fewer to 0 fewer)

Fever at 4 weeks

70<br>(1 RCT)	not serious	not serious	not serious	very serious[b]	none	⊕⊕◯◯<br>Low	5/34<br>(14.7%)	3/36<br>(8.3%)	**RR 0.57**<br>(0.15 to 2.19)	147 per 1000	**63 fewer per 1000**<br>(from 125 fewer to 175 more)

Rash at 4 weeks

70<br>(1 RCT)	not serious	not serious	not serious	very serious[c]	none	⊕⊕◯◯<br>Low	8/34<br>(23.5%)	3/36<br>(8.3%)	**RR 0.35**<br>(0.10 to 1.23)	235 per 1000	**153 fewer per 1000**<br>(from 212 fewer to 54 more)

No. of joints with active arthritis at 4 weeks

72<br>(1 RCT)	not serious	not serious	not serious	serious[d]	none	⊕⊕⊕◯<br>Moderate	36	36	-	The mean no. of joints with active arthritis at 4 weeks was **0**	MD **5.5 lower**<br>(9.03 lower to 1.97 lower)

No. of joints with limited ROM at 4 weeks

Rilonacept compared to placebo for systemic JIA

Bibliography: Lovell DJ, Giannini EH, Reiff AO, Kimura Y, Li S, Hashkes PJ, Wallace CA, Onel KB, Foell D, Wu R, Biedermann S, Hamilton JD, Radin AR. Long-term safety and efficacy of rilonacept in patients with systemic juvenile idiopathic arthritis. Arthritis Rheum. 2013 Sep;65(9):2486-96. doi: 10.1002/art.38042. PMID: 23754188.

Ilowite NT, Prather K, Lokhnygina Y, Schanberg LE, Elder M, Milojevic D, Verbsky JW, Spalding SJ, Kimura Y, Imundo LF, Punaro MG, Sherry DD, Tarvin SE, Zemel LS, Birmingham JD, Gottlieb BS, Miller ML, O'Neil K, Ruth NM, Wallace CA, Singer NG, Sandborg CI. Randomized, double-blind, placebo-controlled trial of the efficacy and safety of rilonacept in the treatment of systemic juvenile idiopathic arthritis. Arthritis Rheumatol. 2014 Sep;66(9):2570-9. doi: 10.1002/art.38699. PMID: 24839206; PMCID: PMC4314719.

Certainty assessment							Summary of findings				
72<br>(1 RCT)	not serious	not serious	not serious	serious[e]	none	⊕⊕⊕◯<br>Moderate	36	36	-	The mean no. of joints with limited ROM at 4 weeks was **0**	MD **4.5 lower** (7.39 lower to 1.61 lower)

Physician's global assessment of disease activity at 4 weeks

72<br>(1 RCT)	not serious	not serious	not serious	serious[f]	none	⊕⊕⊕◯<br>Moderate	36	36	-	The mean physician's global assessment of disease activity at 4 weeks was **0**	MD **23.67 lower** (35.91 lower to 11.43 lower)

Parent's global assessment of overall well-being at 4 weeks

72<br>(1 RCT)	not serious	not serious	not serious	serious[g]	none	⊕⊕⊕◯<br>Moderate	36	36	-	The mean parent's global assessment of overall well-being at 4 weeks was **0**	MD **26 lower** (39.47 lower to 12.53 lower)

CHAQ-DI score at 4 weeks

Rilonacept compared to placebo for systemic JIA

Bibliography: Lovell DJ, Giannini EH, Reiff AO, Kimura Y, Li S, Hashkes PJ, Wallace CA, Onel KB, Foell D, Wu R, Biedermann S, Hamilton JD, Radin AR. Long-term safety and efficacy of rilonacept in patients with systemic juvenile idiopathic arthritis. Arthritis Rheum. 2013 Sep;65(9):2486-96. doi: 10.1002/art.38042. PMID: 23754188.

Ilowite NT, Prather K, Lokhnygina Y, Schanberg LE, Elder M, Milojevic D, Verbsky JW, Spalding SJ, Kimura Y, Imundo LF, Punaro MG, Sherry DD, Tarvin SE, Zemel LS, Birmingham JD, Gottlieb BS, Miller ML, O'Neil K, Ruth NM, Wallace CA, Singer NG, Sandborg CI. Randomized, double-blind, placebo-controlled trial of the efficacy and safety of rilonacept in the treatment of systemic juvenile idiopathic arthritis. Arthritis Rheumatol. 2014 Sep;66(9):2570-9. doi: 10.1002/art.38699. PMID: 24839206; PMCID: PMC4314719.

Certainty assessment							Summary of findings				
72 (1 RCT)	not serious	not serious	not serious	serious[h]	none	⊕⊕⊕◯ Moderate	36	36	-	The mean CHAQ-DI score at 4 weeks was **0**	MD **0.44 lower** (0.85 lower to 0.03 lower)

C-reactive protein, mg/dl at 4 weeks

72 (1 RCT)	not serious	not serious	not serious	serious[i]	none	⊕⊕⊕◯ Moderate	36	36	-	The mean c-reactive protein, mg/dl at 4 weeks was **0**	MD **3.5 lower** (5.3 lower to 1.7 lower)

Ferritin, ng/ml at 4 weeks

72 (1 RCT)	not serious	not serious	not serious	serious[j]	none	⊕⊕⊕◯ Moderate	36	36	-	The mean ferritin, ng/ml at 4 weeks was **0**	MD **49.83 lower** (89.86 lower to 9.8 lower)

ESR, mm/hour at 4 weeks

72 (1 RCT)	not serious	not serious	not serious	serious[k]	none	⊕⊕⊕◯ Moderate	36	36	-	The mean ESR, mm/hour at 4 weeks was **0**	MD **25 lower** (39.25 lower to 10.75 lower)

Adverse event at 4 weeks

Rilonacept compared to placebo for systemic JIA

Bibliography: Lovell DJ, Giannini EH, Reiff AO, Kimura Y, Li S, Hashkes PJ, Wallace CA, Onel KB, Foell D, Wu R, Biedermann S, Hamilton JD, Radin AR. Long-term safety and efficacy of rilonacept in patients with systemic juvenile idiopathic arthritis. Arthritis Rheum. 2013 Sep;65(9):2486-96. doi: 10.1002/art.38042. PMID: 23754188.

Ilowite NT, Prather K, Lokhnygina Y, Schanberg LE, Elder M, Milojevic D, Verbsky JW, Spalding SJ, Kimura Y, Imundo LF, Punaro MG, Sherry DD, Tarvin SE, Zemel LS, Birmingham JD, Gottlieb BS, Miller ML, O'Neil K, Ruth NM, Wallace CA, Singer NG, Sandborg CI. Randomized, double-blind, placebo-controlled trial of the efficacy and safety of rilonacept in the treatment of systemic juvenile idiopathic arthritis. Arthritis Rheumatol. 2014 Sep;66(9):2570-9. doi: 10.1002/art.38699. PMID: 24839206; PMCID: PMC4314719.

Certainty assessment							Summary of findings				
72 (1 RCT)	not serious	not serious	not serious	serious[l]	none	⊕⊕⊕◯ Moderate	19/36 (52.8%)	10/36 (27.8%)	**RR 0.53** (0.29 to 0.97)	528 per 1000	**248 fewer per 1000** (from 375 fewer to 16 fewer)

Serious adverse event at 4 weeks

72 (1 RCT)	not serious	not serious	not serious	very serious[m]	none	⊕⊕◯◯ Low	1/36 (2.8%)	1/36 (2.8%)	**RR 1.00** (0.07 to 15.38)	28 per 1000	**0 fewer per 1000** (from 26 fewer to 399 more)

Infection event at 4 weeks

72 (1 RCT)	not serious	not serious	not serious	very serious[n]	none	⊕⊕◯◯ Low	2/36 (5.6%)	2/36 (5.6%)	**RR 1.00** (0.15 to 6.72)	56 per 1000	**0 fewer per 1000** (from 47 fewer to 318 more)

CI: confidence interval; **MD:** mean difference; **RR:** risk ratio

Explanations

a,b,c,m,n. Only one study. 95% CI is wide.

d-l.Only one study.
