# Supplementary material for: Pan American League of Associations for Rheumatology treatment recommendations for systemic juvenile idiopathic arthritis
Source: Rheumatol Adv Pract. 2025 Nov 11;9(4):rkaf087. doi: 10.1093/rap/rkaf087 (PMC12607261; doi:10.1093/rap/rkaf087)
Supplement: rkaf087_Supplementary_Data [file rkaf087_supplementary_data.zip › Supplementary Data 4. Systemic JIA Recommendations with references 23_05_2025_ clean version.docx]

***Recommendation 1:***

**For patients with sJIA who present predominantly with systemic features and have high disease activity, initiate high-dose intravenous methylprednisolone (pulse therapy) rather than oral prednisone at 1 mg/kg/day.**

High-dose intravenous (IV) methylprednisolone (“pulse therapy”) may be needed for very sick patients (high persistent fever, severe anaemia, myocarditis or pericarditis, and MAS),^[[1]](#endnote-1)^ due to its rapid onset of action. The recommended dose is 10-30 mg/kg/dose (maximum dose 1 g) for 3 to 5 consecutive days.^[[2]](#endnote-2)^

In sJIA patients with predominantly systemic features but without high disease activity, treatment with oral prednisone or equivalent, in a dose of (0.5-1 mg/kg/day, maximum dose 60 mg/day) may be considered, with tapering as soon as partial response is achieved and further withdrawal, based on a case-by-case evaluation,^[[3]](#endnote-3)^ as shown in **Figure 2** (algorithm for sJIA pharmacological treatment for patients with predominantly systemic features).

***Recommendation 2:***

**For patients with newly diagnosed sJIA presenting predominantly with systemic features** **and have high disease activity, initiate treatment with a biologic DMARD targeting the IL-1 pathway (anakinra, canakinumab, or rilonacept) or the IL-6 receptor (tocilizumab) as first-line therapy.**

Glucocorticoid therapy should be limited to the lowest effective dose for the shortest duration. To achieve a complete response and reduce steroid-related damage, adding a biologic disease-modifying antirheumatic drug (bDMARD) is recommended.

Evidence shows that targeting the IL-1 pathway in new-onset SJIA with predominant systemic signs lead to better outcomes, enabling lower glucocorticoid doses, shorter treatment durations, and improved long-term outcomes.^[[4]](#endnote-4),^^[[5]](#endnote-5),^^[[6]](#endnote-6),^^[[7]](#endnote-7),^^[[8]](#endnote-8)^

Initiating IL-1-targeted therapy as first-line therapy is particularly effective in untreated, new-onset cases. ^[[9]](#endnote-9)^ Both anakinra and canakinumab have demonstrated favourable outcomes with low systemic adverse effects, although cases of MAS have been reported with canakinumab.^4,5,6,7,^^[[10]](#endnote-10)^

Few trials have compared rilonacept to placebo^.^^[[11]](#endnote-11),^^[[12]](#endnote-12)^ It is worth mentioning that rilonacept is unavailable in most Latin American (LATAM) countries. Moreover, in LATAM, IL-1-targeted therapies is often limited due to cost and regulatory issues.^[[13]](#endnote-13)^

Similarly, studies on tocilizumab report significant improvements in sJIA patients, including reduced disease activity, fever resolution, and long-term efficacy. Many patients discontinued glucocorticoids with sustained improvement, especially when treatment began early in the disease course.^[[14]](#endnote-14),^^[[15]](#endnote-15),^^[[16]](#endnote-16),^^[[17]](#endnote-17),^^[[18]](#endnote-18),^^[[19]](#endnote-19)^

Subcutaneous tocilizumab offers similar efficacy to intravenous administration, with added convenience for patients and caregivers, particularly for home use.^[[20]](#endnote-20)^

However, when bDMARDs are unavailable, the use of a non-biologic DMARDs may be considered. Although the expert panel did not develop a specific research questions structured using the PICO format for this drug, cyclosporine A may be an option in this scenario, however its slower onset of action should be considered. It has demonstrated favourable outcomes, especially fever control and a significant steroid-sparing effect.^[[21]](#endnote-21),^^[[22]](#endnote-22),^^[[23]](#endnote-23)^  On the other hand, no preferred agent has been defined between IL-1 and IL-6 targeted therapies, due to the lack of direct comparisons. The choice depends on availability, physician experience, and patient/caregiver preferences. Switching between these agents is appropriate in cases of inefficacy or poor tolerability.^3^

***Recommendation 3:***

**For patients with newly diagnosed sJIA presenting predominantly with active synovitis, initiate treatment with methotrexate.**

Current and previous studies highlight a potential "window of opportunity" for patients with sJIA. Early intervention with cytokine antagonists has proven effective in preventing the expansion of arthritis-inducing T-cell populations. However, in settings where bDMARDs are not readily available due to cost or regulatory constraints, methotrexate (MTX) remains a viable alternative as first-line non-biologic DMARDs. Although MTX may offer some degree of disease control, its efficacy in sJIA is limited due to the unique pathophysiological characteristics of this subtype compared to other forms of JIA.^[[24]](#endnote-24),^^[[25]](#endnote-25)^

Low-dose oral MTX versus placebo was studied by Woo P, et al., who evaluated children with sJIA and extended oligoarthritis, with persistent arthritis for more than one year, many of whom had signs of active systemic disease. When combining data from both subgroups, a significant improvement in disease activity was observed based on assessments by physicians, parents, and patients. There were notable improvements in C-reactive protein (CRP) levels, erythrocyte sedimentation rate (ESR), haemoglobin, platelet count, white blood cell count, total protein levels, and immunoglobulin G levels.

However, due to the characteristics of sJIA, this trial did not show a significant difference between MTX and placebo in joint scores or systemic features, nor did it lead to a reduction in steroid doses, suggesting that its utility in sJIA may be limited.^24^

Based on the available evidence, if bDMARDs are not available, for patients with predominantly active synovitis, a combination of MTX (10–20 mg/m², up to a maximum dose of 25 mg/week) and oral prednisone or its equivalent at an initial dose of 0.5–1 mg/kg/day (maximum 60 mg/day) is recommended. Ideally, until the third month, steroids dose should be tapered off gradually to a dose between 0.1 and 0.2 mg/kg/day, and discontinued by the sixth month^3^ as shown in **Figure 3** (algorithm for sJIA pharmacological treatment for patients with predominantly active synovitis).

***Recommendation 4:***

For patients with sJIA characterized by predominantly active synovitis and an inadequate response to non-biologic DMARDs, initiate treatment with a biologic DMARD targeting the IL-6 receptor, such as tocilizumab.

Targeting interleukin-6 (IL-6) receptors with tocilizumab is an effective therapeutic option for the treatment of sJIA with polyarticular involvement.^[[26]](#endnote-26)^ Previous studies have demonstrated the effectiveness of tocilizumab in improving joint involvement and radiographic outcomes in children with sJIA, particularly those with polyarticular-course disease.^17^

Medical literature also suggests that tocilizumab may improve radiographic joint damage in children with sJIA. An observational study demonstrated radiographic improvement in large, damaged joints of children treated with tocilizumab.^[[27]](#endnote-27)^

Additionally, a post hoc analysis of two randomised controlled trials indicated that tocilizumab led to significant improvements in growth and joint remodelling. It may also offer benefits in reducing radiographic progression in sJIA, as well as in polyarticular-course JIA.^[[28]](#endnote-28)^

Moreover, the Phase III study conducted by De Benedetti et al. provided significant evidence that treatment with tocilizumab in patients with sJIA can lead to notable catch-up growth and improvements in disease activity. During treatment, patients experienced growth velocities exceeding normal rates, suggesting a substantial recovery in growth.^[[29]](#endnote-29)^

As experts, we acknowledge the lack of comparative studies supporting the use of IL-6 receptor–targeted therapy as a first-line treatment. Nevertheless, the decision to recommend targeting the IL-6 receptor as part of the treatment for sJIA, particularly in patients with polyarticular involvement, is primarily based on expert opinion. This recommendation is driven by promising observational data and the general consensus on the potential benefits of IL-6 receptor-targeted therapy in this population.

Additionally, new agents are being developed, studied, and even used for compassionate purposes, including biological products targeting IL-18, IFNγ, or both IL-1β and IL-18 simultaneously, as well as small molecules like Janus kinase (JAK) inhibitors and stem cell transplantation. However, at the time of writing these recommendations, there were no controlled studies or systematic reviews on these drugs.^[[30]](#endnote-30),^^[[31]](#endnote-31),^^[[32]](#endnote-32),^^[[33]](#endnote-33),^^[[34]](#endnote-34),^^[[35]](#endnote-35),^^[[36]](#endnote-36),^^[[37]](#endnote-37),^^[[38]](#endnote-38)^

**References**

1. Picco P, Gattorno M, Buoncompagni A, Pistoia V, Borrone C. 6-methylprednisolone 'mini-pulses': a new modality of glucocorticoid treatment in systemic onset juvenile chronic arthritis. Scand J Rheumatol. 1996; 25(1):24-7. [↑](#endnote-ref-1)
2. Ravelli A, Lattanzi B, Consolaro A, Martini A. Glucocorticoids in paediatric rheumatology. Clin Exp Rheumatol. 2011;29(5 Suppl 68):S148-S152. [↑](#endnote-ref-2)
3. Fautrel B, Mitrovic S, De Matteis A, Bindoli S, Antón J, Belot A, *et al*. EULAR/PReS recommendations for the diagnosis and management of Still's disease, comprising systemic juvenile idiopathic arthritis and adult-onset Still's disease. Ann Rheum Dis. 2024; 83(12):1614-27. doi: 10.1136/ard-2024-225851. [↑](#endnote-ref-3)
4. Ruperto N, Quartier P, Wulffraat N, et al. A phase II, multicenter, open-label study evaluating dosing and preliminary safety and efficacy of canakinumab in systemic juvenile idiopathic arthritis with active systemic features. Arthritis Rheum. 2012;64 (2):557-67. [↑](#endnote-ref-4)
5. Ruperto N, Brunner HI, Quartier P, Constantin T, Wulffraat N, Horneff G, *et al*. Two randomized trials of canakinumab in systemic juvenile idiopathic arthritis. N Engl J Med. 2012; 367(25):2396-406. [↑](#endnote-ref-5)
6. Ruperto N, Brunner HI, Quartier P, Constantin T, Wulffraat NM, Horneff G, *et al*; Canakinumab in patients with systemic juvenile idiopathic arthritis and active systemic features: results from the 5-year long-term extension of the phase III pivotal trials. Ann Rheum Dis. 2018;77(12):1710-9. [↑](#endnote-ref-6)
7. Quartier P, Allantaz F, Cimaz R, Pillet P, Messiaen C, Bardin C, *et al*. A multicentre, randomised, double-blind, placebo-controlled trial with the interleukin-1 receptor antagonist anakinra in patients with systemic onset juvenile idiopathic arthritis (ANAJIS trial). Ann Rheum Dis. 2011; 70(5):747-54. [↑](#endnote-ref-7)
8. Nigrovic PA. Review: is there a window of opportunity for treatment of systemic juvenile idiopathic arthritis? Arthritis Rheumatol. 2014;66(6):1405-13. [↑](#endnote-ref-8)
9. Nigrovic PA, Mannion M, Prince FH, Zeft A, Rabinovich CE, van Rossum MA, *et al*. Anakinra as first-line disease-modifying therapy in systemic juvenile idiopathic arthritis: report of forty-six patients from an international multicenter series. Arthritis Rheum. 2011;63(2):545-55. doi: 10.1002/art.30128. [↑](#endnote-ref-9)
10. Grom AA, [Ilowite](https://pubmed.ncbi.nlm.nih.gov/?size=200&term=Ilowite+NT&cauthor_id=26314396) NT, [Pascual](https://pubmed.ncbi.nlm.nih.gov/?size=200&term=Pascual+V&cauthor_id=26314396) V, [Brunner](https://pubmed.ncbi.nlm.nih.gov/?size=200&term=Brunner+HI&cauthor_id=26314396) HI, [Martini](https://pubmed.ncbi.nlm.nih.gov/?size=200&term=Martini+A&cauthor_id=26314396) A, [Lovell](https://pubmed.ncbi.nlm.nih.gov/?size=200&term=Lovell+D&cauthor_id=26314396) D, *et al*. Rate and Clinical Presentation of Macrophage Activation Syndrome in Patients With Systemic Juvenile Idiopathic Arthritis Treated With Canakinumab. Arthritis Rheumatol. 2016;68(1):218-28. [↑](#endnote-ref-10)
11. Ilowite NT, Prather K, Lokhnygina Y, Schanberg LE, Elder M, Milojevic D, *et al*. Randomized, double-blind, placebo-controlled trial of the efficacy and safety of rilonacept in the treatment of systemic juvenile idiopathic arthritis. Arthritis Rheumatol. 2014; 66(9):2570-9. [↑](#endnote-ref-11)
12. Lovell DJ, Giannini EH, Reiff AO, Kimura Y, Li S, Hashkes PJ, *et al*. Long-term safety and efficacy of rilonacept in patients with systemic juvenile idiopathic arthritis. Arthritis Rheum. 2013; 65(9):2486-96. [↑](#endnote-ref-12)
13. Scott C, Chan M, Slamang W, Okong'o L, Petty R, Laxer RM, *et al*. Juvenile arthritis management in less resourced countries (JAMLess): consensus recommendations from the Cradle of Humankind. Clin Rheumatol. 2019;38(2):563-575. [↑](#endnote-ref-13)
14. De Benedetti F, Brunner HI, Ruperto N, Kenwright A, Wright S, Calvo I, *et al.* Randomized trial of tocilizumab in systemic juvenile idiopathic arthritis. N Engl J Med. 2012; 367(25):2385-95. [↑](#endnote-ref-14)
15. Yokota S, Imagawa T, Mori M, Miyamae T, Aihara Y, Takei S, *et al*. Efficacy and safety of tocilizumab in patients with systemic-onset juvenile idiopathic arthritis: a randomised, double-blind, placebo-controlled, withdrawal phase III trial. Lancet; 371(9617):998-1006. [↑](#endnote-ref-15)
16. Bielak M, Husmann E, Weyandt N, Haas JP, Hügle B, Horneff G, *et al*. IL-6 blockade in systemic juvenile idiopathic arthritis - achievement of inactive disease and remission (data from the German AID-registry). Pediatr Rheumatol Online J. 2018;16(1):22. [↑](#endnote-ref-16)
17. Yokota S, Imagawa T, Mori M, Miyamae T, Takei S, Iwata N, *et al*. Long term safety and effectiveness of the anti-interleukin 6 receptor monoclonal antibody tocilizumab in patients with systemic juvenile idiopathic arthritis in Japan. J Rheumatol. 2014; 41(4):759-67. [↑](#endnote-ref-17)
18. Nada DW, Moghazy A, Allam AE, Alunno A, Ibrahim AM. Short-Term Outcomes and Predictors of Effectiveness of Tocilizumab in Systemic Juvenile Idiopathic Arthritis: A Prospective Cohort Study. Front Med (Lausanne). 2021;8:665028. [↑](#endnote-ref-18)
19. Kostik MM, Dubko MF, Masalova VV, Snegireva LS, Kornishina TL, Chikova IA, *et al*. Successful treatment with tocilizumab every 4 weeks of a low disease activity group who achieve a drug-free remission in patients with systemic-onset juvenile idiopathic arthritis. Pediatr Rheumatol Online J. 2015; 13:4. [↑](#endnote-ref-19)
20. Ruperto N, Brunner HI, Ramanan AV, Horneff G, Cuttica R, Henrickson M, *et al*. Subcutaneous dosing regimens of tocilizumab in children with systemic or polyarticular juvenile idiopathic arthritis. Rheumatology (Oxford) 2021; 60(10):4568-4580. [↑](#endnote-ref-20)
21. Pal P, Giri PP, Sinha R. Cyclosporine in resistant systemic arthritis-a cheaper alternative to biologics. Indian J Pediatr. 2019;86:590–4. [↑](#endnote-ref-21)
22. Bagri NK. Cyclosporine for Systemic Onset Juvenile Idiopathic Arthritis: Current Stand and Future Directions. Indian J Pediatr. 2019;86(7):576-577. [↑](#endnote-ref-22)
23. Gerloni V, Cimaz R, Gattinara M, Arnoldi C, Pontikaki I, Fantini F. Efficacy and safety profile of cyclosporin A in the treatment of juvenile chronic (idiopathic) arthritis. Results of a 10-year prospective study. Rheumatology (Oxford). 2001; 40(8):907-13. [↑](#endnote-ref-23)
24. Woo P, Southwood TR, Prieur AM, Doré CJ, Grainger J, David J, *et al*. Randomized, placebo-controlled, crossover trial of low-dose oral methotrexate in children with extended oligoarticular or systemic arthritis. Arthritis Rheum. 2000;43(8):1849-57. [↑](#endnote-ref-24)
25. Beukelman T, [Tomlinson](https://pubmed.ncbi.nlm.nih.gov/?size=200&term=Tomlinson+G&cauthor_id=36482434) G, [Nigrovic](https://pubmed.ncbi.nlm.nih.gov/?size=200&term=Nigrovic+PA&cauthor_id=36482434) PA, [Dennos](https://pubmed.ncbi.nlm.nih.gov/?size=200&term=Dennos+A&cauthor_id=36482434) A, [Del Gaizo](https://pubmed.ncbi.nlm.nih.gov/?size=200&term=Del+Gaizo+V&cauthor_id=36482434) V, [Jelinek](https://pubmed.ncbi.nlm.nih.gov/?size=200&term=Jelinek+M&cauthor_id=36482434) M, *et al*. First-line options for systemic juvenile idiopathic arthritis treatment: an observational study of Childhood Arthritis and Rheumatology Research Alliance Consensus Treatment Plans. Pediatr Rheumatol Online J. 2022 Dec 8;20(1):113. [↑](#endnote-ref-25)
26. Turnier JL, Brunner HI. Tocilizumab for treating juvenile idiopathic arthritis. Expert Opin Biol Ther. 2016;16(4):559-66. [↑](#endnote-ref-26)
27. Inaba Y, Ozawa R, Imagawa T, Mori M, Hara Y, Miyamae T, Aoki C, Saito T, Yokota S. Radiographic improvement of damaged large joints in children with systemic juvenile idiopathic arthritis following tocilizumab treatment. Ann Rheum Dis. 2011 Sep;70(9):1693-5. doi: 10.1136/ard.2010.145359. Epub 2011 Mar 14. PMID: 21402562. [↑](#endnote-ref-27)
28. Malattia C, Ruperto N, Pederzoli S, Palmisani E, Pistorio A, Wouters C, Dolezalova P, Flato B, Garay S, Giancane G, Wells C, Douglass W, Brunner HI, De Benedetti F, Ravelli A; Paediatric Rheumatology International Trials Organisation (PRINTO) and the Pediatric Rheumatology Collaborative Study Group (PRCSG). Tocilizumab may slow radiographic progression in patients with systemic or polyarticular-course juvenile idiopathic arthritis: post hoc radiographic analysis from two randomized controlled trials. Arthritis Res Ther. 2020 Sep 10;22(1):211. doi: 10.1186/s13075-020-02303-y. PMID: 32912276; PMCID: PMC7488325. [↑](#endnote-ref-28)
29. De Benedetti F, Brunner H, Ruperto N, Schneider R, Xavier R, Allen R, Brown DE, Chaitow J, Pardeo M, Espada G, Gerloni V, Myones BL, Frane JW, Wang J, Lipman TH, Bharucha KN, Martini A, Lovell D; Paediatric Rheumatology International Trials Organisation and the Pediatric Rheumatology Collaborative Study Group. Catch-up growth during tocilizumab therapy for systemic juvenile idiopathic arthritis: results from a phase III trial. Arthritis Rheumatol. 2015 Mar;67(3):840-8. doi: 10.1002/art.38984. PMID: 25504861. [↑](#endnote-ref-29)
30. Coto C, Varela G, Hernández V, del Rosario M, López-Saura P. Use of recombinant interferon gamma in pediatric patients with advanced juvenile chronic arthritis. Biotherapy. 1998; 11(1):15-20. [↑](#endnote-ref-30)
31. [Verweyen](about:blank) EL, Schulert GS. [Interfering with interferons: targeting the JAK-STAT pathway in complications of systemic juvenile idiopathic arthritis (SJIA).](about:blank) Rheumatology (Oxford). 2023;62(5):2022. [↑](#endnote-ref-31)
32. Brinkman DM, de Kleer IM, ten Cate R, van Rossum MA, Bekkering WP, Fasth A, *et al*. Autologous stem cell transplantation in children with severe progressive systemic or polyarticular juvenile idiopathic arthritis: long-term follow-up of a prospective clinical trial. Arthritis Rheum. 2007; 56(7):2410-21. [↑](#endnote-ref-32)
33. Silva J, Ladomenou F, Carpenter B, Chandra S, Sedlacek P, Formankova R, *et al*. Allogeneic hematopoietic stem cell transplantation for severe, refractory juvenile idiopathic arthritis. Blood Adv. 2018; 2(7):777-786. [↑](#endnote-ref-33)
34. Swart JF, de Roock S, Nievelstein RAJ, Slaper-Cortenbach ICM, Boelens JJ, Wulffraat NM. Bone-marrow derived mesenchymal stromal cells infusion in therapy refractory juvenile idiopathic arthritis patients. Rheumatology (Oxford). 2019; 58(10):1812-1817. [↑](#endnote-ref-34)
35. De Benedetti F, Grom AA, Brogan PA, Bracaglia C, Pardeo M, Marucci G, *et al*. Efficacy and safety of emapalumab in macrophage activation syndrome. Ann Rheum Dis. 2023;82(6):857-865. [↑](#endnote-ref-35)
36. Gabay C, Fautrel B, Rech J, Spertini F, Feist E, Kötter I, *et al*. Open-label, multicentre, dose-escalating phase II clinical trial on the safety and efficacy of tadekinig alfa (IL-18BP) in adult-onset Still's disease. Ann Rheum Dis. 2018;77(6):840-847. [↑](#endnote-ref-36)
37. Melki I, Frémond M-L. JAK Inhibition in Juvenile Idiopathic Arthritis (JIA): Better Understanding of a Promising Therapy for Refractory Cases. J Clin Med. 2023;12(14):4695. [↑](#endnote-ref-37)
38. Onel K, Rumsey DG, Shenoi S. Juvenile Idiopathic Arthritis Treatment Updates. Rheum Dis Clin North Am. 2021;47(4):545-563. [↑](#endnote-ref-38)
