# Supplementary material for: Pan American League of Associations for Rheumatology treatment recommendations for systemic juvenile idiopathic arthritis
Source: Rheumatol Adv Pract. 2025 Nov 11;9(4):rkaf087. doi: 10.1093/rap/rkaf087 (PMC12607261; doi:10.1093/rap/rkaf087)
Supplement: rkaf087_Supplementary_Data [file rkaf087_supplementary_data.zip › Supplementary_Data_1._Systemic_JIA_Team_Panel_19_03_2025_clean_version (1).docx]

**SUPPLEMENTARY DATA S1:**

**Part 1:** Team Panel and Disclosures

**Part 2:** Methodology

**Part 3:** Scope and PICO Development

**Part 4:** Literature Source and Search

**Part 5:** Study Selection, flowchart, included and excluded articles.

**SUPPLEMENTARY DATA S2:** Summary of evidence

**SUPPLEMENTARY DATA S3:** GRADE profile

**SUPPLEMENTARY DATA S4:** Recommendations with cites.

**SUPPLEMENTARY DATA S5:** Educative appendix

**SUPPLEMENTARY DATA 1 - PART 1: Team Panel and Disclosures**

All the authors are in representation of the Pan American League of Associations for Rheumatology (PANLAR).

**Methodologist Group**

| *Leader group:* | *Members:* |
| --- | --- |
| Brance, María Lorena | Brun, Lucas R.  Marín Zúcaro, Nicolás M.  Ramirez Stieben, Luis A.  Ringer, Ariana |

**PANLAR Research Unit**

Fernández-Ávila, Daniel G & Brance, María Lorena

| **PICO JIA Panel Experts** | **Internal Revisor JIA Expert Panel** |
| --- | --- |
| Barzola, María L.  Cameto Caffa, Juan A.  Faugier Fuentes, Enrique  Franco, Lorena  Guarnizo Zuccardi, Pilar  Herrera, Cristina N.  Ibañez Estrella, Amparo  Jiménez Cruz, Karen V.  Jurado, Rosario M.  León, Beatriz H.  Paz Gastañaga, María E.  Rodrigues Fonseca, Adriana  Rodriguez Tineo, Carmen  Rubio Pérez, Nadina E.  Stichweh, Dorothee S  Talesnik, Eduardo | Appenzeller, Simone  Arroyo Rivera, Ivonne L.  Cuttica, Rubén J.  Eraso, Ruth  Espada, Graciela  Ferrándiz, Manuel A.  Gutierrez-Suárez, Raúl  Morel, Zoilo  Silva, Clovis A. |

**Voting JIA Expert Panel** (Both panel, expert and PICO)

**Author’s affiliations**

| **Author** | **Degree** | **University/Hospital** | **Country** |
| --- | --- | --- | --- |
| Appenzeller, Simone  [appenzellersimone@gmail.com](mailto:appenzellersimone@gmail.com) ORCID: 0000-0001-5075-4474 | MD, PhD | University of Campinas | Brazil |
| Arroyo Rivera, Ivonne L. i_arroyo2003@yahoo.com  ORCID: 0009-0001-0688-9707 | MD | San Juan City Hospital | Puerto Rico |
| Barzola, Maria L.  [marialaurabarzola@yahoo.com.ar](mailto:marialaurabarzola@yahoo.com.ar) | MD | Hospital de Niños Ricardo Gutiérrez. Universidad de Buenos Aires | Argentina |
| Brance, María Lorena  [lorenabrance@gmail.com](mailto:lorenabrance@gmail.com)  ORCID: 0000-0002-6749-9921 | MD, PhD | Rosario National University | Argentina |
| Brun, Lucas R.  [lbrun@unr.edu.ar](mailto:lbrun@unr.edu.ar)  ORCID: 0000-0001-6281-2096 | MD, PhD | Rosario National University | Argentina |
| Cameto Caffa, Juan A.  [cametojuan@gmail.com](mailto:cametojuan@gmail.com)  ORCID: 0000-0003-4119-9207 | MD | Centro Hospitalario Pereira Rossell | Uruguay |
| Cuttica, Rubén J.  [cutticarj@yahoo.com.ar](mailto:cutticarj@yahoo.com.ar) | MD | Hospital General de Niños Pedro de Elizalde. | Argentina |
| Eraso, Ruth  [rutheraso@gmail.com](mailto:rutheraso@gmail.com) | MD | Hospital Pablo Tobón Uribe / Universidad de Antioquia | Colombia |
| Espada, Graciela  [graespada@gmail.com](mailto:graespada@gmail.com)  ORCID: 0000-0002-4897-968X | MD | Hospital de Niños Dr. Ricardo Gutierrez, Buenos Aires | Argentina |
| Faugier Fuentes, Enrique  [dr@efaugier.com](mailto:dr@efaugier.com)   ORCID: 0000-0001-7807-1296 | MD | Hospital Infantil de México Federico Gomez | México |
| Fernández-Ávila, Daniel G.  [daniel.fernandez@javeriana.edu.co](mailto:daniel.fernandez@javeriana.edu.co) | MD. PhD | Pontificia Universidad Javeriana | Colombia |
| Ferrándiz, Manuel A.  [manuelferrandiz@hotmail.com](mailto:manuelferrandiz@hotmail.com)  ORCID: 0000-0001-6101-8608 | MD | Instituto Nacional de Salud del Niño (INSN), Lima, Perú | Perú |
| Franco, Lorena  [franco.lore@gmail.com](mailto:franco.lore@gmail.com)  ORCID: 0000-0003-3820-2305 | MD | Hospital Infantil Municipal Córdoba. Hospital Privado Universitario | Argentina |
| Guarnizo Zuccardi, Pilar  [pilarguarnizo@gmail.com](mailto:pilarguarnizo@gmail.com) | MD | Fundación cardioinfantil de Bogotá | Colombia |
| Gutierrez-Suárez, Raúl  [raul.gutierrez.suarez.reep@gmail.com](mailto:raul.gutierrez.suarez.reep@gmail.com)  ORCID: 0000-0001-7326-6668 | MD. MSc. | Hospital del niño. Instituto Materno Infantil del Estado de México. (IMIEM). | México |
| Herrera, Cristina N.  [cristinaherrera227@gmail.com](mailto:cristinaherrera227@gmail.com) ORCID: 0000-0001-6768-7618 | MD | Hospital Roberto Gilbert  Elizalde, Guayaquil. | Ecuador |
| Ibáñez Estrella, Amparo  [amparoies2003@gmail.com](mailto:amparoies2003@gmail.com)  ORCID: 0000-0002-8921-929X | MD | Instituto Nacional de Salud del Niño Breña | Peru |
| Jiménez Cruz, Karen V.  [kjimenezcruz@gmail.com](mailto:kjimenezcruz@gmail.com)  ORCID: 0000-0001-6690-7340 | MD | Universidad El Bosque, Clínica Pediátrica, Clínica Colsanitas | Colombia |
| Jurado, Rosario M.  [rosariomjurado@gmail.com](mailto:rosariomjurado@gmail.com) | MD | Centro Hospitalario Pereira Rossell. | Uruguay |
| León, Beatriz H.  [docbealeon@yahoo.com](mailto:docbealeon@yahoo.com) | MD | Universidad San Francisco de Quito | Ecuador |
| Marín Zúcaro, Nicolas M.  [nicolas.marin@hospitalitaliano.org.ar](mailto:nicolas.marin@hospitalitaliano.org.ar)  ORCID: 0000-0002-2957-3617 | MD | Italian Hospital, Buenos Aires. | Argentina |
| Morel, Zoilo  [zoiloma@hotmail.com](mailto:zoiloma@hotmail.com)  ORCID: 0000-0001-6438-446X | MD. MSc | Universidad Nacional de Asunción. | Paraguay |
| Ramirez Stieben, Luis A.  [ramirez.stieben@gmail.com](mailto:ramirez.stieben@gmail.com)  ORCID: 0000-0002-8212-1257 | MD. PhD. | Rosario National University | Argentina |
| Ringer, Ariana  [aruris15151@gmail.com](mailto:aruris15151@gmail.com) ORCID: 0000-0002-2610-5818 | MD | Rosario National University | Argentina |
| Rodrigues Fonseca, Adriana  [adrirfonseca@gmail.com](mailto:adrirfonseca@gmail.com)  ORCID: 0000-0002-3536-3552 | MD. PhD. | Universidade Federal do Rio de Janeiro | Brazil |
| Rodriguez Tineo, Carmen  [dra.tineo@hotmail.com](mailto:dra.tineo@hotmail.com) | MD | Hospital Cabral y Báez/Hospital Infantil Arturo Grullón | Dominican Republic |
| Silva, Clovis Artur  [clovisaasilva@gmail.com](mailto:clovisaasilva@gmail.com)  ORCID: 0000-0001-9250-6508 | MD | Hospital das Clínicas da Faculdade de Medicina da Universidade de São Paulo | Brazil |
| Talesnik, Eduardo  [eduardotalesnik@gmail.com](mailto:eduardotalesnik@gmail.com) | MD | Pontificia Universidad Católica de Chile | Chile |

**Author’s disclosures within the last 12 months as per PANLAR policy**

| **Author** | **Disclosures** |
| --- | --- |
| Appenzeller, Simone | Research Grant: From the National Council for Scientific and Technological Development 305981/2023-4 |
| Arroyo Rivera, Ivonne L. | None |
| Barzola, María L. | None |
| Brance, María Lorena | None |
| Brun, Lucas R. | None |
| Cameto Caffa, Juan A. | None |
| Cuttica, Rubén J. | None |
| Eraso, Ruth | Speaker, consulting, or research support: Novartis, Abbvie, Pfizer, Roche |
| Espada, Graciela | Speaker, consulting, or research support: Novartis, Abbvie |
| Faugier Fuentes, Enrique | None |
| Fernández-Ávila, Daniel G. | None |
| Ferrándiz, Manuel A. | None |
| Franco, Lorena | None |
| Guarnizo Zuccardi, Pilar | Speaker, consulting, or research support: Novartis, Alexion |
| Gutierrez-Suárez, Raúl | None |
| Herrera, Cristina N. | Speaker, consulting, or research support: Roche |
| Ibañez Estrella, Amparo | None |
| Jiménez Cruz, Karen V. | Speaker, consulting, or research support: Abbvie, Pfizer, Alexion |
| Jurado, Rosario M. | None |
| León, Beatriz H | None |
| Marín Zúcaro, Nicolas M. | None |
| Morel, Zoilo | None |
| Ramirez Stieben, Luis A. | None |
| Ringer, Ariana | None |
| Rodrigues Fonseca, Adriana | Research grant: Fundação Carlos Chagas Filho de Amparo à Pesquisa do Estado do Rio de Janeiro (FAPERJ) 260003/006024/2023. |
| Rodriguez Tineo, Carmen | Speaker, consulting, or research support: Novartis, Janssen, AstraZeneca |
| Silva, Clovis A. | Fundação de Amparo à Pesquisa do Estado de São Paulo (FAPESP) (#2015/03756-4 to CAS), Conselho Nacional de Desenvolvimento Científico e Tecnológico (CNPq 304984/2020-5 to CAS), and by Núcleo de Apoio à Pesquisa “Saúde da Criança e do Adolescente” da USP (NAP-CriAd) |
| Talesnik, Eduardo | None |

**SUPPLEMENTARY DATA 1 - PART 2: Methodology**

These guidelines have been sponsored and endorsed by the Pan American League of Associations for Rheumatology (PANLAR), and were developed using the Grading of Recommendations Assessment, Development and Evaluation (GRADE) ^[[1]](#endnote-2)^ approach ([www.gradeworkinggroup.org](http://www.gradeworkinggroup.org)) in a process led by a GRADE Methodologist Group.

The guidelines’ working group was divided into two teams*:* A GRADE Methodologist group and a group of experts in JIA. The GRADE methodologist group supervised every stage of the guideline’s development. The GRADE Methodologist Group supported the JIA experts in the process of prioritization of the clinical questions and outcomes, and the development of clinical questions in a Population/Intervention/Comparator/Outcome (PICO) format.^[[2]](#endnote-3)^ In addition, the GRADE Methodologist Group completed the literature searches, studies selection, data extraction and analysis. The group of experts in JIA defined the scope of these guidelines, elaborated the PICO questions, and voted the PICO questions and recommendations based on the evidence report and their expertise, which included making judgments regarding the relative importance of the outcomes. In order to be part of these guidelines, a recommendation required at least 70% of agreement among the voting JIA experts. The guidelines were reviewed by two external reviewers with expertise and leadership in the field of JIA.

The different processes were carried out as detailed below:

***Scope and PICO Development***. The scope of these guidelines was defined by the JIA experts. The PICO questions were elaborated by the JIA experts.

The ***population*** studied was patients who were diagnosed with JIA.

The ***interventions*** were different pharmacologic treatments (glucocorticoids, methotrexate, cDMARDs, etanercept, abatacept, rituximab, anakinra, tocilizumab, canakinumab, interferon gamma, rilonacept).

The ***comparators*** were other pharmacologic treatments and placebo, according to each PICO question.

The ***outcomes*** considered critically important for each intervention were those related with safety (e.g., side effects) and efficacy.

**Searches of the literature.** A systematic literature search for published randomized controlled trials, nonrandomized trials, cohort studies, post hoc analysis and pooled analysis was conducted up to February 2022.

***Literature Source and Search***

MEDLINE/PubMed (<https://pubmed.ncbi.nlm.nih.gov/>), the Cochrane Library (<https://www.cochranelibrary.com/>) including Cochrane Database of Systematic Reviews and EMBASE (<https://www.elsevier.com/products/embase/>) were systematically searched for published randomized controlled trials, non-randomized trials, cohort studies, post hoc analysis and pooled analysis from inception of each database to February 2022. The search was carried out combining MeSH Terms and Entry Terms combined with booleans (OR, AND, not) in order to capture the greatest number of studies. The grey literature and the abstracts of the last five European Alliance of Associations for Rheumatology (EULAR) and American College of Rheumatology (ACR) annual meetings were evaluated.

***Study Selection***

Rayyan software (<https://rayyan-prod.qcri.org/>) was used to screen the literature search results. We performed duplicate screening of each title and abstract using two independent reviewers with a third reviewer resolving potential conflicts. Eligible articles underwent full-text screening by two independent reviewers. Selected manuscripts were matched to PICO questions. All the studies (included and excluded) and the flowchart are in *Supplementary Data 1.*

***Data Extraction and Analysis***

Pooling of the data for statistical analysis was done using Review Manager (RevMan) software V.5.4.1 (Copenhagen: The Nordic Cochrane Centre, The Cochrane Collaboration) for statistical analysis of the data. The quality of each randomized clinical trial was assessed using the Cochrane risk of bias tool (<http://handbook.cochrane.org/>) while the data from observational cohort studies have been analyzed using Newcastle-Ottawa Scale. Binary outcomes (e.g., disease relapse) were analyzed using the Mantel-Haenszel method in a random effects model and reported as relative risks with 95% confidence intervals (CI). Continuous outcomes (e.g., cumulative glucocorticoid dose) were analyzed using the inverse variance as statistical method in a random effects model and reported as 95% CI and standard deviations (SD).

***Evidence Report Formulation***

We exported RevMan files into GRADEpro GDT software (<https://gradepro.org/>) to formulate tables containing the GRADE profile of each PICO question.^[[3]](#endnote-4)^ The summary of evidence is in *Supplementary Data 2*. The quality of evidence for each outcome was evaluated by two independent reviewers using GRADE quality assessment criteria. ^[[4]](#endnote-5),^ ^[[5]](#endnote-6),^ ^[[6]](#endnote-7),^ ^[[7]](#endnote-8),^ ^[[8]](#endnote-9),^ ^[[9]](#endnote-10)^ Potential discordances were resolved through discussion with a third reviewer. The GRADE profile is in *Supplementary Data 3.* The GRADE methodology group reviewed the evidence report and addressed possible evidence gaps prior to presenting the data to the RA experts for voting. The GRADE methodology group also elaborated a concise evidence summary of the evidence for each PICO question.

***Consensus***

The group of RA experts had ample time to review the evidence report and the concise evidence summary of each PICO question before voting. The voting process was anonymous and completed via Zoom software^[[10]](#endnote-11)^ platform. If the voting of a particular PICO question did not reach a consensus of 70% agreement in the first instance, the members held discussions before voting again. Only three voting were allowed. If there was no not agreement among at least 70% of the voting members after three attempts, the recommendation was considered ungraded.

The GRADE methodology differentiates four levels of evidence quality (i.e., high, moderate, low or very low) based on the degree of confidence that the measured effect reached after the analysis of the pooled? studies is close to the true effect. If a recommendation was made on the basis of consensus of expert opinions in the absence of evidence, then the evidence for that recommendation was graded as very low quality.

***Rigour of development*.** These guidelines have been developed under GRADE methodology and abides by the AGREE Reporting Checklist to ensure the completeness and transparency of reporting in practice guidelines. In keeping with the GRADE methodology, the group of JIA experts developed recommendations for different clinical scenarios. All recommendations required achieving at least 70% level of agreement at the voting stage. Each recommendation was made taking into account the risk to benefit ratio and the quality of the evidence available of each intervention considered. A recommendation could be either in favor or against the proposed intervention and either be qualified as being strong or conditional (i.e., weak). Strong recommendations are those for which the panel was highly confident that the proposed intervention favorably balances the expected benefits and risks for the majority of patients in clinical practice. Conditional recommendations are those for which the panel was less confident that the potential benefits outweigh the risks. A recommendation can be conditional either because of low or very low quality of evidence supporting one option over another, or because there is uncertainty in terms of risk to benefit ratio.^22^

This guideline abides by the AGREE Reporting Checklist to ensure the completeness and transparency of reporting in practice guidelines. ^^[[11]](#endnote-12)^^

**SUPPLEMENTARY DATA 3 -PART 3: PICO Development**

**Juvenile idiopathic arthritis: Oligoarticular (PICO 1-19)**

**Juvenile idiopathic arthritis: Polyarticular (PICO 20-59)**

**Juvenile idiopathic arthritis: Systemic (PICO 60-69)**

**Juvenile idiopathic arthritis: Systemic (PICO 60-69)**

**PICO 60**

P: Patients with Systemic JIA

I: Pulse Methylprednisolone

C: Prednisone (1 mg/kg/day)

O: Efficacy (remission, relapse, functional ability, parents/patients reported outcomes) and safety (adverse events)

**PICO 61**

P: Patients with Systemic JIA

I: Methotrexate

C: Placebo

O: Efficacy (remission, relapse, functional ability, parents/patients reported outcomes) and safety (adverse events)

**PICO 62**

P: Patients with Systemic JIA

I: Anakinra

C: Placebo

O: Efficacy (remission, relapse, functional ability, parents/patients reported outcomes) and safety (adverse events)

**PICO 63**

P: Patients with Systemic JIA

I: Anakinra first line

C: Anakinra second line

O: Efficacy (remission, relapse, functional ability, parents/patients reported outcomes) and safety (adverse events)

**PICO 64**

P: Patients with Systemic JIA

I: Tocilizumab

C: Placebo

O: Efficacy (remission, relapse, functional ability, parents/patients reported outcomes) and safety (adverse events)

**PICO 65**

P: Patients with Systemic JIA

I: Tocilizumab (Q2W)

C: Tocilizumab (Q4W)

O: Efficacy (remission, relapse, functional ability, parents/patients reported outcomes) and safety (adverse events)

**PICO 66**

P: Patients with Systemic JIA

I: Canakinumab

C: Placebo

O: Efficacy (remission, relapse, functional ability, parents/patients reported outcomes) and safety (adverse events)

**PICO 67**

P: Patients with Systemic JIA

I: Canakinumab

C: Tapering

O: Efficacy (remission, relapse, functional ability, parents/patients reported outcomes) and safety (adverse events)

**PICO 68**

P: Patients with Systemic JIA

I: Canakinumab naïve

C: Switching to Canakinumab from Tocilizumab

O: Efficacy (remission, relapse, functional ability, parents/patients reported outcomes) and safety (adverse events)

**PICO 69**

P: Patients with Systemic JIA

I: Rilonacept

C: Placebo

O: Efficacy (remission, relapse, functional ability, parents/patients reported outcomes) and safety (adverse events)

**SUPPLEMENTARY DATA 1 - PART 4: Literature Search**

A literature search was carried out to answer 9 clinical questions formulated in the Population/Intervention/Comparator/Outcome (PICO) format. The PICO questions are listed in Supplementary Data-Part 3. The evidence was searched in the MEDLINE/PubMed (<https://pubmed.ncbi.nlm.nih.gov/>), Cochrane Library (<https://www.cochranelibrary.com/>), and EMBASE (<https://www.elsevier.com/products/embase/>) databases from the inception of each database to February, 2022. The grey literature including abstracts of the last five European Alliance of Associations for Rheumatology (EULAR) and American College of Rheumatology (ACR) annual meetings was also searched.

The search was carried out using MeSH (Medical Subject Headings) Terms and Entry Terms to capture the greatest number of studies. Boolean operators (OR, AND, NOT) were used to combine or exclude Terms resulting in more focused and productive results. MeSH or Entry Terms corresponding to a PICO question was combinated with OR while the link with the next Term was made with AND. To exclude animal studies, NOT was used (i.e., animals [mh] not humans [mh]). Some Terms were truncated at their root to increase the number of articles retrieved. The Terms were searched in the Titles and Abstracts to increase the specificity of the search results.

**Eligibility Criteria.**^2^ Studies with inclusion criteria for these guidelines were published randomized controlled trials, nonrandomized trials, cohort studies, post hoc analysis and pooled analysis. Only articles in English, Portuguese or Spanish were included. Case reports, reviews, letters to editors, animal studies, editorials, commentaries, duplicates, studies re-analyzing identical data previously published, and studies in languages other than English, Portuguese or Spanish were excluded.

**Databases**

**MEDLINE/PubMed**

Web link: <https://pubmed.ncbi.nlm.nih.gov/>

Search strategy: The search strategy was done in three running searches (polyarthritis-oligoarthritis and systemic).

**PubMed. First search up to 02-23-2022. JIA systemic**

((SYSTEMIC ARTHRITIS, JUVENILE[Text Word] OR ARTHRITIS, JUVENILE SYSTEMIC[Text Word] OR JUVENILE SYSTEMIC ARTHRITIS[Text Word] OR JUVENILE-ONSET STILL DISEASE[Text Word] OR JUVENILE ONSET STILL DISEASE[Text Word] OR STILL'S DISEASE, JUVENILE-ONSET[Text Word] OR JUVENILE-ONSET STILL'S DISEASE[Text Word] OR STILL'S DISEASE, JUVENILE ONSET[Text Word] OR STILL DISEASE, JUVENILE-ONSET[Text Word] OR STILL DISEASE, JUVENILE ONSET[Text Word] OR JUVENILE-ONSET STILLS DISEASE[Text Word] OR JUVENILE ONSET STILLS DISEASE[Text Word] OR STILLS DISEASE, JUVENILE-ONSET[Text Word] OR MACROPHAGE ACTIVATION SYNDROME[Text Word])) AND (("GLUCOCORTICOIDS"[Pharmacological Action] OR "GLUCOCORTICOIDS"[MeSH Terms] OR GLUCOCORTICOID*[Text Word] OR CORTICOSTEROID*[Text Word] OR CORTICOID*[Text Word] OR STEROID*[Text Word] OR "PREDNISOLONE"[MeSH Terms] OR PREDNISOLONE[Text Word] OR "PREDNISONE"[MeSH Terms] OR PREDNISONE[Text Word] OR "DEFLAZACORT" [Supplementary Concept] OR DEFLAZACORT[Text Word] OR "METHYLPREDNISOLONE"[MeSH Terms] OR METHYLPREDNISOLONE[Text Word] OR "ANTIRHEUMATIC AGENTS"[Pharmacological Action] OR "ANTIRHEUMATIC AGENTS"[MeSH Terms] OR ANTIRHEUMATIC AGENT*[TW] OR ANTI-RHEUMATIC AGENT*[TW] OR ANTI RHEUMATIC AGENT*[TW] OR ANTIRHEUMATIC DRUG*[TW] OR ANTI-RHEUMATIC DRUG*[TW] OR ANTI RHEUMATIC DRUG*[TW] OR DMARD*[TW] OR DISEASE-MODIFYING ANTIRHEUMATIC DRUG*[TW] OR DISEASE MODIFYING ANTIRHEUMATIC DRUG*[TW] OR ANTIRHEUMATIC DISEASE-MODIFYING SECOND-LINE DRUG*[TW] OR ANTIRHEUMATIC DISEASE MODIFYING SECOND LINE DRUG*[TW] OR NON-STEROIDAL ANTI-RHEUMATIC AGENT*[Text Word] OR NONSTEROIDAL ANTI-RHEUMATIC AGENT*[Text Word] OR NON STEROIDAL ANTI-RHEUMATIC AGENT*[Text Word] OR NON-STEROIDAL ANTIRHEUMATIC AGENT*[Text Word] OR NONSTEROIDAL ANTIRHEUMATIC AGENT*[Text Word] OR NON STEROIDAL ANTIRHEUMATIC AGENT*[Text Word] OR NON-STEROIDAL ANTI RHEUMATIC AGENT*[Text Word] OR NONSTEROIDAL ANTI RHEUMATIC AGENT*[Text Word] OR NON STEROIDAL ANTI RHEUMATIC AGENT*[Text Word] OR "METHOTREXATE"[MeSH Terms] OR METHOTREXATE[Text Word] OR "CYCLOSPORINE"[MeSH Terms] OR CYCLOSPORINE[Text Word] OR CYCLOSPORIN[Text Word] OR CICLOSPORIN[Text Word] OR OL 27-400[Text Word] OR OL 27 400 [Text Word] OR OL 27400[Text Word] OR Tumor Necrosis Factor Inhibitors [Pharmacological Action] OR TNFi*[Text Word] OR TNF ALPHA INHIBITOR*[Text Word] OR TNF-ALPHA INHIBITOR*[Text Word] OR TNF INHIBITOR*[Text Word] OR TNF-INHIBITOR*[Text Word] OR TUMOUR NECROSIS FACTOR ALPHA INHIBITOR*[Text Word] OR TUMOUR NECROSIS FACTOR INHIBITOR*[Text Word] OR TUMOR NECROSIS FACTOR ALPHA INHIBITOR*[Text Word] OR TUMOR NECROSIS FACTOR INHIBITOR*[Text Word] OR ANTI TUMOR NECROSIS FACTOR AGENT*[Text Word] OR ANTI-TUMOR NECROSIS FACTOR AGENT*[Text Word] OR ANTI TUMOUR NECROSIS FACTOR AGENT*[Text Word] OR ANTI-TUMOUR NECROSIS FACTOR AGENT*[Text Word] OR ANTI-TNF ALPHA AGENT*[Text Word] OR ANTI TNF ALPHA AGENT*[Text Word] OR ANTI TNF AGENT*[Text Word] OR ANTI-TNF AGENT*[Text Word] OR "ADALIMUMAB"[MeSH Terms] OR ADALIMUMAB[Text Word] OR MONOCLONAL ANTIBODY D2E7[TW] OR D2E7 ANTIBODY[TW] OR "ETANERCEPT"[MeSH Terms] OR ETANERCEPT[Text Word] OR TNFR-FC FUSION PROTEIN[Text Word] OR TNFR FC FUSION PROTEIN[Text Word] OR TNF RECEPTOR TYPE II-IGG FUSION PROTEIN[Text Word] OR TNF RECEPTOR TYPE II IGG FUSION PROTEIN[Text Word] OR RECOMBINANT HUMAN DIMERIC TNF RECEPTOR TYPE II-IGG FUSION PROTEIN[Text Word] OR RECOMBINANT HUMAN DIMERIC TNF RECEPTOR TYPE II IGG FUSION PROTEIN [Text Word] OR TNT RECEPTOR FUSION PROTEIN[Text Word] OR TNR-001[Text Word] OR TNR 001[Text Word] OR TNR001[Text Word] OR "INFLIXIMAB"[MeSH Terms] OR INFLIXIMAB[Text Word] OR MAB CA2[Text Word] OR MONOCLONAL ANTIBODY CA2[Text Word] OR "GOLIMUMAB"[ Supplementary Concept] OR GOLIMUMAB[Text Word] OR CNTO 148[Text Word] OR CNTO148[Text Word] OR CNTO-148[Text Word] OR TsDMARD*[TW] OR TARGETED SYNTHETIC DMARD*[TW] OR TARGETED SYNTHETIC DISEASE-MODIFYING ANTIRHEUMATIC DRUG*[TW] OR TARGETED SYNTHETIC DISEASE MODIFYING ANTIRHEUMATIC DRUG*[TW] OR TOFACITINIB[Supplementary Concept] OR TOFACITINIB[Text Word] OR TASOCITINIB [Text Word] OR CP-690550[Text Word] OR CP-690,550[Text Word] OR CP 690550[Text Word] OR CP 690,550[Text Word] OR CP690550[Text Word] OR CP690,550[Text Word] OR BARICITINIB[Supplementary Concept] OR INCB-28050[Text Word] OR INCB028050[Text Word] OR INCB-028050[Text Word] OR LY3009104[Text Word] OR LY-3009104[Text Word] OR TOCILIZUMAB[Supplementary Concept] OR TOCILIZUMAB[Text Word] OR ATLIZUMAB[Text Word] OR BAT-1806[Text Word] OR BAT1806[Text Word] OR MSB-11456[Text Word] OR MSB11456[Text Word] OR RG-1569[Text Word] OR R-1569[Text Word] OR RHPM-1[Text Word] OR RO-4877533[Text Word] OR SARILUMAB[Supplementary Concept] OR SARILUMAB[Text Word] OR SAR-153191[Text Word] OR SAR153191[Text Word] OR REGN-88[Text Word] OR REGN88[Text Word] OR "RITUXIMAB"[MeSH Terms] OR RITUXIMAB[TW] OR IDEC-C2B8 ANTIBODY[Text Word] OR IDEC C2B8 ANTIBODY[Text Word] OR IDEC-C2B8[Text Word] OR IDEC C2B8[Text Word] OR GP2013[Text Word] OR ANAKINRA[Text Word] OR INTERLEUKIN 1 RECEPTOR ANTAGONIST PROTEIN[MeSH Terms] OR INTERLEUKIN 1 INHIBITOR[Text Word] OR IL-1 Inhibitor[Text Word] OR IL1 Inhibitor[Text Word] OR CANAKINUMAB[Supplementary Concept] OR CANAKINUMAB[Text Word] OR ACZ-885[Text Word] OR ACZ885[Text Word] OR ANTI-HUMAN INTERLEUKIN 1BETA[Text Word] OR RILONACEPT[Supplementary Concept] OR RILONACEPT[Text Word] OR INTERLEUKIN-1 TRAP [Text Word] OR "USTEKINUMAB"[MeSH Terms] OR USTEKINUMAB[TW] OR CNTO 1275[Text Word] OR CNTO-1275[Text Word]) OR CYCLOPHOSPHAMIDE[MeSH Terms] OR B-518[Text Word] OR B 518[Text Word] OR B518[Text Word] OR NSC-26271[Text Word] OR NSC 26271[Text Word] OR NSC26271[Text Word] OR THALIDOMIDE[Text Word] OR GAMMA GLOBULINS[Text Word] OR GAMMA-GLOBULIN[Text Word] OR GAMMA GLOBULIN[Text Word] OR Emapalumab[Supplementary Concept] OR Etoposide[MeSH Terms] OR NSC-141540[Text Word] OR NSC 141540[Text Word] OR NSC141540[Text Word] OR VP 16-213[Text Word] OR VP 16 213[Text Word] OR VP 16213[Text Word] OR VP-16[Text Word] OR VP 16[Text Word] OR VP16[Text Word] OR "BONE MARROW TRANSPLANTATION"[MeSH Terms])

**EMBASE**

Web link: (<https://www.elsevier.com/products/embase/>)

Search strategy: The search strategy was done in three running searches.

**Embase. First search up to 02-23-2022. JIA Systemic**

((SYSTEMIC ARTHRITIS, JUVENILE[Text Word] OR ARTHRITIS, JUVENILE SYSTEMIC[Text Word] OR JUVENILE SYSTEMIC ARTHRITIS[Text Word] OR JUVENILE-ONSET STILL DISEASE[Text Word] OR JUVENILE ONSET STILL DISEASE[Text Word] OR STILL'S DISEASE, JUVENILE-ONSET[Text Word] OR JUVENILE-ONSET STILL'S DISEASE[Text Word] OR STILL'S DISEASE, JUVENILE ONSET[Text Word] OR STILL DISEASE, JUVENILE-ONSET[Text Word] OR STILL DISEASE, JUVENILE ONSET[Text Word] OR JUVENILE-ONSET STILLS DISEASE[Text Word] OR JUVENILE ONSET STILLS DISEASE[Text Word] OR STILLS DISEASE, JUVENILE-ONSET[Text Word] OR MACROPHAGE ACTIVATION SYNDROME[Text Word])) AND (("GLUCOCORTICOIDS"[Pharmacological Action] OR "GLUCOCORTICOIDS"[MeSH Terms] OR GLUCOCORTICOID*[Text Word] OR CORTICOSTEROID*[Text Word] OR CORTICOID*[Text Word] OR STEROID*[Text Word] OR "PREDNISOLONE"[MeSH Terms] OR PREDNISOLONE[Text Word] OR "PREDNISONE"[MeSH Terms] OR PREDNISONE[Text Word] OR "DEFLAZACORT" [Supplementary Concept] OR DEFLAZACORT[Text Word] OR "METHYLPREDNISOLONE"[MeSH Terms] OR METHYLPREDNISOLONE[Text Word] OR "ANTIRHEUMATIC AGENTS"[Pharmacological Action] OR "ANTIRHEUMATIC AGENTS"[MeSH Terms] OR ANTIRHEUMATIC AGENT*[TW] OR ANTI-RHEUMATIC AGENT*[TW] OR ANTI RHEUMATIC AGENT*[TW] OR ANTIRHEUMATIC DRUG*[TW] OR ANTI-RHEUMATIC DRUG*[TW] OR ANTI RHEUMATIC DRUG*[TW] OR DMARD*[TW] OR DISEASE-MODIFYING ANTIRHEUMATIC DRUG*[TW] OR DISEASE MODIFYING ANTIRHEUMATIC DRUG*[TW] OR ANTIRHEUMATIC DISEASE-MODIFYING SECOND-LINE DRUG*[TW] OR ANTIRHEUMATIC DISEASE MODIFYING SECOND LINE DRUG*[TW] OR NON-STEROIDAL ANTI-RHEUMATIC AGENT*[Text Word] OR NONSTEROIDAL ANTI-RHEUMATIC AGENT*[Text Word] OR NON STEROIDAL ANTI-RHEUMATIC AGENT*[Text Word] OR NON-STEROIDAL ANTIRHEUMATIC AGENT*[Text Word] OR NONSTEROIDAL ANTIRHEUMATIC AGENT*[Text Word] OR NON STEROIDAL ANTIRHEUMATIC AGENT*[Text Word] OR NON-STEROIDAL ANTI RHEUMATIC AGENT*[Text Word] OR NONSTEROIDAL ANTI RHEUMATIC AGENT*[Text Word] OR NON STEROIDAL ANTI RHEUMATIC AGENT*[Text Word] OR "METHOTREXATE"[MeSH Terms] OR METHOTREXATE[Text Word] OR "CYCLOSPORINE"[MeSH Terms] OR CYCLOSPORINE[Text Word] OR CYCLOSPORIN[Text Word] OR CICLOSPORIN[Text Word] OR OL 27-400[Text Word] OR OL 27 400 [Text Word] OR OL 27400[Text Word] OR Tumor Necrosis Factor Inhibitors [Pharmacological Action] OR TNFi*[Text Word] OR TNF ALPHA INHIBITOR*[Text Word] OR TNF-ALPHA INHIBITOR*[Text Word] OR TNF INHIBITOR*[Text Word] OR TNF-INHIBITOR*[Text Word] OR TUMOUR NECROSIS FACTOR ALPHA INHIBITOR*[Text Word] OR TUMOUR NECROSIS FACTOR INHIBITOR*[Text Word] OR TUMOR NECROSIS FACTOR ALPHA INHIBITOR*[Text Word] OR TUMOR NECROSIS FACTOR INHIBITOR*[Text Word] OR ANTI TUMOR NECROSIS FACTOR AGENT*[Text Word] OR ANTI-TUMOR NECROSIS FACTOR AGENT*[Text Word] OR ANTI TUMOUR NECROSIS FACTOR AGENT*[Text Word] OR ANTI-TUMOUR NECROSIS FACTOR AGENT*[Text Word] OR ANTI-TNF ALPHA AGENT*[Text Word] OR ANTI TNF ALPHA AGENT*[Text Word] OR ANTI TNF AGENT*[Text Word] OR ANTI-TNF AGENT*[Text Word] OR "ADALIMUMAB"[MeSH Terms] OR ADALIMUMAB[Text Word] OR MONOCLONAL ANTIBODY D2E7[TW] OR D2E7 ANTIBODY[TW] OR "ETANERCEPT"[MeSH Terms] OR ETANERCEPT[Text Word] OR TNFR-FC FUSION PROTEIN[Text Word] OR TNFR FC FUSION PROTEIN[Text Word] OR TNF RECEPTOR TYPE II-IGG FUSION PROTEIN[Text Word] OR TNF RECEPTOR TYPE II IGG FUSION PROTEIN[Text Word] OR RECOMBINANT HUMAN DIMERIC TNF RECEPTOR TYPE II-IGG FUSION PROTEIN[Text Word] OR RECOMBINANT HUMAN DIMERIC TNF RECEPTOR TYPE II IGG FUSION PROTEIN [Text Word] OR TNT RECEPTOR FUSION PROTEIN[Text Word] OR TNR-001[Text Word] OR TNR 001[Text Word] OR TNR001[Text Word] OR "INFLIXIMAB"[MeSH Terms] OR INFLIXIMAB[Text Word] OR MAB CA2[Text Word] OR MONOCLONAL ANTIBODY CA2[Text Word] OR "GOLIMUMAB"[ Supplementary Concept] OR GOLIMUMAB[Text Word] OR CNTO 148[Text Word] OR CNTO148[Text Word] OR CNTO-148[Text Word] OR TsDMARD*[TW] OR TARGETED SYNTHETIC DMARD*[TW] OR TARGETED SYNTHETIC DISEASE-MODIFYING ANTIRHEUMATIC DRUG*[TW] OR TARGETED SYNTHETIC DISEASE MODIFYING ANTIRHEUMATIC DRUG*[TW] OR TOFACITINIB[Supplementary Concept] OR TOFACITINIB[Text Word] OR TASOCITINIB [Text Word] OR CP-690550[Text Word] OR CP-690,550[Text Word] OR CP 690550[Text Word] OR CP 690,550[Text Word] OR CP690550[Text Word] OR CP690,550[Text Word] OR BARICITINIB[Supplementary Concept] OR INCB-28050[Text Word] OR INCB028050[Text Word] OR INCB-028050[Text Word] OR LY3009104[Text Word] OR LY-3009104[Text Word] OR TOCILIZUMAB[Supplementary Concept] OR TOCILIZUMAB[Text Word] OR ATLIZUMAB[Text Word] OR BAT-1806[Text Word] OR BAT1806[Text Word] OR MSB-11456[Text Word] OR MSB11456[Text Word] OR RG-1569[Text Word] OR R-1569[Text Word] OR RHPM-1[Text Word] OR RO-4877533[Text Word] OR SARILUMAB[Supplementary Concept] OR SARILUMAB[Text Word] OR SAR-153191[Text Word] OR SAR153191[Text Word] OR REGN-88[Text Word] OR REGN88[Text Word] OR "RITUXIMAB"[MeSH Terms] OR RITUXIMAB[TW] OR IDEC-C2B8 ANTIBODY[Text Word] OR IDEC C2B8 ANTIBODY[Text Word] OR IDEC-C2B8[Text Word] OR IDEC C2B8[Text Word] OR GP2013[Text Word] OR ANAKINRA[Text Word] OR INTERLEUKIN 1 RECEPTOR ANTAGONIST PROTEIN[MeSH Terms] OR INTERLEUKIN 1 INHIBITOR[Text Word] OR IL-1 Inhibitor[Text Word] OR IL1 Inhibitor[Text Word] OR CANAKINUMAB[Supplementary Concept] OR CANAKINUMAB[Text Word] OR ACZ-885[Text Word] OR ACZ885[Text Word] OR ANTI-HUMAN INTERLEUKIN 1BETA[Text Word] OR RILONACEPT[Supplementary Concept] OR RILONACEPT[Text Word] OR INTERLEUKIN-1 TRAP [Text Word] OR "USTEKINUMAB"[MeSH Terms] OR USTEKINUMAB[TW] OR CNTO 1275[Text Word] OR CNTO-1275[Text Word]) OR CYCLOPHOSPHAMIDE[MeSH Terms] OR B-518[Text Word] OR B 518[Text Word] OR B518[Text Word] OR NSC-26271[Text Word] OR NSC 26271[Text Word] OR NSC26271[Text Word] OR THALIDOMIDE[Text Word] OR GAMMA GLOBULINS[Text Word] OR GAMMA-GLOBULIN[Text Word] OR GAMMA GLOBULIN[Text Word] OR Emapalumab[Supplementary Concept] OR Etoposide[MeSH Terms] OR NSC-141540[Text Word] OR NSC 141540[Text Word] OR NSC141540[Text Word] OR VP 16-213[Text Word] OR VP 16 213[Text Word] OR VP 16213[Text Word] OR VP-16[Text Word] OR VP 16[Text Word] OR VP16[Text Word] OR "BONE MARROW TRANSPLANTATION"[MeSH Terms])

**COCHRANE**

Web link: <https://www.cochranelibrary.com/>

Search strategy: The search strategy was done in three running searches.

**Cochrane. First search up to 02-15-2022. JIA Systemic**

ID Search

#1 (SYSTEMIC ARTHRITIS, JUVENILE):ti,ab,kw

#2 (ARTHRITIS, JUVENILE SYSTEMIC):ti,ab,kw

#3 (JUVENILE SYSTEMIC ARTHRITIS):ti,ab,kw

#4 (JUVENILE-ONSET STILL DISEASE):ti,ab,kw

#5 (JUVENILE ONSET STILL DISEASE):ti,ab,kw

#6 (STILL'S DISEASE, JUVENILE-ONSET):ti,ab,kw

#7 (JUVENILE-ONSET STILL'S DISEASE):ti,ab,kw

#8 (STILL'S DISEASE, JUVENILE ONSET):ti,ab,kw

#9 (STILL DISEASE, JUVENILE-ONSET):ti,ab,kw

#10 (STILL DISEASE, JUVENILE ONSET):ti,ab,kw

#11 (OR JUVENILE-ONSET STILLS DISEASE):ti,ab,kw

#12 (JUVENILE ONSET STILLS DISEASE):ti,ab,kw

#13 (STILLS DISEASE, JUVENILE-ONSET):ti,ab,kw

#14 (MACROPHAGE ACTIVATION SYNDROME):ti,ab,kw

#15 {or #1-#14}

#16 MeSH descriptor: [Glucocorticoids] explode all trees

#17 MeSH descriptor: [Steroids] explode all trees

#18 (GLUCOCORTICOID*):ti,ab,kw

#19 (CORTICOSTEROID*):ti,ab,kw

#20 (CORTICOID*):ti,ab,kw

#21 MeSH descriptor: [Prednisolone] explode all trees

#22 ("prednisolone"):ti,ab,kw

#23 MeSH descriptor: [Prednisone] explode all trees

#24 ("prednisone"):ti,ab,kw

#25 ("deflazacort"):ti,ab,kw

#26 MeSH descriptor: [Methylprednisolone] explode all trees

#27 ("methylprednisolone"):ti,ab,kw

#28 {or #16-#27}

#29 #15 and #28

#30 MeSH descriptor: [Antirheumatic Agents] explode all trees

#31 (ANTIRHEUMATIC AGENT*):ti,ab,kw

#32 (ANTI-RHEUMATIC AGENT*):ti,ab,kw

#33 (ANTI RHEUMATIC AGENT*):ti,ab,kw

#34 (ANTIRHEUMATIC DRUG*):ti,ab,kw

#35 (ANTI-RHEUMATIC DRUG*):ti,ab,kw

#36 (ANTI RHEUMATIC DRUG*):ti,ab,kw

#37 (DMARD*):ti,ab,kw

#38 (DISEASE-MODIFYING ANTIRHEUMATIC DRUG*):ti,ab,kw

#39 (DISEASE MODIFYING ANTIRHEUMATIC DRUG*):ti,ab,kw

#40 (ANTIRHEUMATIC DISEASE-MODIFYING SECOND-LINE DRUG*):ti,ab,kw

#41 (ANTIRHEUMATIC DISEASE MODIFYING SECOND LINE DRUG*):ti,ab,kw

#42 (ANTIRHEUMATIC DISEASE-MODIFYING SECOND-LINE DRUG*):ti,ab,kw

#43 (ANTIRHEUMATIC DISEASE MODIFYING SECOND LINE DRUG*):ti,ab,kw

#44 (NON-STEROIDAL ANTI-RHEUMATIC AGENT*):ti,ab,kw

#45 (NONSTEROIDAL ANTI-RHEUMATIC AGENT*):ti,ab,kw

#46 (NON STEROIDAL ANTI-RHEUMATIC AGENT*):ti,ab,kw

#47 (NON-STEROIDAL ANTIRHEUMATIC AGENT*):ti,ab,kw

#48 (NONSTEROIDAL ANTIRHEUMATIC AGENT*):ti,ab,kw

#49 (NON STEROIDAL ANTIRHEUMATIC AGENT*):ti,ab,kw

#50 (NON-STEROIDAL ANTI RHEUMATIC AGENT*):ti,ab,kw

#51 (NONSTEROIDAL ANTI RHEUMATIC AGENT*):ti,ab,kw

#52 (NON STEROIDAL ANTI RHEUMATIC AGENT*):ti,ab,kw

#53 MeSH descriptor: [Methotrexate] explode all trees

#54 ("methotrexate"):ti,ab,kw

#55 (CICLOSPORIN):ti,ab,kw

#56 MeSH descriptor: [Cyclosporine] explode all trees

#57 (OL 27400):ti,ab,kw

#58 MeSH descriptor: [Tumor Necrosis Factor Inhibitors] explode all trees

#59 (TNFi*):ti,ab,kw

#60 (TNF ALPHA INHIBITOR*):ti,ab,kw

#61 (TNF-ALPHA INHIBITOR*):ti,ab,kw

#62 (TNF INHIBITOR*):ti,ab,kw

#63 (TNF-INHIBITOR*):ti,ab,kw

#64 (TUMOUR NECROSIS FACTOR ALPHA INHIBITOR*):ti,ab,kw

#65 (TUMOUR NECROSIS FACTOR INHIBITOR*):ti,ab,kw

#66 (TUMOR NECROSIS FACTOR ALPHA INHIBITOR*):ti,ab,kw

#67 (TUMOR NECROSIS FACTOR INHIBITOR*):ti,ab,kw

#68 (ANTI TUMOR NECROSIS FACTOR AGENT*):ti,ab,kw

#69 (ANTI-TUMOR NECROSIS FACTOR AGENT*):ti,ab,kw

#70 (ANTI TUMOUR NECROSIS FACTOR AGENT*):ti,ab,kw

#71 (ANTI-TUMOUR NECROSIS FACTOR AGENT*):ti,ab,kw

#72 (ANTI-TNF ALPHA AGENT*):ti,ab,kw

#73 (ANTI TNF ALPHA AGENT*):ti,ab,kw

#74 (ANTI TNF AGENT*):ti,ab,kw

#75 (ANTI-TNF AGENT*):ti,ab,kw

#76 MeSH descriptor: [Adalimumab] explode all trees

#77 (ADALIMUMAB):ti,ab,kw

#78 (MONOCLONAL ANTIBODY D2E7):ti,ab,kw

#79 (D2E7 ANTIBODY):ti,ab,kw

#80 MeSH descriptor: [Etanercept] explode all trees

#81 ("etanercept"):ti,ab,kw

#82 (TNFR-FC FUSION PROTEIN):ti,ab,kw

#83 (TNFR FC FUSION PROTEIN):ti,ab,kw

#84 (TNF RECEPTOR TYPE II-IGG FUSION PROTEIN):ti,ab,kw

#85 (TNF RECEPTOR TYPE II IGG FUSION PROTEIN):ti,ab,kw

#86 (RECOMBINANT HUMAN DIMERIC TNF RECEPTOR TYPE II-IGG FUSION PROTEIN):ti,ab,kw

#87 (RECOMBINANT HUMAN DIMERIC TNF RECEPTOR TYPE II IGG FUSION PROTEIN):ti,ab,kw

#88 (TNT RECEPTOR FUSION PROTEIN):ti,ab,kw

#89 (TNR-001):ti,ab,kw

#90 (TNR 001):ti,ab,kw

#91 (TNR001):ti,ab,kw

#92 MeSH descriptor: [Infliximab] explode all trees

#93 (INFLIXIMAB):ti,ab,kw

#94 (MAB CA2):ti,ab,kw

#95 (MONOCLONAL ANTIBODY CA2):ti,ab,kw

#96 (GOLIMUMAB):ti,ab,kw

#97 (CNTO 148):ti,ab,kw

#98 (CNTO148):ti,ab,kw

#99 (CNTO-148):ti,ab,kw

#100 (TsDMARD*):ti,ab,kw

#101 (TARGETED SYNTHETIC DMARD*):ti,ab,kw

#102 (TARGETED SYNTHETIC DISEASE-MODIFYING ANTIRHEUMATIC DRUG*):ti,ab,kw

#103 (TARGETED SYNTHETIC DISEASE MODIFYING ANTIRHEUMATIC DRUG*):ti,ab,kw

#104 (TOFACITINIB):ti,ab,kw

#105 (TASOCITINIB):ti,ab,kw

#106 (CP-690550):ti,ab,kw

#107 (CP-690,550):ti,ab,kw

#108 (CP 690550):ti,ab,kw

#109 (CP 690,550):ti,ab,kw

#110 (CP690550):ti,ab,kw

#111 (CP690,550):ti,ab,kw

#112 (BARICITINIB):ti,ab,kw

#113 (INCB-28050):ti,ab,kw

#114 (INCB028050):ti,ab,kw

#115 (INCB-028050):ti,ab,kw

#116 (LY3009104):ti,ab,kw

#117 (LY-3009104):ti,ab,kw

#118 (TOCILIZUMAB):ti,ab,kw

#119 (ATLIZUMAB):ti,ab,kw

#120 (BAT-1806):ti,ab,kw

#121 (BAT1806):ti,ab,kw

#122 (MSB-11456):ti,ab,kw

#123 (MSB11456):ti,ab,kw

#124 (RG-1569):ti,ab,kw

#125 (R-1569):ti,ab,kw

#126 (RHPM-1):ti,ab,kw

#127 (RO-4877533):ti,ab,kw

#128 (SARILUMAB):ti,ab,kw

#129 (SAR-153191):ti,ab,kw

#130 (SAR153191):ti,ab,kw

#131 (REGN-88):ti,ab,kw

#132 (REGN88):ti,ab,kw

#133 MeSH descriptor: [Rituximab] explode all trees

#134 ("rituximab"):ti,ab,kw

#135 (IDEC-C2B8 ANTIBODY):ti,ab,kw

#136 (IDEC C2B8 ANTIBODY):ti,ab,kw

#137 (IDEC-C2B8):ti,ab,kw

#138 (IDEC C2B8):ti,ab,kw

#139 (GP2013):ti,ab,kw

#140 (ANAKINRA):ti,ab,kw

#141 (INTERLEUKIN 1 RECEPTOR ANTAGONIST PROTEIN):ti,ab,kw

#142 (INTERLEUKIN 1 INHIBITOR):ti,ab,kw

#143 (IL-1 Inhibitor):ti,ab,kw

#144 (IL1 Inhibitor):ti,ab,kw

#145 (CANAKINUMAB):ti,ab,kw

#146 (ACZ-885):ti,ab,kw

#147 (ACZ885):ti,ab,kw

#148 (ANTI-HUMAN INTERLEUKIN 1BETA):ti,ab,kw

#149 ("rilonacept"):ti,ab,kw

#150 ("interleukin-1 trap"):ti,ab,kw

#151 MeSH descriptor: [Ustekinumab] explode all trees

#152 (USTEKINUMAB):ti,ab,kw

#153 (CNTO 1275):ti,ab,kw

#154 (CNTO-1275):ti,ab,kw

#155 MeSH descriptor: [Cyclophosphamide] explode all trees

#156 (B-518):ti,ab,kw

#157 (B 518):ti,ab,kw

#158 (B518):ti,ab,kw

#159 (NSC-26271):ti,ab,kw

#160 (NSC 26271):ti,ab,kw

#161 (NSC26271):ti,ab,kw

#162 MeSH descriptor: [Thalidomide] explode all trees

#163 ("thalidomide"):ti,ab,kw

#164 (GAMMA GLOBULINS):ti,ab,kw

#165 (GAMMA-GLOBULIN):ti,ab,kw

#166 (GAMMA GLOBULIN):ti,ab,kw

#167 (Emapalumab):ti,ab,kw

#168 MeSH descriptor: [Etoposide] explode all trees

#169 (NSC-141540):ti,ab,kw

#170 (NSC 141540):ti,ab,kw

#171 (NSC141540):ti,ab,kw

#172 (VP 16213):ti,ab,kw

#173 (VP 16213):ti,ab,kw

#174 (VP-16):ti,ab,kw

#175 (VP 16):ti,ab,kw

#176 (VP16):ti,ab,kw

#177 {or #30-#176}

#178 #15 and #177

#179 MeSH descriptor: [Bone Marrow Transplantation] explode all trees

#180 #15 and #17

**SUPPLEMENTARY DATA 1 -PART 5: Study Selection, flowchart, included and excluded articles.**

The literature review initially identified 2874 manuscripts but finally, 73 were considered for the evidence report and only 11 for GRADE analysis. The following flowchart showed the process of searching, screening, and including articles.

1. **Flowchart studies**

**
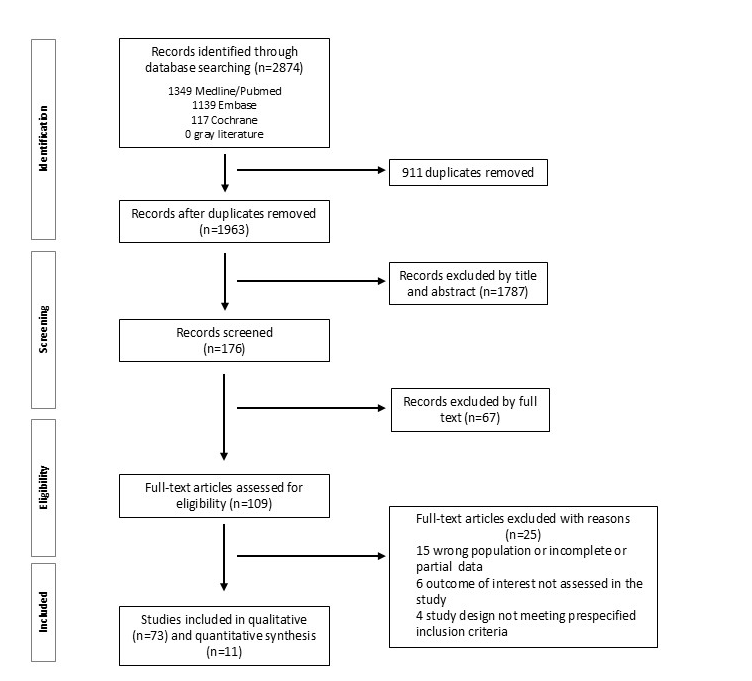
**

1. **Excluded Studies**

**Reasons of excluded studies**

**Characteristics of excluded studies**

***Vanucci 2013***

| Reason for exclusion | Review |
| --- | --- |

***Bullement 2021***

| Reason for exclusion | Wrong outcome |
| --- | --- |

***Calvo 2013***

| Reason for exclusion | Insufficient data |
| --- | --- |

***De Jager 2011***

| Reason for exclusion | Wrong outcome |
| --- | --- |

***Emminger 2013***

| Reason for exclusion | Insufficient data |
| --- | --- |

***Horneff 2018***

| Reason for exclusion | Wrong outcome |
| --- | --- |

***Sönmez 2018***

| Reason for exclusion | Wrong population |
| --- | --- |

***Brunner 2019***

| Reason for exclusion | Data in Ruperto 2021. |
| --- | --- |

***De Benedetti 2015***

| Reason for exclusion | Wrong outcome. |
| --- | --- |

***Le Stradic 2014***

| Reason for exclusion | Insufficient data |
| --- | --- |

***Yokota 2013***

| Reason for exclusion | Abstract of Yokota 2014. |
| --- | --- |

***Grom 2014***

| Reason for exclusion | Abstract, wrong outcome |
| --- | --- |

***Horneff 2017***

| Reason for exclusion | Abstract, full paper analyzed |
| --- | --- |

***Hur 2021***

| Reason for exclusion | Wrong outcome |
| --- | --- |

***Kostic 2021***

| Reason for exclusion | Abstract |
| --- | --- |

***Miettunen 2011***

| Reason for exclusion | Letter to the editor |
| --- | --- |

***Nakagishi 2016***

| Reason for exclusion | Case report |
| --- | --- |

***Orztuk 2015***

| Reason for exclusion | Case report |
| --- | --- |

***Quartier 2019***

| Reason for exclusion | Abstract, full paper analyzed |
| --- | --- |

***Ruperto(2) 2016***

| Reason for exclusion | Abstract, full paper analyzed |
| --- | --- |

***Ruperto 2011***

| Reason for exclusion | Abstract, full paper analyzed |
| --- | --- |

***Ruperto 2016***

| Reason for exclusion | Abstract, full paper analyzed |
| --- | --- |

***Wulffraat 2014***

| Reason for exclusion | Abstract |
| --- | --- |

***Wulffraat(2) 2014***

| Reason for exclusion | Abstract |
| --- | --- |

***Autmizguine 2014***

| Reason for exclusion | Wrong outcome |
| --- | --- |

**References**

**Excluded studies**

Vannucci G, Cantarini L, Giani T, Marrani E, Moretti D, Pagnini I, et al. Glucocorticoids in the management of systemic juvenile idiopathic arthritis. Pediatr Drugs. 2013; 15(5):343-9. doi: 10.1007/s40272-013-0038-0.

Bullement A, Knowles ES, Langenfeld M, Diogo GR, Nazir J, Eriksson D. An Economic Comparison of Treatment Strategies with Anakinra in Systemic Juvenile Idiopathic Arthritis (sJIA). Open Access Rheumatol. 2021; 13:257-266. doi: 10.2147/OARRR.S325400.

Calvo I, Marvillet I, Lόpez B, et al AB1158 Efficacy and security of anakinra treatment in patients with systemic-onset juvenil idiopathic arthritis: “Initial results from a register of two pediatric rheumatologic units from Spain”. Annals of the Rheumatic Diseases 2013;71:703.

de Jager W, Vastert S, Noordman B, et al. Anakinra restores the defective IL-18 NK cell axis in steroid naïve systemic onset JIA patients. Pediatr Rheumatol 9, O33 (2011). doi: 10.1186/1546-0096-9-S1-O33.

Emminger W, Dufek S, Perneczky-Hintringer E, et al. AB1218 Anakinra and canakinumab in children suffering from systemic onset juvenile idiopathic arthritis, from cryopyrin associated fever syndromes or from recurrences of pericardial effusion. Annals of the Rheumatic Diseases 2013;71:707.

Horneff G, Schulz A, Klein A, et al. THU0575 Anakinra for first line steroid free treatment in systemic onset juvenile idiopathic arthritis. Annals of the Rheumatic Diseases 2018;77:489.

Sönmez HE, Demir S, Bilginer Y, Özen S. Anakinra treatment in macrophage activation syndrome: a single center experience and systemic review of literature. Clin Rheumatol. 2018; 37(12):3329-3335. doi: 10.1007/s10067-018-4095-1.

Brunner H, Chen C, Martini A, Espada G, Joos R, Akikusa J, Chaitow J, Gámir Gámir M, Kimura Y, Rietschel C, Siri D, Smolewska E, Schmeling H, Brown D, De Benedetti F, Lovell D, Huang B, Ruperto N. Disability and Health-Related Quality of Life Outcomes in Patients with Systemic or Polyarticular Juvenile Idiopathic Arthritis Treated with Tocilizumab in Randomized Controlled Phase 3 Trials [abstract]. 2019.

De Benedetti F, Brunner H, Ruperto N, Schneider R, Xavier R, Allen R, Brown DE, Chaitow J, Pardeo M, Espada G, Gerloni V, Myones BL, Frane JW, Wang J, Lipman TH, Bharucha KN, Martini A, Lovell D; Paediatric Rheumatology International Trials Organisation and the Pediatric Rheumatology Collaborative Study Group. Catch-up growth during tocilizumab therapy for systemic juvenile idiopathic arthritis: results from a phase III trial. Arthritis Rheumatol. 2015; 67(3):840-8. doi: 10.1002/art.38984.

Le Stradic C, Galeotti C, Koné-Paut I, Traitement par tocilizumab: expérience d’un centre de rhumatologie pédiatrique 2014; 4321(12):1281-1402. doi: 10.1016/j.arcped.2014.08.018.

Yokota, S., Nozawa, T., Kanetaka, T. et al. PReS-FINAL-2166: Long-term safety and effectiveness of anti-interleukin-6 receptor monoclonal antibody, tocilizumab, in patients with systemic juvenile idiopathic arthritis in Japan. Pediatr Rheumatol 11, P178 (2013). doi :10.1186/1546-0096-11-S2-P178.

Grom A, Brunner H, Ruperto N, et al. FRI0528 Canakinumab in Systemic Juvenile Idiopathic Arthritis: Impact on the Rate and Clinical Presentation of Macrophage Activation Syndrome. Annals of the Rheumatic Diseases 2014; 73:578.

Horneff G, Dressler F, Thon A, Minden K. Real-life treatment with canakinumab in systemic juvenile idiopathic arthritis - first experience from the biker registry. Annals of the Rheumatic Diseases 217; 2(76):390. doi: 10.1136/annrheumdis-2017-eular.1745.

Hur P, Yi E, Ionescu-Ittu R, Manceur AM, Lomax KG, Cammarota J, Xie J, Gautam R, Nakasato P, Sanghera N, Kim N, Grom AA. Reasons for Initiating Canakinumab among Patients with Systemic Juvenile Idiopathic Arthritis and Adult-Onset Still's Disease in the U.S. Real-World Settings. Rheumatol Ther. 2022; 9(1):265-283. doi: 10.1007/s40744-021-00402-z.

Kostik M, Isupova E, Chikova I, et al. POS1310 Canakinumab is a rescue treatment for macrophage activation syndrome in patients with systemic onset juvenile idiopathic arthritis: single center experience. Annals of the Rheumatic Diseases 2021; 80:937.

Miettunen PM, Narendran A, Jayanthan A, Behrens EM, Cron RQ. Successful treatment of severe paediatric rheumatic disease-associated macrophage activation syndrome with interleukin-1 inhibition following conventional immunosuppressive therapy: case series with 12 patients. Rheumatology (Oxford). 2011; 50(2):417-9. doi: 10.1093/rheumatology/keq218.

Nakagishi Y, Shimizu M, Kasai K, Miyoshi M, Yachie A. Successful therapy of macrophage activation syndrome with dexamethasone palmitate. Mod Rheumatol. 2016; 26(4):617-20. doi: 10.3109/14397595.2014.906053.

Can Öztürk, Sümer Sütçüoğlu, Oya Baltalı, Meral Türker, Sezin Aşık Akman, Esra Arun Özer.Macrophage Activation Syndrome in Children with Systemic Juvenile Idiopathic Arthritis Successfully Treated with Megadose Methyl Prednisolone Therapy. The Journal of Pediatric Research 2015; 2(2):92-5.

Quartier P, Alexeeva E, Wouters C, et al. OP0055 efficacy of canakinumab, on a reduced dose or a prolonged dose interval without concomitant corticosteroids and methotrexate, in patients with systemic juvenile idiopathic arthritis. Annals of the Rheumatic Diseases 2019;78:98-99.

Ruperto N, Brunner H, Quartier P, et alTHU0215 Long-Term Efficacy and Safety of Canakinumab in Patients with Systemic Juvenile Idiopathic Arthritis (SJIA): 5-Year Follow-up of An Open-Label Trial. Annals of the Rheumatic Diseases 2016; 75:265-266.

Ruperto N, Brunner H, Horneff G, et al. Efficacy and safety of canakinumab, a long acting fully human anti-Interleukin-1β antibody, in systemic juvenile idiopathic arthritis with active systemic features: results from a phase III study. Pediatr Rheumatol Online J. 2011; 9(Suppl 1):O21. doi:10.1186/1546-0096-9-S1-O21.

Ruperto N.THU0579 Treating To Target with Canakinumab in Patients with Active Systemic Juvenile Idiopathic Arthritis: Results from The Long-Term Extension The Phase III Pivotal Trial. Annals of the Rheumatic Diseases 75(Suppl 2):401.2-402.

Wulffraat N, Ruperto N, Brunner H, et al. OP0180 Maintenance of Efficacy by Canakinumab Treatment in Systemic Juvenile Idiopathic Arthritis Patients. Annals of the Rheumatic Diseases 2014;73:130.

Wulffraat N, Ruperto N, Brunner H, et al. Canakinumab treatment shows maintained efficacy in systemic juvenile idiopathic arthritis patients. Pediatr Rheumatol 2014; 12:P68. doi: 10.1186/1546-0096-12-S1-P68.

Autmizguine J, Cohen-Wolkowiez M, Ilowite N; RAPPORT Investigators. Rilonacept pharmacokinetics in children with systemic juvenile idiopathic arthritis. J Clin Pharmacol. 2015; 55(1):39-44. doi: 10.1002/jcph.372.

**References of included studies**

Included studies are detailed in Supplementary Data 2 and 3.

**References**

1. Guyatt G, Oxman AD, Akl EA, Kunz R, Vist G, Brozek J, Norris S, Falck-Ytter Y, Glasziou P, DeBeer H, Jaeschke R, Rind D, Meerpohl J, Dahm P, Schünemann HJ. GRADE guidelines: 1. Introduction-GRADE evidence profiles and summary of findings tables. J Clin Epidemiol. 2011;64(4):383-94. doi: 10.1016/j.jclinepi.2010.04.026. [↑](#endnote-ref-2)
2. Guyatt GH, Oxman AD, Kunz R, Atkins D, Brozek J, Vist G, Alderson P, Glasziou P, Falck-Ytter Y, Schünemann HJ. GRADE guidelines: 2. Framing the question and deciding on important outcomes. J Clin Epidemiol. 2011;64(4):395-400. doi: 10.1016/j.jclinepi.2010.09.012. [↑](#endnote-ref-3)
3. Guyatt GH, Oxman AD, Santesso N, Helfand M, Vist G, Kunz R, Brozek J, Norris S, Meerpohl J, Djulbegovic B, Alonso-Coello P, Post PN, Busse JW, Glasziou P, Christensen R, Schünemann HJ. GRADE guidelines: 12. Preparing summary of findings tables-binary outcomes. J Clin Epidemiol. 2013;66(2):158-72. doi: 10.1016/j.jclinepi.2012.01.012. [↑](#endnote-ref-4)
4. Guyatt GH, Oxman AD, Vist G, Kunz R, Brozek J, Alonso-Coello P, Montori V, Akl EA, Djulbegovic B, Falck-Ytter Y, Norris SL, Williams JW Jr, Atkins D, Meerpohl J, Schünemann HJ. GRADE guidelines: 4. Rating the quality of evidence--study limitations (risk of bias). J Clin Epidemiol. 2011;64(4):407-15. doi: 10.1016/j.jclinepi.2010.07.017. [↑](#endnote-ref-5)
5. Guyatt GH, Oxman AD, Montori V, Vist G, Kunz R, Brozek J, Alonso-Coello P, Djulbegovic B, Atkins D, Falck-Ytter Y, Williams JW Jr, Meerpohl J, Norris SL, Akl EA, Schünemann HJ. GRADE guidelines: 5. Rating the quality of evidence--publication bias. J Clin Epidemiol. 2011;64(12):1277-82. doi: 10.1016/j.jclinepi.2011.01.011. [↑](#endnote-ref-6)
6. Guyatt GH, Oxman AD, Kunz R, Brozek J, Alonso-Coello P, Rind D, Devereaux PJ, Montori VM, Freyschuss B, Vist G, Jaeschke R, Williams JW Jr, Murad MH, Sinclair D, Falck-Ytter Y, Meerpohl J, Whittington C, Thorlund K, Andrews J, Schünemann HJ. GRADE guidelines 6. Rating the quality of evidence--imprecision. J Clin Epidemiol. 2011 Dec;64(12):1283-93. doi: 10.1016/j.jclinepi.2011.01.012. Epub 2011 Aug 11. Erratum in: J Clin Epidemiol. 2021;137:265. [↑](#endnote-ref-7)
7. Guyatt GH, Oxman AD, Kunz R, Woodcock J, Brozek J, Helfand M, Alonso-Coello P, Glasziou P, Jaeschke R, Akl EA, Norris S, Vist G, Dahm P, Shukla VK, Higgins J, Falck-Ytter Y, Schünemann HJ; GRADE Working Group. GRADE guidelines: 7. Rating the quality of evidence--inconsistency. J Clin Epidemiol. 2011;64(12):1294-302. doi: 10.1016/j.jclinepi.2011.03.017. [↑](#endnote-ref-8)
8. Guyatt GH, Oxman AD, Kunz R, Woodcock J, Brozek J, Helfand M, Alonso-Coello P, Falck-Ytter Y, Jaeschke R, Vist G, Akl EA, Post PN, Norris S, Meerpohl J, Shukla VK, Nasser M, Schünemann HJ; GRADE Working Group. GRADE guidelines: 8. Rating the quality of evidence--indirectness. J Clin Epidemiol. 2011;64(12):1303-10. doi: 10.1016/j.jclinepi.2011.04.014. [↑](#endnote-ref-9)
9. Guyatt GH, Oxman AD, Sultan S, Glasziou P, Akl EA, Alonso-Coello P, Atkins D, Kunz R, Brozek J, Montori V, Jaeschke R, Rind D, Dahm P, Meerpohl J, Vist G, Berliner E, Norris S, Falck-Ytter Y, Murad MH, Schünemann HJ; GRADE Working Group. GRADE guidelines: 9. Rating up the quality of evidence. J Clin Epidemiol. 2011;64(12):1311-6. doi: 10.1016/j.jclinepi.2011.06.004. [↑](#endnote-ref-10)
10. Zoom Video Communications, Inc. (2024). *Zoom (version 5.10.6)* [Software]. <https://zoom.us> [↑](#endnote-ref-11)
11. Brouwers MC, Kho ME, Browman GP, Burgers JS, Cluzeau F, Feder G, Fervers B, Graham ID, Grimshaw J, Hanna SE, Littlejohns P, Makarski J, Zitzelsberger L; AGREE Next Steps Consortium. AGREE II: advancing guideline development, reporting and evaluation in health care. CMAJ. 2010;182(18):E839-42. doi: 10.1503/cmaj.090449. [↑](#endnote-ref-12)
