# Supplementary material for: Pan American League of Associations for Rheumatology treatment recommendations for systemic juvenile idiopathic arthritis
Source: Rheumatol Adv Pract. 2025 Nov 11;9(4):rkaf087. doi: 10.1093/rap/rkaf087 (PMC12607261; doi:10.1093/rap/rkaf087)
Supplement: rkaf087_Supplementary_Data [file rkaf087_supplementary_data.zip › Supplementary_Data_2._Systemic_JIA._Summary_of_evidence.docx]

**Juvenile idiopathic arthritis (JIA): Systemics**

***SUMMARY OF EVIDENCE***

*This file only present information about clinical trials or observational studies that answer the PICO questions*

Contents

[ABREVIATIONS 4](#_Toc132063038)

[SUMMARY OF EVIDENCE 7](#_Toc132063039)

[SYSTEMICS 7](#_Toc132063040)

[STEROIDS 7](#_Toc132063041)

[cDMARD - Classic disease modifying antirheumatic drugs 7](#_Toc132063042)

[ETANERCEPT (ETA) - Recombinant human monoclonal antibody anti-TNFα 8](#_Toc132063043)

[ABATACEPT (ABA) - Inhibits T cell activation. Selective blocker of CD80/CD86 to CD28 receptors 17](#_Toc132063044)

[RITUXIMAB (RTX) - Murine/human chimeric anti-CD20 monoclonal antibody 17](#_Toc132063045)

[ANAKINRA (ANA) - Recombinant polypeptide antagonist of IL-1 receptor 18](#_Toc132063046)

[TOCILIZUMAB (TOC) - Recombinant humanized IgG1 anti-human IL-6 receptor monoclonal antibody 22](#_Toc132063047)

[CANAKINUMAB (CAN) - a selective, fully human, anti-interleukin-1β monoclonal antibody 34](#_Toc132063048)

[INTERFERON GAMMA (INF) - Recombinant interferon (IFN) gamma 39](#_Toc132063049)

[RILONACEPT (RILO) 40](#_Toc132063050)

[CYCLOSPORINE (CsA) 42](#_Toc132063051)

[STEM CELLS 43](#_Toc132063052)

[MACROPHAGE ACTIVATION SYNDROME (MAS) 46](#_Toc132063053)

[ANAKINRA 46](#_Toc132063054)

[CYCLOSPORINE 48](#_Toc132063055)

[ETOPOSIDE 52](#_Toc132063056)

[Steroids or IVIG 53](#_Toc132063057)

[TOCILIZUMAB 57](#_Toc132063058)

[REFERENCES 58](#_Toc132063059)

## ABREVIATIONS

ABA: abatacept

ACR: American College of Rheumatology

ADA: adalimumab

ADR: any drug reaction

ERA: entesitis related arthritis

ANA: anakinra

AE: adverse events

bDMARDs: biologic DMARDs

CAN: canakinumab

C-HAQ: Childhood Health Assessment Questionnaire

CI: confidence interval

csDMARDs: conventional synthetic DMARDs

DAS: Disease Activity Score

DMARDs: disease-modifying antirheumatic drugs

ESR: erythrocyte sedimentation rate

ETA: etanercept

EY: exposure years

GC: glucocorticoid

GOL: golimumab

HAQ: Health Assessment Questionnaire

IFN: interferon

IL-1i: interleukin-1 inhibitors

ILAR: International League of Associations for Rheumatology

INF: infliximab

IQR: interquartile range

JADAS: Juvenile Arthritis Disease Activity Index

JPsA: juvenile psoriatic arthritis

MAS: macrophage activation syndrome

MII: medically important infections

MTX: methotrexate

NOS: Newcastle-Ottawa Scale

NSAID: nonsteroidal anti-inflammatory drugs

nsNSAID: non-selective NSAID

o-JIA: oligoarticular juvenile idiopathic arthritis

OR: odds ratio

p-JIA: polyarticular juvenile idiopathic arthritis

PEY: patient exposure years

PY: patient year

RF: rheumatoid factor

ROM: range of motion

RTX: rituximab

RR: risk ratio

s-JIA: systemic juvenile idiopathic arthritis

SAE: serious adverse events

SC: subcutaneous

SD: standard deviation

SSZ: sulphasalazine

TNFi: tumor necrosis factor α inhibitor

TOC: tocilizumab

TOFA: tofacitinib

VAS: visual analogue scale

## SUMMARY OF EVIDENCE

## SYSTEMICS

# STEROIDS

Picco P, et al.^[[1]](#endnote-2)^ followed 22 patients affected by systemic JIA who underwent glucocorticoid (GC) treatment. The patients were randomized in two groups: one of them received methylprednisolone for 3 days followed by prednisone and the other group received prednisone. A remarkable decrease of disease activity and fever score and an improvement of joint involvement were observed. A CPR serum concentration decrease associated to a consistent increase of Hb over baseline values were disclosed in the group that received methylprednisolone. GC cumulative daily requirement was significantly lower in the group that received methylprednisolone.

# cDMARD - Classic disease modifying antirheumatic drugs

Methotrexate versus placebo was studied by Woo P, et al.^^[[2]](#endnote-3)^^ in systemic arthritis and extended oligoarticular arthritis. The analysis of global assessments of disease activity by the physician, parent, and patient showed no evidence of different effects of treatment in the two disease subgroups. But when the data from the two subgroups were combined, there was significant improvement in disease activity with MTX treatment according to the physicians (p<0.001), the parents (p<0.001), and the patients themselves (p< 0.02). Regarding the effect of treatment on systemic feature a reduction in the systemic feature score was seen during active treatment, but without significant differences. There was no significant difference in the effect of treatment on joint score and joint range of motion. Effect of treatment on CRP and ESR. With treatment, there were significant improvements in the CRP (p<0.001) and ESR (p<0.001). With treatment, there were significant improvements in the hemoglobin level, platelet count, white blood cell count, total protein level, and IgG level when data from the two disease subgroups were combined, and there were no significant differences between the treatment effects in the two subgroups. There was no significant reduction in the steroid dosage with MTX treatment.

# ETANERCEPT (ETA) - Recombinant human monoclonal antibody anti-TNFα

**Non comparative observational or single arms studies included**.

Giannini E, et al.^[[3]](#endnote-4)^ evaluated the long-term safety and effectiveness of ETA alone or in combination with MTX in patients with polyarthritis, systemic JIA, or extended oligoarthritis. Nevertheless, oligoarthritis is not described as partial results neither in the baseline table. Since the results correspond to the total group, it was not analyzed in RevMan and is shown in the table below.

Exposure-adjusted rates of adverse events were similar among the MTX, ETA, and ETA plus MTX groups were 18.3, 18.7, and 21.6 per 100 PY, respectively. Respective rates per 100 PY of SAE (4.6, 7.1, and 6.0) and medically important infections (1.3, 1.8, and 2.1) were also similar among groups. Scores for physician’s global assessment and total active joints improved from baseline, and improvement was maintained for the duration of the study.

| **Author, year**  **Methodology**  **Duration** | **Population Description** | **Treatment** | **Results** |
| --- | --- | --- | --- |
| Giannini 2009 ^3^  Phase IV, open-label, non randomized, multicenter study  Duration:  3 years  Data not discriminated.  Systemic JIA is not analyzed alone | JIA patients aged 2-18 years. However, 3 patients younger than 2 years of age were also included.  Patients were enrolled and classified at study entry using the JIA classification system.  . | **MTX alone:** n=197  >10 mg/m^2^/week [0.3 mg/kg/week], maximum dosage 1 mg/kg/ week  Systemic JIA: n=13, 6.6%  **Etanercept alone:** n=103  0.8 mg/kg/week, maximum dose 50 mg  Systemic JIA: n=8, 7.8%  **Etanercept plus MTX:** n=294 Systemic JIA: n=37, 12.6% | **245 completed the study (41%). 349 (59%) discontinued:**   - 103 (17%) discontinued due to lack of effectiveness: effectiveness (36 [18%], 8 [8%], and 59 [20%] in the MTX, ETA, and ETA plus MTX groups, respectively). - 6 (1%) discontinued due to AE: (3 [2%], 2 [2%], and 1 [0.3%] in the MTX, ETA, and ETA plus MTX groups, respectively). - Other causes: Two patients became pregnant during the study, and drug treatment was discontinued when the pregnancy was reported.   **Effectiveness***:*  **Physician’s global assessment and total active joint scores** for the 3 treatment groups were similar at baseline, and improvement was achieved in all groups at 3 and 6 months and was sustained through 36 months**.**  **Median physician’s global assessment scores** were 4 for MTX, 3 for ETA, and 4 for ETA plus MTX at baseline, 2 for all groups at month 3, 1 for MTX and 2 for both ETA groups at month 6, and 1 for all groups at months 12, 24, and 36.  **The median numbers of active joints** were 6 for MTX, 4 for ETA, and 6 for ETA plus MTX at baseline, 1 for MTX and 2 for both ETA groups at month 6, 0 for MTX, 1 for ETA, and 2 for ETA plus MTX at month 12, 0 for MTX and ETA and 1 for ETA plus MTX at month 24, and 0 for all groups at month 36.  **SAE:** The AE that led to study withdrawal were elevated liver enzymes (n=2) and rash (n=1) in the MTX group, juvenile dermatomyositis (n=1) and headache (n=1) in the ETA group, and injection site reaction (n=1) in the ETA plus MTX group. A total of 32 patients in the MTX group who discontinued switched to the ETA group (n=8) or ETA plus MTX (n)24) group. |

**Non comparative observational or single arms studies included**. **Table 1**

Lovell D, et al.^[[4]](#endnote-5)^ evaluated the safety and efficacy of up to 8 years of ETA treatment in patients with JIA previously enrolled in a RCT and included polyarticular, oligoarticular and systemic JIA. The results are not discriminated according to the course of the disease. The authors that the overall rate of SAs (0.12 PY) did not increase with long-term exposure to ETA. The rate of medically important infections (MII) was 0.03 PY; 1 new MII was reported in patients with >5 years of ETA exposure. No cases of tuberculosis, opportunistic infections, malignancies, lymphomas, lupus, demyelinating disorders, or deaths were reported. An ACR Pedi 70 response or higher was achieved by 100% of patients with 8 years of data and by 61% of patients according to the last observation carried forward data. ACR Pedi 30, 50, 70, 90, and 100 response rates were 83%, 77%, 61%, 41% and 18%, respectively.

Woerner A, et al.^[[5]](#endnote-6)^ studied retrospectively 77 patients with s-JIA. On a first biological agent, inactive disease was achieved in 37 patients, including 1 patient out of 12 patients on ETA, 26 patients out of 51 on ANA and 7 out of 10 on CAN. At last follow-up, 40 patients were in clinical remission (27 patients off steroids, 5 patients having never received steroid treatment), either on (n=29) or off (n=11) biological agents.

The British Society for Paediatric and Adolescent Rheumatology Etanercept Cohort Study (BSPAR-ETN) is a national prospective observational study established in 2004. Kearsley-Fleet L, et al.^[[6]](#endnote-7)^ analysed children with JIA starting ETA. Median JADAS-71 at baseline was 16.6 (IQR: 12.0-24.1). At 1 year this decreased to 3.7 (IQR: 0.6-9.3; p<0.001). When patients were grouped by ILAR category, JADAS-71 improved after 1 year of treatment in all ILAR subtypes, although this did not reach significant differences in patients with persistent o-JIA. Of the 496 children included in this analysis, 17 (3%) stopped ETA due to inefficacy, 9 (2%) due to AE and 7 (1%) for other reasons.

Patients with s-JIA documented in the BIKeR register, who were exposed to ETA, TOC or IL-1i were studied by Horneff G, et al.^[[7]](#endnote-8)^ The number/rate of patients without systemic symptoms increased with TOC (41/58%, 30/86%, 45/94%, 42/93%, 35/95%, 27/96%) and IL-1i (24/37%, 19/68%, 28/78%, 31/79%, 23/74%, 19/83%) at months 0, 3, 6, 12, 18 and 24 months, respectively. The intention-to-treat analysis revealed that JADAS-remission (JADAS-10 ≤ 1) was reached at month 3, 6, 12, 18 and 24 in 10/93, 21/105, 22/109, 16/110 and 18/109 patients in the ETA cohort, in 8/31, 16/44, 15/46, 15/43 and 15/41 in the TOC cohort and in 13/26, 16/31, 18/38, 16/32 and 14/29 in the IL-1i cohort, respectively. Minimal disease activity (MDA, JADAS-10 ≤ 3.8) was reached at month 3, 6, 12, 18 and 24 in 32, 35, 38, 31 and 29 patients in the ETA cohort, in 14, 27, 23, 26 and 18 patients in the TOC cohort and in 17, 21, 26, 20 and 18 patients in the IL-1i cohort, respectively. At month 6, the difference between the rate of patients on treatment with IL-1i and TOC reaching JADAS-10 MDA compared to ETA was significant (ETA:TOC, p<0.01; ETA:IL-1i, p<0.001) as was the rate of patients reaching JADAS-10 remission (ETA:TOC, p<0.05; ETA:IL-1i, p<0.001). No significant differences were detected in JADAS-10 MDA in patients treated with TOC compared to patients treated with IL-1i (OR = 1.06; 95% CI, 0.96–1.16; p=0.262) or in JADAS-10 remission (OR = 1.01; 95% CI, 0.94-1.09; p=0.783) adjusted for the propensity score. ACR-defined inactive disease was reached at month 3, 6, 12, 18 and 24 in 23/98, 29/114, 29/114, 25/117 and 27/113 in the ETA cohort, in 19/49, 29/63, 32/67, 24/53 and 20/60 in the TOC cohort and in 17/34, 28/43, 28/47, 20/38 and 19/34 in the IL-1i cohort, respectively. Discontinuations caused by inefficacy were significantly more frequent within the ETA cohort (43.4%) than within the TOC (8.5%) or IL-1i (21.7%) cohorts. However, TOC was more often discontinued due to intolerance compared to ETA or IL-1i.

The German BIKER registry has been prospectively documenting JIA patients treated with biologics since 2001. For this analysis patients with the diagnosis of systemic-onset JIA were evaluated by Klein A, et al.^[[8]](#endnote-9)^ The total exposure time to biologics 151 patients received ETA, 109 TOC, 71 ANA and 51 CAN. About 260 patients, 53% had only one treatment course and 47% had more than one treatment course with a biologic including the restart of the same biologic or the switch to another biologic. In all cohorts combined, 464 AE and 92 SAE were reported during exposure to ETA, TOC, ANA or CAN up to 90 days after last dose. The rates for AE and SAE were highest with CAN (AE 108.8/100 exposure years -EY-; SAE 20.3/ 100 EY) and TOC (AE 99.2/100 EY; SAE 20.9/100 EY), followed by ANA (AE 33.1/100 EY; SAE 6.6/100 EY) and ETA (AE 20.2/100 EY; SAE 3.5/100 EY). The combination of TOC and systemic steroids resulted in higher AE rates (127.5/100 EY vs 79.4/ 100 EY, p=0.002) and SAE rates (28.4/100 EY vs 15.6/ 100 EY, p=0.019) in comparison with TOC without systemic steroids. Regarding rates of AE, SAE and infections, there were no significant differences whether patients were treated with systemic steroid concomitant to either ANA or CAN. Macrophage activation syndrome (MAS) was reported in all cohorts, with higher frequency in patients with CAN (n=3; 3.2/100 EY) and TOC (n= 6; 2.5/100 EY). In the ETA cohort there were two cases of MAS (0.5/100 EY) and in the ANA cohort one case (0.8/100 EY). The differences were not significant.

Armaroli G, et al.^[[9]](#endnote-10)^ analysed data from the Biker Registry with those JIA patients who starting a biologic therapy and belonging to the ILAR-defined JIA categories and studied effectiveness and safety. A total of 2725 new ETA users with a diagnosis of JIA were registered. Of these, ETA was received as a first-line biologic by 95.8% and as monotherapy without concomitant MTX by 31.5%. After nine years on continuous treatment, 68.1% of patients presented minimal disease activity, 43.1% JADAS-defined remission on drug, and 36.6% ACR-inactive disease. On ETA, the mean JADAS10 decreased from 15.3±7.5 at baseline to 5.6±5.7 (p<0.0001) after 3 months and to 4.1 ± 5.3 (p<0.0001) after 12 months of treatment. Patients recruited in the most recent years achieved a lower JADAS10 after 12 months on ETA compared to those enrolled in the earlier years, although the difference did not reach significance (2.3±2.0 in 2018 compared to 6.9±7.2 in 2001; p=0.0637). JADAS-defined minimal disease activity (MDA; JADAS ≤ 3.8) was reached at months 3, 12, and 24 in 844 (45.9%), 990 (58.6%), and 734 (61.8%) ETA patients, and in 252 (63.0%), 120 (63.8%), and 49 (68.1%) patients after 5, 7, and 9 years. JADAS-remission (JADAS ≤ 1) was reached at months 3, 12, and 24 in 315 (17.2%), 591 (35.0%), and 449 (37.8%) patients, and in 175 (43.8%), 76 (40.4%), and 31 (43.1%) patients after 5, 7, and 9 years. Over the course of the years, the percentage of patients who reached JADAS-MDA and JADAS-remission following 12 months of ETA treatment increased, respectively, from 43.1 and 20.9% in 2001 to 72.8 and 45.6% in 2018. ACR-inactive disease was reached at months 3, 12, and 24 by 166 (25.0%), 248 (42.8%), and 180 (46.3%) ETA patients, and in 84 (46.9%), 43 (48.9%), and 15 (36.6%) patients after 5, 7, and 9 years. Improvement according to JIA-ACR30/50/70/90 criteria was reached in 74/64/45/24% of patients at month 3, in 81/75/61/42% of patients at month 12, and in 82/76/64/46% of patients at month 24. JIA-ACR30/50/70/90 response rates were 84/80/68/53% after 5 years, 82/79/69/57% after 7 years, and 82/79/71/54% after 9 years on ETA. Over 18 years of observation, ETA was discontinued by 1655 (60.7%) patients. The most common reason for discontinuation was remission (23.9%), followed by inefficacy (21.8%) and intolerance (7.1%). During 5988 PY of ETA exposure, a total of 2053 AE were reported to the registry. No significant difference in exposure-adjusted AE rates was observed between ETA (34.3/100 PY) and biologic-naïve patients (35.6/100 PY; p=0.3). SAE were significantly more frequent in the ETA cohort (3.8 versus 1.4/100 PY; p=0.0001). The incidence of serious infections was significantly higher in the ETA group (0.9 versus 0.2/100 PY; p=0.0001), while neutropenia rates were comparable in the two cohorts (0.07 vs 0.05/100 PY; p=0.8). Three and two malignancies were documented in the ETA and biologic-naïve groups, as well as three and one deaths, respectively.

Beukelman T, et al.**^[[10]](#endnote-11)^** evaluated JIA patients treated with ETA from the CARRA Registry. Patterns of ETA and MTX use were categorized as follows: combination therapy (ETA and MTX started concurrently), step-up therapy (MTX started first and ETA added later), switchers (MTX started and then stopped when or before ETA started), MTX add-on (ETA started first and MTX added later), and ETA only (no MTX use). Two thousand thirty-two of the five thousand six hundred forty-one patients with JIA met inclusion criteria (74% female, median age at diagnosis 6.0 years [interquartile range 2.0, 11.0]. Most patients (66.9%) were treated with a non-biologic DMARD, primarily MTX, prior to ETA. At 24 months overall ETA persistence was 66.3%, with lower persistence for s-JIA (42.5%). At 36 and 48 and months overall persistence rates were 49.4 and 37.3% respectively. There was no predominant treatment strategy for clinically active enthesitis. Patients with clinically active sacroiliitis were most likely to receive ETA alone (39.4%), followed by step-up therapy (24.2%), however the number of patients with sacroiliitis was small. ETA persistence among spondyloarthritis patients (enthesitis related arthritis and psoriatic JIA) varied by MTX initiation pattern, with higher ETA persistence rates in those who initiated combination therapy (68.9%) and switchers/ETA only (73.3%) patients compared to step-up (65.4%) and MTX add-on (51.1%) therapy.

Klotsche J, et al.^[[11]](#endnote-12)^ analysed data from two prospective registries, BiKeR and JuMBO. Both registries provide individual trajectories of clinical data and outcomes from childhood to adulthood in JIA patients treated with bDMARDs and csDMARDs. The authors reported that 1724 patients were treated first with ETA treatment course (338 with second, 54 with third ETA course). Similar rates of discontinuation due to ineffectiveness and AE could be observed for the first (19.4%/6.2%), second (18.6%/5.9%), and third (14.8%/5.6%) ETA course. A higher likelihood for discontinuing ETA due to achieving an inactive disease was significantly associated with a shorter duration between JIA onset and the start of ETA treatment (HR = 0.91, 95% CI 0.87-0.95), lower age at the start of ETA (HR = 0.92, 95% CI 0.89-0.96), higher response to therapy within the first 6 months (HR = 1.12, 95% CI 1.07-1.16), and persistent oligoarthritis (HR = 1.89, 95% CI 1.22-2.93), whereas patients with RF-positive polyarthritis (HR = 0.56, 95% CI 0.35-0.89) were less likely to discontinue ETA due to the onset of inactive disease. A total of 332 patients (+/−MTX, 19.3%) discontinued ETA after achieving remission with the first ETA course. Younger age (HR 1.08, p<0.001), persistent oligoarthritis (HR 1.89, p=0.004), and shorter duration between JIA onset and ETA start (HR 1.10, p<0.001), as well as good response to therapy within the first 6 months of treatment (HR 1.11, p<0.001) significantly correlated to discontinuation with inactive disease. Reoccurrence of active disease was reported for 77% of patients with mean time to flare of 12.1 months. Authors could not identify any factor correlating to flare risk. The majority of patients were re-treated with ETA (n=117 of 161; 72.7%) after the flare. One in five patients (n=23, 19.7%) discontinued ETA again after achieving an inactive disease and about 70% of the patients achieved an inactive disease 12 months after restarting ETA.

**Table 1. Etanercept**

| **Author, year**  **Methodology**  **Duration** | **Population Description** | **Treatment** | **Results** |
| --- | --- | --- | --- |
| Lovell 2008 ^4^  Open label single arm  Duration:  8 years | JIA patient starting ETA. All patients had active disease despite treatment with NSAID and MTX at baseline.  n=58  Polyarticular: 59%  Oligoarticular/systemic: 41%  Data not discriminated | Patients received SC injections of ETA 0.4 mg/kg twice a  week (maximum dose 25 mg per injection) or 0.8 mg/kg once a week (maximum dose of 50 mg/week). | 2 years: 69 patients.  4 years: 58 patients (72%)  8 years: 26 patients (45%)  **SAE:** 16 patients (23%)  ACR Pedi 70 response or higher was achieved by 100% of patients with 8 years of data (11 of 11) and by 61% of patients according to the last observation carried forward data (28 of 46).  ACR Pedi 30, 50, 70, 90, and 100 response rates were 83% (40 of 48 patients), 77% (36 of 47 patients), 61% (28 of 46 patients), 41% (19 of 46 patients), and 18% (8 of 45 patients), respectively. |
| Woerner 2015 ^5^  Retrospective  Duration: between 2005-2012. | s-JIA based on ILAR criteria who started their first biologic therapy | ETA, TOC and the interleukin-1 inhibitors anakinra and canakinumab (IL-1i), abatacept  Doses are not detailed  n= 77 patients | **Inactive disease:** 37 patients; ETA: 1/12, TOC: 2/2, ABA: 1/1, IL-1: Anakinra: 26/51 - Canakinumab: 7/10,  **Switch to second biologic:** 34/77  **Switch to third biologic:** 18/77  **Switch to fourth biologic:** 4/77  **Last follow-up**: 40/77  **AE:**  ETA (17 patients; 28.5 PY), ABA (3 patients; 9.6 PY), ANA (58 patients; 127.7 PY), CAN (30 patients; 48.7 PY), TOC (20 patients; 27.9 PY) |
| Kearsley-Fleet 2015 ^6^  Prospective observational study  Duration:  12-week (Part 1) | Children with JIA starting ETA treatment.  n, %  Systemic JIA: 73, 15%  p oligoarthritis: 12, 2%  e oligoarthritis: 81, 16%  polyarthritis: 224, 45%  PsA: 36, 7%  ERA: 35, 7%  Undifferentiated arthritis: 35, 7% | ETA 0.8 mg/kg once weekly (maximum 50 mg) | **JADAS-71**, median (IQR) baseline: systemic JIA: 22.3 (15.5-33.8) / JADAS-71, median (IQR) 1 year: systemic JIA: 5.3 (1.1-11.9)  ACR Pedi 30: systemic JIA: 69%  ACR Pedi 50: systemic JIA: 64%  ACR Pedi 70: systemic JIA: 48%  ACR Pedi 90: systemic JIA: 27%  MDA: 37%  Factors associated with improvement in disease activity: Independent predictors of achieving ACR Pedi 90 at 1 year included shorter disease duration [OR 0.91; 95% CI: 0.85-0.97], no concurrent oral corticosteroid use (OR 0.48; 95% CI: 0.29-0.80) and history of uveitis (OR 2.26; 95% CI: 1.08-4.71). Independent predictors of achieving MDA at 1 year included younger patients (OR 0.60; 95% CI: 0.38-0.95), and disease not treated with concurrent oral corticosteroids (OR 0.57; 95% CI: 0.35-0.93). |
| Horneff 2017 ^7^  Observational Biker register  Duration:  2000-2015 | s-JIA according to ILAR criteria, starting treatment with a biologic agent between 2000 and 2015. Patients were only included in analyses if they  had assessments at baseline and at least at one follow up visit. | ETA, TOC and the interleukin-1 inhibitors ANA and CAN.  Doses not detailed. | n=245 patients  ETA: 143, TOC: 60, IL1i (ANA: 38, CAN: 22)  ETA/TOC/IL1i  JADAS-remission (JADAS ≤1): 20%/37%/52%  Minimal disease activity (JADAS ≤3.8): 35%/61%/ 68%  ACR inactive disease: 24%/33%/56%.  AE: compared to ETA, rates of AE were significantly higher in the TOC cohort (RR 5.3 PY; p<0.0001) and serious AE were observed more frequently with TOC (RR 2.5; p<0.5) and IL1i (2.9; p<0.01).  There were 71 AE in the ETA cohort, 118 in the TOC and 81 in the IL-1i cohort. |
| Klein 2020 ^8^  Observational Biker register  Duration:  2001-2016 | s-JIA according to ILAR criteria.  The majority of documented patients with s-JIA in BIKER had been treated with one or more cycles of the following biologics: ETA, TOC, ANA and/or CAN. | ETA, TOC and the interleukin-1 inhibitors ANA and CAN.  Doses not detailed. | n=260 patients  ETA: 151, TOC: 109, IL-1 (ANA: 71, CAN: 51)  The rates for **AE** and **SAE** were higher with CAN (AE 108.8/100 EY; SAE 20.3/ 100 EY) and TOC (AE 99.2/100 EY; SAE 20.9/100 EY), followed by ANA (AE 33.1/100 EY; SAE 6.6/100 EY) and ETA (AE 20.2/100 EY; SAE 3.5/100 EY.  **Hepatic** **events**: one case of hepatic steatosis each in the TOC and CAN cohorts.  **Vasculitis events**: 2 events, one cutaneous panarteritis nodosa in a patient receiving TOC and one case of vasculitic skin changes in the ETA cohort.  **Thrombotic events**: 2 events occurred in the TOC cohort.  **Anaphylaxia**: in TOC treated patients (11 events in nine patients; 4.5/100 EY) and in one patient with CAN (1.1/100 EY). The higher risk in TOC patients was not significant after adjustment.  **MAS**: was reported in all cohorts, with higher frequency in patients with CAN (n=3; 3.2/100 EY) and TOC (n=6; 2.5/100 EY). In the ETA cohort there were two cases of MAS (0.5/100 EY) and in the ANA cohort one case (0.8/100 EY). The differences were not significant.  There was no case of bleeding disorder, gastrointestinal perforation, cardiovascular event, pregnancy, cerebral insult, systemic lupus erythematosus, hepatitis B-reactivation, arterial hypertension, sarcoidosis, serum sickness, pulmonary hypertension or amyloidosis. |
| Armaroli 2020 ^9^  Open label single arm  Duration:  2001-2019 | JIA patient starting a biologic therapy and belonging to the ILAR-categories.  n=2725  Systemic JIA: 146, 5.3%  Polyarthritis: 1127, 41.2%  p oligoarthritis: 120, 4.4%  e oligoarthritis: 570, 20.9%  ERA: 486, 17.8%  PsA: 191, 7%  Unclassified JIA: 85, 3.1%  Data not discriminated. | Mean ETA dose was 0.79 ± 0.21/kg/week once weekly in 56.9% of patients and twice weekly in 43.1% | **JADAS10, mean (±):** decreased from 15.3 ± 7.5 at baseline to 5.6 ± 5.7 (p<0.0001) after 3 months and to 4.1 ± 5.3 (p<0.0001) after 12 months of treatment.  **JADAS-defined minimal disease activity (MDA; JADAS ≤ 3.8):** was reached at months 3, 12, and 24 in 844 (45.9%), 990 (58.6%), and 734 (61.8%) ETA patients, and in 252 (63.0%), 120 (63.8%), and 49 (68.1%) patients after 5, 7, and 9 years.  **JADAS-remission (JADAS ≤ 1):** was reached at months 3, 12, and 24 in 315 (17.2%), 591 (35.0%), and 449 (37.8%) patients, and in 175 (43.8%), 76 (40.4%), and 31 (43.1%) patients after 5, 7, and 9 years.  **ACR-inactive disease**: was reached at months 3, 12, and 24 by 166 (25.0%), 248 (42.8%), and 180 (46.3%) etanercept patients, and in 84 (46.9%), 43 (48.9%), and 15 (36.6%) patients after 5, 7, and 9 years.  **JIA-ACR Pedi 30/50/70/90 criteria** was reached in:   - 74/64/45/24% of patients at month 3 - 81/75/61/42% of patients at month 12 - 82/76/64/46% of patients at month 24 - 84/80/68/53% after 5 years - 82/79/69/57% after 7 years - 82/79/71/54% after 9 years - ACR inactive disease: 36,6%   **SAE**, 226 serious AEs. (SAE, 3.8/100PY)   \| **AE:** 2053 53 AEs (34.3/100PY) \| \| --- \| |
| Beukelman 2021 ^10^  Open label single arm  Duration:  2015 - 2018 | JIA patient starting a biologic therapy and belonging to the ILAR-defined JIA categories  n=2032 patients | Mean ETA dose was 0.79 ± 0.21/kg/week once weekly patients and twice weekly.  Patterns of ETA and MTX use were categorized as follows:  Total n=1681   - **Combination therapy (ETA and MTX started concurrently)**   Oligoarthritis: 34,9.3%  p Oligoarthritis: 13, 8.2%  e Oligoarthritis: 15, 8.4%  PsA: 29, 21.6%  ERA: 23, 14.1%  Systemic: 4, 10%  Undifferentiated: 6, 17.6%   - **Step-up therapy (MTX started first and ETA added later)**   Oligoarthritis: 184, 50.3%  p Oligoarthritis: 74, 46.5%  e Oligoarthritis: 99, 55.6%  PsA: 59, 44%  ERA: 65, 39.9%  Systemic: 23, 57.5%  Undifferentiated: 15, 44.1%   - **Switchers (MTX started and then stopped when or before ETa started)**   Oligoarthritis: 34, 9.3%  p Oligoarthritis: 11, 6.9 %  e Oligoarthritis: 18, 10.1%  PsA: 4, 3%  ERA: 7, 4.3%  Sytemic: 1, 2.5%  Undifferentiated: 1, 2.9%   - **MTX add-on (ETA started first and MTX added later)**   Oligoarthritis: 29, 7.9%  p Oligoarthritis: 12, 7.5%  e Oligoarthritis: 15, 8.4%  PsA: 12, 9%  ERA: 13, 8%  Sytemic: 9, 22.5%  Undifferentiated: 1, 2.9%   - **ETA only (no MTX use)**   Oligoarthritis: 53, 14.5%  p Oligoarthritis: 36, 22.6 %  e Oligoarthritis: 12, 6.7 %  PsA: 25, 18.7%  ERA: 51, 31.3%  Sytemic: 1, 2.5%  Undifferentiated: 6, 17.6% | **Persistence (all treatments):**   \|  \| Month 12 \| Month 24 \| Month 36 \| Month 48 \| \| --- \| --- \| --- \| --- \| --- \| \| **JIA categories** \| \| \| \| \| \| Systemic arthritis \| 52.5 \| 42.5 \| 30 \| 17.1 \| \| **ETN persistence by MTX initiation pattern: all patients** \| \| \| \| \| \| Combination \| 82.2 \| 64.6 \| 48.8 \| 42.3 \| \| Step-up \| 86.9 \| 66.9 \| 49.8 \| 34.7 \| \| Switchers + ETN only \| 88.4 \| 69.4 \| 48.6 \| 42.6 \| \| MTX add-on \| 77.8 \| 54 \| 45.2 \| 27.7 \| \| **ETN persistence by MTX initiation pattern: ERA, and PsA** \| \| \| \| \| \| Combination \| 78.9 \| 60.1 \| 47.2 \| 47.2 \| \| Step-up \| 79.0 \| 55.2 \| 37.3 \| 32.7 \| \| Switchers + ETN only \| 83.3 \| 60.9 \| 57.4 \| 44.9 \| \| MTX add-on \| 68.0 \| 51.1 \| 35.8 \| 29.9 \|   Data are not discriminated. |
| Klotsche 2021 ^11^  prospective observational study  Duration:  3 years | Children with JIA starting ETA treatment. | ETA 0.8 mg/kg once weekly (maximum 50 mg) | - 1724 ETA as first treatment, 338 as second and 54 as third. - **Re-ocurrence of active disease**: 77% after discontinuation. - **Re-treatment with ETN:** 72,7%, good responders.   **Response to therapy during the first ETA course**   - A total of 446 patients (61.5%) were in a state of an inactive disease at the 12-month follow-up - Systemic JIA (n=18, 47.4%)   **Etanercept withdrawal after achieving inactive disease:** n, %. HR [95% CI]   - Systemic: 15, 4.5%. 0.83 [0.47 to 1.45] |

* Values were calculated as the number of patients with physician’s global assessment of 0 (on a scale of 0 [no symptoms] to 10 [severe symptoms]) or total active joint score of 0 enrolling into the registry who completed at least 1 evaluation while receiving methotrexate (MTX) or etanercept divided by the number of patients enrolling into the registry who completed at least 1 evaluation while receiving MTX or etanercept and had data available at each visit.

JADAS 71: Juvenile Arthritis Disease Activity Score 71 joints evaluated; SAE: serious adverse event; SC: subcutaneous; IV: intravenous; ILAR: International League of Associations for Rheumatology; SAE: serious adverse event; MDA: minimal disease activity; eoJIA: extended oligoarthritis JIA; p oligoarthritis: persistent oligoarthritis; e oligoarthritis: extended oligoarthritis; ERA: enthesitis related arthritis.

# ABATACEPT (ABA) - Inhibits T cell activation. Selective blocker of CD80/CD86 to CD28 receptors

Ruperto N, et al. ^[[12]](#endnote-13)^ described the efficacy of ABA based on data derived from the patients who entered the open-label long-term extension phase and reflect ˃21 months of treatment. By day 589, 90%, 88%, 75%, 57%, and 39% of patients treated with ABA achieved responses according to the ACR Pedi 30, 50, 70, 90, and Pedi 100 criteria, respectively. Similar response rates were observed among patients previously treated with placebo. Only in the ACR Pedi 100, and in the proportion of patients with inactive disease, were observed differences in the ABA group. No cases of tuberculosis and malignancies were reported. Pneumonia developed in 3 patients, and multiple sclerosis in 1 patient. The outcomes were also analyzed in polyarticular JIA. Only 11.8% of the total population was oligoarticular subtype and 20.9 systemic subtype.

# RITUXIMAB (RTX) - Murine/human chimeric anti-CD20 monoclonal antibody

**Non comparative observational or single arms studies included**.

Alexeeva EI, et al. ^[[13]](#endnote-14)^ included children aged from 2.3 to 17.0 years who had a diagnosis of severe systemic, polyarticular, and oligoarticular JIA for at least 1 year and did not respond to GC, NSAIDs, and at least two other immunosuppressants (including MTX) and were also non-responsive (AE or secondary inefficacy) to TNF inhibitors. Rituximab was administered as one intravenous infusion per week for 4 successive weeks (375 mg/m^2^ of body surface area per dose). The primary endpoint was an ACR Pedi 30 response at week 24. The secondary endpoints were ACR Pedi 50 and 70 responses and clinical remission at weeks 24, 48, 72 and 96. The study included 55 children with JIA (30 girls and 25 boys). All patients received the first course of rituximab treatment, 54 patients received the second courses of rituximab, 41 patients received the third course and 25 patients received four courses.

| **Author, year,**  **Study methodology**  **Duration** | **Population Description** | **Treatment** | **Results** |
| --- | --- | --- | --- |
| Alexeeva 2011 ^13^  Observational  Duration: 96 weeks | Children aged from 2.3 to 17.0 years who had a diagnosis of JIA for at least 1 year and did not respond to GC, NSAIDs, and at least two other immunosuppressants (including MTX) and were also non-responsive to TNF inhibitors. | Rituximab IV infusion per week for 4 successive weeks (375 mg/m^2^ of body surface area per dose). | **JIA ACR 30:** achieved in 98% of patients at week 24.  **JIA ACR 50:** 75% at week 48, 75% at week 52 and 96% at week 96.  **JIA ACR 70:** 70% at week 48, 75% at week 52 and 93% at week 96.  At 24 and 48 week, 25% and 52% of patients respectively achieved remission.  The number of systemic manifestations was significantly reduced at 12 weeks (p<0.001). The life-threatening manifestations carditis and polyserositis were resolved in all patients by week 12. The signs of carditis and polyserositis were resolved in all patients by week 12.  The proportion of patients with skin rash decreased from 55% at baseline to 10% at week 12 (p<0.001). The dimensions of liver and of spleen returned to normal range in 80% of patients. No fever was observed in 91% of patients and sub-febrile temperature persisted in six patients (9%).  At week 24, inactive joints (painful, stiff, swollen) in 65% of patients. After 48 weeks of treatment, 52% of patients achieved remission of arthritis.  A total of 101 AE were reported during the study. These included 67 mild, 22 moderate, and 12 SAE.  SAE included severe neutropenia with absolute neutrophil count below 500/μL and acute focal pneumonia. |

# ANAKINRA (ANA) - Recombinant polypeptide antagonist of IL-1 receptor

Quartier P, et al.^[[14]](#endnote-15)^ studied the efficacy of a 1-month treatment with ANA vs placebo each with 12 patients with s-JIA. After one month, patients taking placebo were switched to ANA. At 1-month 8/12 responders were receiving ANA and 1 responder receiving placebo (p=0.003). Ten patients from the placebo group switched to ANA; nine were responders at month 2. Between month 1 and 12, six patients stopped treatment owing to an AE (n=2), lack of efficacy (n=2) or a disease flare (n=2). Six patients developed a SAE. Four infections and one vertebral collapse had a favorable outcome and these five patients continued the trial. One patient stopped ANA owing to a sudden increase in serum transaminases at month 6.

Atemnkeng Ntam V, et al.^[[15]](#endnote-16)^ evaluated patients undergoing treatment with ANA who were documented in the German Biologics registry- BiKeR. Patients were grouped into two cohorts, first-line, and second-line patients. Firstline included patients who never received any sort of s-JIA treatment except for NSAIDs and steroids for a maximum of 3 days prior to ANA start (steroid free-cohort). Meanwhile, the second-line cohort included patients who had been pre-treated with more than 3 days of steroids, DMARDs or other biologics prior to ANA start. This study was not included in RevMan as comparation due to the great differences in treatments among patients in second-line cohort. Therefore, here we describe briefly the results. At month 3, JADAS minimal disease activity (MDA), JADAS remission and ACR inactive disease were observed with 42%/37% and 57% respectively in all ANA treated patients. After one year of treatment, these rates were 62%/45% and 65% respectively in all patients. Patients in both cohorts showed significant JADAS-10 improvement at last follow-up. The mean JADAS10 improved from 13.4 (first-line) and 11.4 (second-line) at baseline to 0.3 and 1.1 (p=0.02 and 0.0014) at last follow-up in 1st and 2nd-line cohort respectively. At last follow-up, JADAS MDA, JADAS remission and inactive disease were reached by 66.7%;50% and 50% of patients in first-line cohort and 60%;45%;70% in the second-line cohort with no significant differences. Regarding safety during ANA treatment and 90 days after last dose, 41 AE were recorded (44.4/100 patient exposure years (PEY) [95% CI 32.7-60.3]) in both cohorts. Of these, 8 qualified as serious (SAE: 8.7/100 PEY exposure years [95% CI 4-17.3]).

**Non comparative observational or single arms studies included.**

Zeft A, et al.^[[16]](#endnote-17)^ evaluated retrospectively the effect of ANA in 33 patients with s-JIA. Treatment was associated with decreases in corticosteroid dosage and sedimentation rate and increases in hemoglobin and albumin. There were decreases in large joint arthritis counts but not small joint counts after 3 to 4 months. Eight patients had periods of arthritis, 1 developed macrophage activation syndrome, and another Epstein Barr virus.

Nigrovic P, et al.^[[17]](#endnote-18)^ studied the safety and efficacy of ANA as first-line therapy for s-JIA. Among 46 patients meeting inclusion criteria, ANA monotherapy was used in 10 patients (22%), while 67% received corticosteroids and 33% received additional DMARDs. Outcomes were evaluated at a median follow-up interval of 14.5 months. Fever and rash resolved within 1 month in >95% of patients, while CRP and ferritin normalized within this interval in >80% of patients. Active arthritis persisted at 1 month in 39% of patients, at 3 months in 27%, and at >6 months of follow-up in 11%. Approximately 60% of patients, including 8 of 10 receiving ANA monotherapy, attained a complete response without escalation of therapy. Associated AE included documented bacterial infection in 2 patients and hepatitis in 1 patient. Tachyphylaxis was not observed.

Marvillet I, et al.^[[18]](#endnote-19)^ retrospectively reviewed the medical records of patients with s-JIA treated with ANA (n=22). They had been follow-up for 11-56 months. Fever and rash were resolved in 18 patients (82%) within the first 3 months. Active arthritis persisted at 3 months in 22% of patients, at 6 months in 14%, and at 12 months in 22%. Sixteen patients (70%), including 6 patients receiving ANA as first-line therapy, attained a complete response. ANA was stopped in one patient due a severe skin reaction and two patients due to infections: one severe pneumonia and one positive intradermoreaction. Ten episodes of macrophage activation syndrome were observed in 9 patients (40%), 8 episodes were present at the S-JIA diagnosis and 2 while receiving ANA.

Vastert S, et al.^[[19]](#endnote-20)^ studied 20 patients with s-JIA before systemic steroid after ANA treatment. The mean follow-up period was 32 months (range 12-54 months). At the 3-month time point, 85% of the patients showed an adapted ACR Pedi 90 response or had inactive disease; 75% of the patients achieved this response while receiving recombinant IL-1Ra alone. After 1 year, 17/20 patients met the criteria for clinically inactive disease, and 13 of these patients met these criteria while receiving monotherapy with recombinant IL-1Ra. However, because of persistent disease activity, 7/20 patients required additional therapy besides recombinant IL-1Ra. After 3 years, 10 (91%) of 11 patients met the criteria for disease remission, either while receiving (n=2) or not receiving (n=8) medication.

Lainka E, et al.^[[20]](#endnote-21)^ studied s-JIA patients who were recorded in the AID-registry. In 6 years, 202 patients with confirmed s-JIA were recorded in the AID-registry. Out of these, 111 children received therapy with ANA (n=84) and/or CAN (n=27). During the first 12 months patients were evaluated according to Wallace criteria (achievement of inactive disease 28/55 and 17/20, remission over 6 months under medication 13/55 and 7/20 cases). Over the whole period of time, clinical response was preserved in the majority of patients (ANA 54/80, CANA 20/27). Arthritis mostly persisted in polyarticular courses. During treatment with IL-1i concomitant medication could be tapered in about 15%. IL-1i was discontinued in 59/111 patients. 45 (15) AE in ANA (CANA) treated patients (19.7 (26.6) AE/100 ANA (CANA) exposure years, 95%CI: 14.4–26.4 (14.9–43.9)) were reported.

| **Author, year,**  **Study methodology**  **Duration** | **Population Description** | **Treatment** | **Results** |
| --- | --- | --- | --- |
| Zeft 2009 ^16^  Retrospective study  Duration:  Median duration of therapy was 6 months. | s-JIA according to ILAR criteria  In this retrospective study, follow-up visits on ANA had not been predesignated at identical time points. They divided values into those occurring either 1 to 2 or 3 to 4 months into treatment. | ANA  Doses, median (IQR)  1.6 mg/kg (0.8 –9.1 mg/kg).  n=33 | **Corticoids dose** (mg/kg), mean (SD): **Baseline:** 0.4 (0.39); **Months 1-2:** 0.17 (0.22); **Months 3-4:** 0.13 (0.18)  **ESR**, mean (SD): **Baseline:** 56 (24); **Months 1-2:** 21 (20); **Months 3-4:** 26 (24)  **N° of active joints**, mean (SD): **Baseline:** 9.3 (9.5); **Months 1-2:** 6 (9.2); M**onths 3-4:** 4.5 (6.8)  **Fatigue improvement**, n (%): 26/28 (93%)  **Fever improvement**, n (%): 7/7 (100%)  **Pain of swelling on site of injection**, n (%): 17/32 (53.2%)  **MAS:** 1 patient |
| Nigrovic 2011 ^17^  Retrospective study  Duration:  after receiving ANA, months median (IQR): 82.4 (44-172) | s-JIA patients according to ILAR criteria. | ANA alone: 21.7%  ANA + DMARDs: 10.9%  ANA + steroids: 45.7%  ANA + steroids + DMARDs: 21.7% | **Fever and rash resolved, %:** 30 days: 97%  **Normalized CRP, %:** 30 days: 84%  **Normalized ESR, %:** 30 days: 63%  **Complete response: 59%**  **Partial response: 39%**  **Lack of response: 2%**  Injection site reactions occurred in 44% of the 45 patients with evaluable data, leading to permanent discontinuation of drug in 1 patient.  They observed 11 episodes of MAS in 9 patients (20%), including 6 at presentation and 5 while they were receiving anakinra. |
| Marvillet 2011 ^18^  Retrospective study  Duration:  Follow-up: 11-56 months | s-JIA patients | ANA 1-3 mg/kg/day  n=22 | **Fever and rash improvement, n (%):** 3 months: 18 (82%)  **Active arthritis persisted, n (%): 1**2 months: 22%  **Complete response, n (%):** 16 (70%)  They observed 10 episodes of MAS in 9 patients (40%), 8 episodes were present at the s-JIA diagnosis and 2 while receiving anakinra. |
| Quartier 2011  Two phase study.  Phase I: randomised, double blinded, placebo-controlled study for 1 month.  Phase II: open label period for 11 months.  Duration: 12 months | s-JIA patients aged 2-20 years with failure to corticosteroid and active and severe systemic symptoms and/or arthritis. | **Phase I:**  Intervention: ANA 2 mg/kg SC daily, maximum 100 mg.  Control: Placebo  **Phase II:** 2 mg/kg SC daily, maximum 100 mg | **Any AE**, n, e/100 PY: 12 months: 89, 5.7/100 PY  **SAE** n, e/100PY: 12 months: 5 (0.33)  **Paint to injection**, n, e/100 PY: 12 months: 15, 0.99/100 PY  **Post-injection erythema**, n, e/100 PY: 12 months: 6, 0.40/100 PY  Sixteen patients reached months 12; among seven responders, six had stopped corticosteroid treatment and five of them had inactive disease. |
| Vastert 2014 ^19^  Prospective study  Mean follow-up after starting anakinra: 32 months (12-54) | s-JIA patients according to ILAR criteria.  ANA was used as initial therapy, after failure to respond to NSAID but before the  use of other DMARDs, systemic corticosteroids, or other biologic agents. | **Anakinra**  2 mg/kg/day via SC injections, with a maximum dosage of 100 mg/day.  n=20 | **ACR 70,** n (%): **12 months:** 18/20 (90%).  **ACR 90**, n (%): **12 months:** 17/20 (85%); **24 months:** 12/14 (86); **36 months:** 10/11 (91%).  **Clinically inactive disease**, n (%): **12 months:** 13/20 (66%).  **CRP**, median (IQR): **Baseline:** 176 (56-410); **1 years:** 6 (2-42); **2 years:** 5 (2-8); **3 years:** 3 (2-5).  No serious infection was reported. |
| Lainka 2021 ^20^  Retrospective study  Duration: 6 years | s-JIA patients according to ILAR criteria. | Anakinra | **Fever** %: **Baseline:** 90%; **Under ANA:** 28%  **Skin involvement** %: **Baseline:** 71%; **Under ANA:** 25%  **Patients with arthritis** %: **Baseline:** 70%; **Under ANA:** 46%  **CRP (mg/L)**, median (IQR): **Baseline:** 48.7 (0-531); **Under ANA:** 17.1 (0-178.4)  **ESR (mm/h)**, median (IQR): **Baseline:** 44.5 (1-106); **Under ANA:** 18 (1-125)  **Good response**, n(%): **12 months:** 50/63 (79%); **Last follow-up:** 54/80 (68%)  **AE**, n, e/100 PY: 45, 19.7/100 PY  **Discontinuation,** n (%): 59/111 (53%)  **MAS:** none |

NSAID: non-steroidal anti-inflammatory drugs. ANA: anakinra. IQR: interquartile range; SD: standard deviation; AE: adverse event; SAE: serious adverse event; ESR: erythrocyte sedimentation rate. CRP: C-reactive protein. * events/100 patients’ year.

# TOCILIZUMAB (TOC) - Recombinant humanized IgG1 anti-human IL-6 receptor monoclonal antibody

De Benedetti F, et al.^[[21]](#endnote-22)^ studied active s-JIA (duration of ≥6 months and inadequate responses to NSAIDs and GC) treated with TOC vs placebo. Efficacy in the double-blind phase: At week 12, significantly more patients who received TOC than those who received placebo met the primary outcome of a JIA ACR 30 response and an absence of fever (85% vs. 24%, p<0.001). Significant differences in all variables in the ACR core set for JIA were observed between the TOC and placebo groups. Significantly more patients in the TOC group than in the placebo group had a JIA ACR 70 response (71% vs. 8%, p<0.001) or a JIA ACR 90 response (37% vs. 5%, p<0.001). Systemic symptoms (fever and rash) and laboratory abnormalities (anemia, thrombocytosis, and hyperferritinemia) significantly improved with TOC. In the double-blind phase, more patients in the TOC group than in the placebo group had AE (66 vs. 18 patients), and infection developed in more patients in the TOC group than in the placebo group (41 vs. 11 patients). The rate of infection was 3.4 PY with TOC, 2.9 PY with placebo, and 3.0 PY during open-label treatment. In the double-blind phase, 4 SAE (including two infections) occurred in 3 patients in the TOC group, as compared with none in the placebo group.

Kostik M, et al.^[[22]](#endnote-23)^ evaluated retrospectively thirty-seven active s-JIA children who have failed treatment with corticosteroids and other DMARDs, treated with TOC every 2 weeks and 4 weeks TOC dosing. The median delay from diagnosis to use of TOC was 36.0 months (range 10.7-97.0). The median duration of TOC treatment was 665 (range 456–1000) days. MAS occurred before TOC was in 12 children (32.4%). During the trial GC was successfully discontinued in 21/26 (80.8) after a mean of 66.0 days (range 43.0-93.0). CsA was discontinued in 10/20 (50.0%) after a mean of 53 days (range 25-85) after achievement of improvement, MTX in 9/32 (28.1%) patients after a mean of 11.5 months (range 3.8-25.6). During the trial 11/33 (33.3%) patients accomplished the status of being only on the monotherapy of TOC. Compared to the patients assigned to the Q2W TOC treatment group, the patients assigned to the Q4W TOC group had a milder s-JIA course. The patients had higher levels of hemoglobin, total proteins, and serum albumins. They had lower white blood cell counts, % granulocytes, CRP, ESR, ferritins and LDH. TOC was discontinued in 11 patients during the trial. The reasons for stopping TOC were achievement of inactive diseases status, SAE and death. Inactive disease was reached in 12 patients, and TOC treatment was stopped in 7/33 (21.2%) children. SAE included infusion reactions and a diagnosis of early MAS.

Yokota S, et al.^[[23]](#endnote-24)^ evaluated 56 children with disease refractory to conventional treatment who were given three doses of TOC 8 mg/kg every 2 weeks during a 6-week open-label lead-in phase. Patients achieving an ACR Pedi 30 response, and a CRP of less than 5 mg/L were randomly assigned to receive placebo or to continue TOC treatment for 12 weeks or until withdrawal for rescue medication in a double-blind phase. At the end of the open-label lead-in phase, ACR Pedi 30, 50, and 70 responses were achieved by 91%, 86%, and 68% patients, respectively. Both CRP concentrations and ESR remained low in the TOC group, but increased in the placebo group after patients entered the double-blind phase. No deaths or cases of MAS occurred during the lead-in and double-blind phases of the study. Two SAE were reported during the open-label lead-in phase: one anaphylactoid reaction in a patient who tested negative for IgE-type anti-TOC antibodies and previously had had allergic reactions to aspirin and INF, and one case of gastrointestinal haemorrhage from diff use acute or chronic colonic ulceration in a patient with a history of chronic diarrhoea and rectal bleeding.

**Non comparative observational or single arms studies included**. **Table 2**

Yokota S, et al.^23^ did a study which consisted of three phases open-label lead-in phase of 6 weeks, a double-blind, randomised, placebo-controlled phase of 12 weeks, and an open-label extension phase of at least 48 weeks. The distribution of ACR Pedi 30, 50, and 70 responses at completion of the open-label lead-in phase was similar in the placebo and TOC groups, and median ESR values and CRP concentrations were low and much the same in both groups in the double-blind phase. At the last observation, the ACR Pedi 30, 50, and 70 response rates were seen in 91%, 86% and 68% of 56 patients, respectively. 86% of 56 patients had an improvement in their actual CRP concentrations to less than 5 mg/L and this reduction took place within 2 weeks of starting TOC. Overall 79% of 56 patients achieved both an ACR Pedi 30 and CRP concentrations of less than 5 mg/L at week 6. Moreover, every ACR Pedi variable showed a sustained response to TOC treatment. Similar to CRP concentrations, median ESR rapidly fell within 2 weeks. At the 48-week analysis in the open-label extension phase, 96% of 50 patients were still receiving TOC. Median duration of treatment for the 50 patients from the initial open-label lead-in phase was 61·1 (range 8·7-98·9) weeks; 48 of these patients completed 48-week assessments. The numbers of patients who achieved ACR Pedi 30, 50, and 70 responses at 48 weeks were 98%, 94%, and 90%, respectively. The median absolute change from baseline in ESR at week 48 was −34 mm/h. The median absolute change from baseline in CRP concentrations at the same time point was −43·1. No deaths or cases of MAS occurred during the lead-in and double-blind phases of the study. Two SAE were reported during the open-label lead-in phase: one anaphylactoid reaction and one case of gastrointestinal haemorrhage.

The long-term efficacy and safety of TCZ treatment through 144 weeks were presented by Yokota S, et al.^[[24]](#endnote-25)^ In total, 50 patients responding to TOC and needing further treatment entered the open-label extension. ACR Pedi 30/50/70 response rates were 83.9%/83.9%/75.0%, respectively, at week 144 (last-observation-carried-forward). Inactive disease was achieved in 57.1% at week 144. Initially observed clinical improvements were maintained through week 144.

De Benedetti F, et al.^21^ described the efficacy of TOC in the open-label extension phase study. The 59% of these patients had a JIA ACR 90 response and an absence of fever at week 52. The mean(±SD) count of active joints decreased to 2.8±6.5 at week 52, and 48% of the patients had no active joints. A total of 32% of the patients met the criteria for clinically inactive disease. The need for oral glucocorticoids decreased; 52% of the patients discontinued glucocorticoids, and the mean prednisone-equivalent dose decreased to 0.06±0.09 mg per kilogram per day.

Mihailova D, et al.^[[25]](#endnote-26)^ evaluated seven children in the age range 2-12 years (mean 6.3 years) with disease duration 1.2-9 years with active persistent s-JIA have been enrolled in one-year prospective study. All 7 patients had oligoarticular joint involvement and received previous treatment with NSAIDs, MTX and GC. 4/7 had concomitant therapy with ETA and 2/7 second DMARD. In all patients TOC was administered immediately after discontinuation of the biological treatment or the second DMARD. At the end of the 3rd month, ACR Pedi 30, 50, 70 and 90 responses were achieved by 7 (100%), 5 (71%), 1(14%) and 0 (0%) patients, respectively. By 6^th^ month the observed responses we as follow: ACR Pedi 30 - 7 (100%), ACR Pedi 50 - 7 (100%), ACR Pedi 70 - 5 (71%), ACR 90 - 1 (14%). At the end of 9^th^ month ACR Pedi 30, 50, 70 and 90 responses were achieved by 7 (100%), 7 (100%), 6 (86%) and 4 (57%) patients. All patients maintained these parameters by 12^th^ month. At the end of the observational period the linear growth velocity of all treated patients reached the average normal values per year of their age matched peers.

Kubota T, et al.^[[26]](#endnote-27)^ studied 28 s-JIA patients treated with 8 mg/kg of TOC for more than 1 year who were initially resistant to 3 times of consecutive weekly methyl-PSL pulse therapy in active phase and/or had been refractory to long-term oral GC therapy. 96% of the patients attained clinical remission with combination therapy of prednisolone <0.2 mg/kg/day and TOC every 3 weeks. Thereafter, 36% completed prednisolone-free remission, and 25% attained drug-free remission by discontinued TOC. Serum IL-6 decreased quickly in correlating with improvement of clinical symptoms after starting TOC.

[De Benedetti](https://www.ncbi.nlm.nih.gov/pubmed/?term=De%20Benedetti%20F%5BAuthor%5D) F, et al.^[[27]](#endnote-28)^ described the patients registered in the optional alternative dosing schedule in the TENDER study. Of these 39 patients, 20 patients lost clinically inactive disease status at different points along the alternative dosing schedule until the data review of May 2014. In these 20 patients, the time to loss of clinically inactive disease status ranged from 1.4 to 27.9 months from initiation of the optional alternative dosing schedule (n=4 on 3-week dosing; n=10 on 4-week dosing; n=6 off TOC). Risk for losing inactive disease status on the optional alternative dosing schedule was 62.5% (10/16) in patients on concomitant methotrexate and 43.5% (10/23) in patients not on it.

Malattia C, et al.^[[28]](#endnote-29)^ Described in a post hoc analyses of TENDER trial 112 patients with active, refractory s-JIA of ≥6 months’ duration and inadequate response to previous NSAIDs and oral GC were enrolled in TENDER. Patients were randomly to receive TOC or placebo every 2 weeks for 12 weeks. On average, patients with s-JIA did not experience noticeable progression of radiographic damage over 2 years of treatment with TOC.

Yokota S, et al. ^[[29]](#endnote-30)^ described 2 clinical studies of TOC in s-JIA and their long-term extension study. Mean duration of TOC treatment was 3.4 years (range, 0.04-6.22) and total exposure was 228 PY. In total, 9 patients withdrew from the study, 4 (6.0%) as a result of AE [2 because of an anaphylactoid reaction and 2 because of a gastrointestinal hemorrhage (previously reported in the phase III study)], 1 (1.5%) because of an unsatisfactory response, and 4 (6.0%) in response to the development of anti-TOC antibodies. No deaths were reported during the study period. Most AE (93.1%) were mild. SAE were reported in 32 patients (47.8%; 34.7 events/100 PY); the most common SAE were infections (13.2 events/100 PY), laboratory test abnormalities (5.3 events/100 PY), musculoskeletal and connective tissue disorders (3.9 events/100 PY), and gastrointestinal disorders (3.9 events/100 PY).

Horneff G, et al.^[[30]](#endnote-31)^ evaluated the efficacy and safety of TOC in 60 s-JIA patients of the BiKeR registry. Only 20.3% of patients were treated with TOC in the first two years of their disease. Most patients showed a significant response to treatment. At last documentation 62%/58%/50% reached a JIA ACR30/50/70 response. The mean JADAS10 showed a decrease from 17.5 to 6.0/3.0/4.0/3.0/4.0 after 3/6/12/18/24 months. The proportion of patients in remission (JADAS10≤1) at month 6, 12, 24 was 42%/25%/27%, and the proportion in JADAS minimal disease activity, MDA (JADAS10≤3,8) 55%, 35% and 55%. 5 AE related cytopenias (without MAS), 4 intolerance reactions. 3 infections were SAE (appendicitis, pneumonia, herpes zoster). 4 patients developed a MAS.

Yokota S, et al.^[[31]](#endnote-32)^ described s-JIA who initiated intravenous TOC in real-world settings in Japan. Patients were registered before initiating TOC treatment. A total 9.6% of patients (40/417) discontinued TOC treatment, including discontinuation because the treatment objective was met (2.6% -11/417-). The most common reasons for discontinuation for the remaining patients were AE (4.1% -17/417-) and insufficient response (1.4% -6/417-); 3.8% (16/417) were lost to follow-up. The overall incidence rate per 100 PY for SAE was 54.5. The most common SAE were infections and infestations, with an incidence rate of 18.2/100 PY. The second most common SAE were blood and lymphatic system disorders, with a rate of 9.8/100 PY.

Pacharapakornpong T, et al.^[[32]](#endnote-33)^ studied patients with s-JIA in an observational studied. Patients were divided into the early TOC treatment group and the late TOC treatment group. Patients in the early TOC treatment had a remission rate of 54.5%, whereas none in the late TOC treatment achieved remission. At the 12-month follow-up, 91% in the early TOC treatment group and 50% in the late TOC achieved ACR Pedi 70.

Glazyrina G, et al.^[[33]](#endnote-34)^ studied 18 children with s-JIA treated with TOC. Therapy duration was from 3 months to 5 years. During the TOC therapy a decrease in disease activity was observed in all patients (number of joints with active arthritis, number of joints with functional impairments, assessment of functional activity according to CHAQ questionnaire, activity assessment according to VAS, assessment of parents according to VAS). Clinical disease remission (according to ACR Pedi criteria ≥90%) was observed in 11 patients after 6-9 months of treatment. Remission duration is from 3 months to 4 years.

Roszkiewicz J, et al.^[[34]](#endnote-35)^ evaluated s-JIA patients treated with TOC. At 12-week assessment none of the patients complained of fever. The number of children who achieved ACR Pedi 30, 50 and 70 at this time point was 10, 10 and 3, respectively. At this time point all the children reached the state of clinically inactive disease according to Wallace criteria. In all patients a significant decrease of inflammatory marker values were observed. After 12 weeks of TOC therapy all the children were still receiving oral corticosteroids, but in 9 of them we were able to reduce their doses by at least 30%. Treatment effectiveness after 48 weeks: At 48 weeks of TOC treatment all of the patients had achieved ACR Pedi 30, 50 and 70, and half of them had reached ACR Pedi 90. There was no change in CRP and ESR median concentration between 24 and 48 weeks of treatment. Regarding AE, no tuberculosis or Pneumocystis jiroveci opportunistic infection was noted and Herpes simplex type 1 infection was rare (0.11/patient-year) during the period of TOC treatment.

Demir S, et al.^[[35]](#endnote-36)^ retrospectively evaluated JIA patients who were treated with TOC and followed up for two years. Twenty patients with the diagnosis of JIA were included in the study. However, two patients (one patient with polyarticular and one patient with s-JIA) could not continue TOC due to an anaphylactic reaction. Finally, 18 patients were evaluated, 38.9% had p-JIA, and 61.1% had s-JIA. All patients with s-JIA had persistent course of disease. Platelet counts, ESR and CRP levels, active joint counts, and JADAS71 were significantly decreased at the third month. Acute phase reactants (ESR and CRP), active joint counts, and JADAS71 were significantly decreased at the third month. No significant differences between the third and sixth months were detected. At three months, eight patients (72.7%) had an inactive disease, while the remaining three patients had high levels of CRP without presence of any clinical symptoms. During follow-up, thrombocytopenia in two patients, MAS in one patient, and the elevation of transaminase in one patient were observed. None of the patients had active pulmonary tuberculosis before initiation. Three patients had a TST above 10 mm and isoniazid prophylaxis was started. None of the patients had activation of tuberculosis during follow-up.

Ruperto N, et al.^[[36]](#endnote-37)^ studied patients with active (≥6 months) s-JIA or p-JIA who received IV TOC or placebo every 4 weeks (p-JIA) or every 12 weeks (s-JIA). For s-JIA patients, changes within 3 months of treatment initiation with TOC (baseline) were compared between TOC and placebo patients. S-JIA patients experienced clinically relevant improvement of physical function (CHAQ-DI) and reduction in pain (pain-global). Marked improvement in all CHAQ-DI domains over 2 years was observed with TOC treatment in both s-JIA and p-JIA patients; improvement rates in patient well-being (patient-global) were 87.7% in s-JIA patients and 83.4% in p-JIA patients. There was also significant improvement in most domains of HRQOL (CHQ-domain scores) in patients with s-JIA. S-JIA patients experienced marked improvement in mean scores from baseline to week 104 for pain/discomfort (31.7 to 75.3), self-esteem (61.0 to 76.0), mental health (62.1 to 76.8), and social limitation-emotional (52.6 to 86.2).

Mallalieu N, et al. ^[[37]](#endnote-38)^ studied safety and efficacy of TOC in 11 patients. Efficacy: JADAS-71 and component values improved during the main evaluation period in patients younger than 2 years. The median JADAS-71 was 20.5 at baseline, decreased to near minimal disease activity by day 57, and was at minimal disease activity by day 85 (week 12). The percentage of patients with fever or rash decreased over the course of the study from baseline to week 12. The decrease in fever and rash was rapid, with fever resolving in 70% of patients and rash resolving in 50% of patients as early as day 8, after the first dose. Safety: A higher percentage of patients younger than 2 years experienced AE that led to withdrawal (3 because of clinically confirmed SAE of hypersensitivity and 1 because of a non-serious AE of thrombocytopenia). During the main evaluation period, 3 of 11 (27.3%) patients experienced SAE; 2 patients reported 1 SAE each (hypersensitivity and urticaria). One patient reported 3 SAE (hypersensitivity, hand-foot and-mouth disease, and JIA flare). Total observation period: SAE were reported by 5 of 11 patients (45.5%).

Pardeo M, et al. ^[[38]](#endnote-39)^ performed a secondary analysis of the data from the 2 pivotal phase III clinical trials of TOC in patients with s-JIA and p-JIA to investigate variables possibly associated with reduced neutrophil counts and to evaluate their relationship to the development of serious and non-serious infections. One of the studies was TENDER and the other CHERISH. TENDER was a 5-year, phase III study that assessed the efficacy and safety of IV TOC in 112 patients with s-JIA aged 2 to 17 years. Patients were randomly assigned to receive TOC (12 mg/kg for body weight < 30 kg or 8 mg/kg for body weight ≥ 30 kg) or placebo every 2 weeks. CHERISH studied TOC in p-JIA or o-JIA. Median absolute neutrophil count decreased from 8.13 × 10^9^/l at baseline to 3.93 × 10^9^/l within 2 weeks of the initiation of TOC treatment, then stabilized and remained within the normal range up to week 104 in the group of patients with s-JIA absolute neutrophil count. Notably, 44.6% of patients with s-JIA had normal absolute neutrophil count or grade 1 neutropenia throughout the study. Grade 2 neutropenia occurred in 30.4%, grade 3 in 23.2%, and grade 4 in 1.8% of patients. Among patients with s-JIA, 13 infection AE (243.7/100 PY) were reported for 29.7% of patients during placebo exposure compared with 46 infection AE (267.6/100 PY) reported for 45.3% of patients during TOC exposure. By week 104, 102/112 (91.1%) patients reported a total of 570 infection AE (282.1/100 PY).

SC TOC dosing regimens for s-JIA and p-JIA was studied by Ruperto N, et al.^[[39]](#endnote-40)^ JADAS-71 improved in TOC-naive s-JIA and p-JIA patients treated with SC TOC, similar to improvements observed with IV TOC, indicating that comparable efficacy was achieved with the SC and IV formulations. Among all patients who had efficacy data at week 52, 3/43 (7.0%) children with s-JIA and 8/47 (17.0%) children with p-JIA were able to reach a status of moderate disease activity (JADAS-71 3.9-10.5), and 93.0% of the children with s-JIA and 74.5% with p-JIA were able to reach a status of a low disease activity. By week 52, 68.6% of s-JIA patients and 63.5% of p-JIA patients had inactive disease; 52.9% of s-JIA and 30.8% of p-JIA patients achieved clinical remission on treatment.

A total of 77 s-JIA patients with a median disease duration of 11 months were studied by Yan X, et al.^[[40]](#endnote-41)^ Patients were grouped based on the duration (before TOC treatment): group A ≤ 6 months and group B > 6 months. After 2 weeks of TOC treatment, fever, active arthritis, JADAS-27 score, white blood cell counts, ESR, and CRP levels had significantly relieved (p<0.05) in both groups. After the 12-week treatment, the effect of TOC in relieving active arthritis in group A was better than in group B (p<0.05). There was no difference between group A and group B in relieving fever, JADAS-27 score, white blood cell count, ESR, and CRP. All patients were followed up for at least 24 months. At the 1-year follow-up, there was no significant difference in the proportion of patients who achieved clinical remission, no clinical activity, and clinical activity period in both groups. Leukopenia was observed in 7 patients, including one leukopenia induced by streptococcal infection, two infusion reactions characterized by fever and cold chills, and facial blushing. Two patients experienced MAS, one at 3 months and the other at 6 months of TOC treatment.

**Table 2. Tocilizumab**

| **Author, year,**  **Study methodology**  **Duration** | **Population Description** | **Treatment** | **Results** |
| --- | --- | --- | --- |
| Yokota 2008 ^23^  This study consisted of three phases:  Phase I: an open-label lead-in phase of 6 weeks.  Phase II: a double-blind, randomised placebo-controlled phase of 12 weeks.  Phase III: open-label extension phase of at least 48 weeks. | Patients between 2 and 19 years of age diagnosed as having s-JIA based on ILAR criteria. | **Phase I:** TOC 8 mg/kg every 2 weeks during a 6-week  **Phase II:**  Intervention: TOC 8 mg/kg every 2 weeks during a 12-week  Control: placebo during a 12-week  **Phase III:** TOC 8 mg/kg every 2 weeks for at least 48 weeks. | **Phase I (n=56)**  **ACR 30**, n (%): 51(91%)  **ACR 50,** n (%): 48 (86%)  **ACR 70,** n (%): 38 (68%)  **N° of active joints**, median (IQR): **Baseline:** 4 (0-39); **6 weeks:** 0 (0-34)  **N° of joints with restricted motion**, median (IQR): **Baseline:** 4 (0-39); **6 weeks:** 0 (0-34)  **Physician global assessment**, median (IQR): **Baseline:** 52 (18-100); **6 weeks:** 85 (0-97)  **Parent global assessment**, median (IQR): **Baseline:** 53 (0-90); **6 weeks:** 13.5 (0-69)  **CHAQ**, median (IQR): **Baseline:** 0.88 (0-3); **6 weeks:** 0.38 (0-3)  **ESR**, median (IQR): **Baseline:** 44.5 (8-125); **6 weeks:** 4 (0-64)  **Phase III (n=48)**  **ACR 30**, n (%): 47(98%)  **ACR 50**, n (%): 45 (94%)  **ACR 70**, n (%): 43 (90%)  **SAE**, n: 13 |
| Yokota 2012 ^24^  Open label extension of Yokota 2008  Duration:  144 weeks | s-JIA (ILAR criteria) patients who complete first 48 weeks of an open label extension. | TOC 8 mg/kg every 2 weeks during | **ACR 30**, % (CI95%): 144 weeks: 83.9% (71.7%-92.4%)  **ACR 50**, % (CI95%): 144 weeks: 83.9% (71.7%-92.4%)  **ACR 70**, % (CI95%): 144 weeks: 75% (61.6%-85.6%)  **Inactive disease**, n (%): **144 weeks:** 32/56 (57.1%)  *Safety*  **AE**, e/100 PY: 795/100 PY. 93% were mild.  **SAE**, %, e/100 PY: 42.9%, 34.8 100 PY |
| De Benedetti 2012 ^27^  Two phases study (TENDER trial)  Phase I: Phase III, double blinded, randomized, parallel assignment (12 weeks).  Phase II: open-label extension (until 104 weeks).  Duration: up to 5 years. Data until 52 weeks. | Patients with s-JIA who completed phase I.  110 patients (73 previous tocilizumab and 37 previous placebo)  We analyzed phase II. | TOC 8 mg/kg (patients ≥30kg) or 12 mg/kg (patients <30kg) IV every 2 weeks.  Administration frequency may be reduced to every 3 and every 4 weeks, respectively, according to an optional alternative dosing schedule | **Mean count of active joint decrease**, mean (SD): 52 weeks: 2.8 (6.5)  **Patients with no active joints**, %: 52 weeks: 48%  **Patients with inactive disease**, %: 52 weeks: 32%  **CHAQ-DI score ≥ 0.75**: 52 weeks: from 82% (baseline) to 38%  **Glucocorticoids discontinuation**, %: 52 weeks: 52% |
| Mihailova 2013 ^25^  Prospective cohort  Duration: 1 year | Children in the age range 2-12 years with active persistent s-JIA. | TOC was administered immediately after discontinuation of the biological treatment or  the second DMARD in a dose of 8 mg/kg for patients ≥30 kg and 12 mg/kg for patients < 30 kg every two weeks. | **ACR 30, n(%): 3 months:** 7 (100%); **6 months:** 7 (100%); **9 months:** 7 (100%); **12 months:** 7 (100%)  **ACR 50, n(%): 3 months:** 5 (71%); **6 months:** 7 (100%); **9 months:** 7 (100%); **12 months:** 7 (100%)  **ACR 70, n(%): 3 months:** 1(14%); **6 months:** 5 (71%); **9 months:** 6 (86%); **12 months:** 6 (86%)  **ACR 90, n(%): 3 months:** 0 (0%); **6 months:** 1 (14%); **9 months:** 4 (57%); **12 months:** 4 (57%)  At the end of the 1st month they found complete resolution of the systemic symptoms and acute phase response in all patients. |
| Kubota 2013 ^26^  Retrospective  Duration: 4.1 years | s-JIA patients treated with TOC for more than 1 year who were initially resistant to 3 times of consecutive weekly methyl-prednisolone pulse therapy in active phase and/or had been refractory to long-term oral steroid therapy. | **Tapering protocol**  **Group 1:**  PSL dose gradually to less than 0.2 mg/kg/ day.  **Group 2:**  Prolongation of TOC interval from every 2 week to 3 week.  **Group 3:**  Discontinuation of prednisolone  **Group 4**  Discontinuation of TOC. | **Clinical remission, %**  **Group 1:** 96%  **Group 2:** 96%  **Group 3:** 36%  **Group 4:** 25% |
| De Benedetti 2014 ^27^  Post hoc analyses of TENDER trial.  Duration:  2 years | s-JIA patients of TENDER trial.  Patients had to be in the study for a minimum of 2 years  and had to achieve clinical inactive disease.  Among the 112 patients enrolled,  39 (35%) entered the optional alternative dosing regimen. | **Tocilizumab IV**  Prolongation of the time interval between TOC infusions from 2 weeks (standard interval)  to 3 weeks, then 4 weeks, with the option of terminating TOC after the discontinuation of any treatment. | **April 2013 data review**  **Inactive disease status, n(%):** 26/39 (67%)  **May 2014 data review**  **Inactive disease status, n(%):** 19/39 (49%); every 3 weeks: n, 3; every 4 weeks: n, 9  Discontinuation: n, 7 |
| Malatia 2014 ^28^  Post hoc analyses of TENDER trial.  Duration:  Data evaluated until 104 week. | s-JIA patients of TENDER trial. | TOC 8mg/kg (patients ≥30kg) or 12 mg/kg (patients <30kg) IV every 2 weeks.  Administration frequency may be reduced to every 3 and every 4 weeks, respectively, according to an optional alternative dosing schedule. | **aSH score (n=47)** median, (IQR): **52 weeks:** 0.00 (-8.7: 4); **104 weeks:** 0.50 (-7.5: 12)  **Poznanski score (n=33)** median, (IQR): **52 weeks:** 0.29 (-0.05: 1.05); **104 weeks:** 0.16 (-0.01: 1.04)  **ACR 70**, n(%): **52 weeks:** 92/106 (86.8%); **104 weeks:** 57/65 (87.7%)  **ACR 90**, n(%): 5**2 weeks:**  67/106 (63.2%); **104 weeks:** 46/65 (70.8%) |
| Yokota 2014 ^29^  Open label extension of two trial (Yokota 2005-2008)  Duration: 168 weeks | s-JIA (ILAR criteria) patients aged 2-19 years. | TOC 8 mg/kg  every 2 weeks | **ACR 30**, % (CI95%): 168 weeks: 80.3% (68.2-89.4)  **ACR 50**, % (CI95%): 168 weeks: 80.3% (68.2-89.4)  **ACR 70**, % (CI95%): 168 weeks: 75.4% (62.7-85.5)  **ACR 90**, % (CI95%): 168 weeks: 60.7% (47.3-72.8)  **ACR 100**, 168 weeks: 18% (9.4-30)  *Safety:*  **AE**, e/100 PY: 803/100 PY  **SAE**, %, e/100 PY: 47.8%, 34.7 100 PY  **Infections SAE** e/100 PY: 13.2/100 PY |
| Horneff 2015 ^30^  Prospective cohort, BIKeR registry.  Duration:  2011-2014 | s-JIA patient who had started TOC. | Tocilizumab  n=60 | **At last documentation**  **ACR 30:** 62%; **ACR 50:** 58%; **ACR 70:** 50%  **Remission % (JADAS10 ≤1): 6 months:** 42%; **12 months:** 25%; **24 months:** 27%  **MDA % (JADAS10 ≤3,8): 6 months:** 42%; **12 months:** 25%; 2**4 months:** 27%  **AE**, 100 PY (95%CI): 101.7 (81-127.8)  **SAE**, 100 PY (95%CI): 13.7 (7.4-25.5) |
| Yokota 2016 ^31^  Retrospective cohort  Duration: 52 weeks | Pediatric patients with s-JIA who initiated IV TOC in real-world settings in Japan between April 2008 and  February 2012.  In addition, patients who were previously enrolled in a clinical trial received TOC prior to its approval in 2008. | TOC 8 mg/kg once every  2 weeks. | **Normal CRP level, from baseline: 52 weeks: 99%**  **Fever, n(%): Baseline:** 137/251 (54.6%); **52 weeks:** 14/251 (5.6%)  **Rash, n(%): Baseline:** 108/251 (43%); **52 weeks:** 14/251 (5.6%)  **Systemic features score, mean (SD): Baseline:** 1.6 (1.7); **52 weeks:** 0.2 (0.6)  *Safety*  **TOC exposure 407 PY**  **Any AE**, e/100 PY: 224.3 100 PY  **Infections and infestations,** e/100 PY: 69.8/100 PY  **MAS**, e/100 PY: 6.4/100 PY  **Blood and lymphatic system disorders**, e/100 PY: 14/100 PY  **Hepatobiliary disorders**, e/100 PY: 10.1/100 PY  **SAE**, e/100 PY: 54.5/100 PY  **Serious infections**, e/100 PY: 18.2/100 PY  **Serious infusion-related reactions**, e/100 PY: 3.4/100 PY |
| Pacharapakornpong 2016 ^32^  Retrospective study  Duration: 12 months | s-JIA patients according to ILAR criteria. | TOC 8 mg/kg/dose every 2 weeks divided in two groups:  Early TOC (n=11): patients receiving TOC as the first line therapy or receiving TOC as soon as it was indicated  Late TOC (n=12): patients receiving TOZ more than 6 months after it was indicated. | **Remission %: Early TOZ:** 54.5%; **Late TOC:** 0%  **ACR 70**, n (%): **Early TOC:** 10 (91%); **Late TOC:** 6 (50%)  **N° of active joints**, median (IQR):**Early TOC: Baseline** 3 (2); **12 months:** 0 (0); **Late TOC: Baseline:** 6 (6); **12 months:** 0 (0)  **N° of limited joints**, median (IQR): **Early TOC: Baseline** 2 (2); **12 months:** 0 (0); **Late TOC: Baseline:** 3 (5); **12 months:** 2 (2)  **Parent global assessment**, median (IQR): **Early TOC: Baseline** 5.9 (5.6); **12 months:** 0 (0); **Late TOC: Baseline: 4**.8 (5.6); **12 months:** 0.5 (4.5)  **Physician global assessment**, median (IQR): **Early TOC: Baseline** 5 (4); **12 months:** 0.5 (1.5); **Late TOC: Baseline:** 7(5); **12 months:** 2.5 (3.9)  **ESR (mm/h)**, median (IQR): **Early TOC: Baseline** 73 (30); **12 months:** 8 (18); **Late TOC: Baseline:** 61 (56); **12 months:** 5 (5)  **CHAQ**, median (IQR): **Early TOC: Baseline** 0 (0.3); **12 months:** 0 (0); **Late TOC: Baseline:** 0.3 (0.7); **12 months:** 0 (0) |
| Glazyrina 2017 ^33^  Retrospective  Duration:  23 months | s-JIA patients (aged 2-17 years) according to ILAR criteria who had started TOC.  n=18 | IV TOC was every 2 or 4 weeks in dose of 12 mg/kg for children *<*30 kg or 8 mg/kg for children ≥30 kg. | **ACR 90**, n(%) 6-9 months: 11/18 (61.1%)  **N° of joints with active arthritis**, median (IQR): **Baseline:** 13.5 (6-15); **Last documentation:** 1 (0-2)  **N° of joints with functional impairments**, median (IQR): **Baseline:** 12.5 (6-15); **Last documentation:** 3 (0-3)  **CHAQ**, median (IQR): **Baseline:** 2.09 (2-2.5); **Last documentation:** 0.25 (0-0.5)  **Physician VAS**, mean (IQR): **Baseline:** 82 (75-90); **Last documentation:** 16 (10-20)  **Parents VAS**, mean (IQR): **Baseline:** 84 (80-90); **Last documentation:** 18 (10-20) |
| Roszkiewicz 2018 ^34^  Retrospective  Duration: 2011-2017 | s-JIA (ILAR criteria) with predominant systemic features, not responding adequately to at least a two-week course of systemic steroids in the dose 1–2 mg/kg/day of oral prednisone or 10–30 mg/kg/day of IV methylprednisolone,  s-JIA with nvolvement of at least five peripheral joints or two joints and coexisting fever, in whom the disease is active despite at least three-month treatment with systemic steroids and MTX or another immunosuppressive drug. | TOC 12 mg/kg/dose every 2 weeks in children *<*30 kg and 8 mg/kg/dose every 2 weeks in children ≥30 kg. | **ACR 70**, n(%): **12 weeks:** 3 (30%); **24 weeks:** 8 (80%); **48 weeks:** 10 (100%)  **N° of patients with active arthritis**, mean: **Baseline:** 3; **12 weeks:** 0; **24 weeks:** 0; **48 weeks:** 0  **N° of patients with LOM**, mean: **Baseline:** 6; **12 weeks:** 0; **24 weeks:** 0; **48 weeks:** 0  **Physician global assessment**, mean: **Baseline:** 40; **12 weeks:** 10; **24 weeks:** 5; **48 weeks:** 0  **Parents global assessment**, mean: **Baseline:** 75; **12 weeks:** 35; **24 weeks:** 20; **48 weeks:** 15  **CHAQ**, mean: **Baseline:** 1.275; **12 weeks:** 0.675; **24 weeks:** 0.55; **48 weeks:** 0.375  **ERS (mm/h), mean: Baseline:** 37; **12 weeks:** 3; **24 weeks:** 2; **48 weeks:** 2.5  **AE**  **Neutropenia grade 1**, e/PY: 0.77/PY  **Increase in liver enzymes**, e/PY: 3.33/ PY  **Tuberculosis, Pneumocystis jiroveci and MAS:** None |
| Demir 2019 ^35^  Single arm observational descriptive  Duration:  6 months | s-JIA patients treated with TOC.  Followed up: 2014-2016  Disease duration, years, median (min-max): 5.5 (0.5-12) | IV TOC, BW <30 kg: 10 mg/kg every 2 weeks or BW ≥30 kg 8 mg/kg every 2 weeks.  n=11 | **Erythrocyte sedimentation rate, mm/hour, median (min-max):**  Baseline: 48 (25-119); 3^rd^ month: 8 (2-28); 6^th^ Month: 10 (2-25)  **C-reactive protein, mg/dl, median (min-max):**  Baseline: 4.6 (2.3-1.9); 3^rd^ month: 0.2 (0.1-1.3); 6^th^ Month: 0.6 (0.1-1.9)  **Active joint count, median (min-max):**  Baseline: 5 (2-14); 3^rd^ month: 0 (0-2); 6^th^ Month: 0 (0-2)  **JADAS 71, median (min-max):**  Baseline: 15 (7-24); 3^rd^ month: 4 (1-9); 6^th^ Month: 3 (2-4) |
| Ruperto 2019 ^36^  Phase II open label extension of TENDER trial  Duration: 104 weeks | s-JIA patients of TENDER trial | TOC 8mg/kg (≥30kg) or 12 mg/kg (<30kg) every 2 weeks.  Administration frequency may be reduced to every 3 and every 4 weeks, respectively, according to an optional alternative dosing schedule | **CHAQ**, mean (SD): **Baseline:** 1.71 (0.8); **week 104:** 0.58 (0.72)  **Parent global assessment**, mean (SD): **Baseline:** 58.79 (23.65); **week 104:** 7.28 (11-76)  **Physician global assessment**, mean (SD): **Baseline:** 66.89 (17.98); **week 104:** 7.40 (10.36)  **Patient global assessment**, mean (SD): **Baseline:** 58.96 (22.94); **week 104:** 7.25 (10.86) |
| Mallalieu 2019 ^37^  Phase I: Open-label, single arm  Phase II: optional extension  Duration:  Phase I: 12 weeks  Phase II: 2 years | s-JIA according ILAR criteria younger than 2 years.  The patients had symptoms for ≥1 month before screening and had uncontrolled s-JIA despite treatment with GC and NSAIDs. Patients had to have ≥2 active joints with or without fever (defined as ≥38 °C) attributed to s-JIA | TOC dose of 12 mg/kg IV Q2W.  n=10 | **JADAS 71**, mean change of baseline (SD): 12 weeks: -13.9 (-2.7,10.1)  **N° of joints with LOM**, mean change of baseline (SD): 12 weeks: -3 (-9,0)  **Physician global VAS**, mean change of baseline (SD): 12 weeks: -44 (-69,-37)  **Parent global VAS**, mean change of baseline (SD): 12 weeks: -28 (-85,-21)  **N° of active joints**, mean change of baseline (SD): 12 weeks: -7 (-16,-2)  **Presence Fever and rash**, n/N (%): **Baseline:** 9/11 (81-8%); **12 weeks:** 1/7 (14.3%)  **Presence of rash**, n/N (%)**: Baseline:** 8/11 (72.7%); **12 weeks:** 1/7 (14.3%)  **CRP level**, mg/L, mean change of baseline (SD): 12 weeks: -74 (-1181, -13)  **ERS**, mm/h, mean change of baseline (SD): 12 weeks: -59 (-78, -6)  *Safety at entire study*  **AE**, n (%): 10 (90.9%)  **SAE**, n (%): 5 (45.5%)  **AE with fatal outcome**, n(%): 0  **AE leading to dose interruption**, n(%): 5 (45.5%)  **Infection AE**, n(%): 9 (81.8%)  **Infection SAE**, n (%): 1 (9.1)  **Serious hypersensitivity reaction**, n (%): 3 (27.3%). |
| Pardeo 2019 ^38^  Analyses of TENDER trial  Duration: 104 weeks | s-JIA patients of TENDER trial | TOC 8 mg/kg (≥30kg) or 12 mg/kg (<30kg) IV every 2 weeks.  Administration frequency may be reduced to every 3 and every 4 weeks, respectively, according to an optional alternative dosing schedule. | **AE,** 104 week  102/112 (91.1%) patients reported a total of 570 infection AE (282.1/100 PY)  22 serious infection AE developed in 20 patients (17.9%; 10.9/100 PY): 4 gastroenteritis and varicella, 3 pneumonia, 2 herpes zoster.  **Neutropenia**, n(%) 104 week:  Grade 0-1: 50 (44.6%) / Grade 2: 34 (30.4%) / Grade 3: 26 (23.2%) / Grade 4: 2 (1.8%) |
| Ruperto 2021 ^39^  Open-label, multicentre, pharmacokinetic,  pharmacodynamic and safety phase 1 b study  Duration: 52 week | Children aged 1-17 years with s-JIA according to ILAR criteria, with inadequate responses to NSAIDs and GC.  Eligible patients were TOC-naive or had achieved well controlled s-JIA with IV TOC (TOC-prior). | TOC (162 mg/dose) every week or every 2 weeks every 10 days before interim analysis with body weight >30 kg or <30 kg | **52 weeks**  **Remission n (%) (JADAS10≤1):** 35/51 (63.5%)  **MDA% (JADAS10≤3.8):** 40/43 (93%)  *Safety*  **TCZ-naïve: patients with ≥1 AE**, n (%)**:** 26 (100%)  **Total AE**, n: 300  **Patients with ≥1 SAE**, n (%): 4 (15.4)  **Infections and infestations**, n (%): 21(80.8%)  **TCZ-prior:**  **Patients with ≥1 AE**, n (%): 24 (96%)  **Total AE**, n: 261  **Patients with ≥1 SAE**, n (%): 3 (12%)  **Infections and infestations**, n(%): 19(76%) |
| Yan 2021 ^40^  Retrospective  Duration: 52 weeks | s-JIA according ILAR criteria treated by TOC    Group A: <6 months  Group B: >6 months | IV TOC 12 mg/kg for body  weight <30 kg, 8 mg/kg for body weight ≥ 30 kg every 2 weeks.  After 12 weeks, TOC was given every 4 weeks and every 6 weeks after an initial 24 weeks of treatment. | **Fever,** n/N  **Group A: Baseline:** 40/41; **12 weeks:** 6/41  **Group B: Baseline:** 29/36; **12 weeks:** 4/36  **N° of active arthritis**, mean (SD)  **Group A: Baseline:** 3.2 (3)**; 12 weeks:** 0.7 (1.6)  **Group B: Baseline:** 4.4 (3.7); **12 weeks:** 1.1 (1.9)  **JADAS 27**, mean (SD)  **Group A: Baseline:** 21.7 (4.2)**; 12 weeks:** 4.9 (6.1)  **Group B: Baseline:** 21.8 (5.8); **12 weeks:** 5.2 (5.2)  **ESR (mm/H)**, mean (SD)  **Group A: Baseline:** 91.5 (30.6)**; 12 weeks:** 26.2 (40)  **Group B: Baseline:** 76.8 (28); **12 weeks:** 13.3 (22.8)  **CRP (mg/L)**, mean (SD)  **Group A: Baseline:** 78.4 (42.3); **12 weeks:** 19.1(24.3)  **Group B: Baseline:** 89.7 (33.7); **12 weeks:** 17.8 (28.5)  *Safety:* **Leukopenia**, n: 7; **Elevated transaminases**, n: 5; **Infusion reaction**, n: 3 |

aSH score: Sharp/van der Heijde score; IQR: interquartile range; SD: standard deviation; AE: adverse event; SAE: serious adverse event; MDA: minimal disease activity. CHAQ: Childhood Health Assessment Questionnaire. VAS: visual analogue scale. TCZ: tocilizumab. MAS: macrophage activation syndrome. LOM: limited of motion. ERS: erythrocyte sedimentation rate. CRP: C-reactive protein.

# CANAKINUMAB (CAN) - a selective, fully human, anti-interleukin-1β monoclonal antibody

Ruperto N, et al.^[[41]](#endnote-42)^ assessed the efficacy and safety of CAN in two trials. They published the results of the pivotal phase III trials. CAN treatment showed significant differences vs placebo in ACR Pedi 30/50/70/90 and in clinical inactive disease in trial 1, with significant differences in AE. In trial 2 when CAN was compared with placebo there were no differences in ACR Pedi 30 and 50, but they found differences in ACR 90. Regarding safety, MAS occurred in 7 patients; and infections were more frequent with CAN than with placebo. There were no cases of cancer, tuberculosis, or opportunistic infection in either trial.

Of the 177 patients enrolled in trial 2, a total of 144 (81%) continued in the long-term extension study (Ruperto N, et al.^[[42]](#endnote-43)^); 122 (69%) stayed for a maximum of 271 weeks. Overall, 75 (42%) patients completed the LTE study. When early vs late responders were compared, JADAS, fever and discontinuation rate showed significant differences. Non-response (n=26/102, 25%) and loss of response over time (n=36/102, 35%) were the main reasons for discontinuation with majority coming from the late responder’s group (n=25/36, 69%), followed by intolerance (n=19/102, 19%). Seven patients (n=7/102, 7%) discontinued CAN as per the physicians’/family decision no longer requiring CAN. For the patients who entered the long-term extension study from the open-label part of trial 2 (n=48), a decrease in disease activity by a median of 64.6% and 93.5% was observed within the 6 months and 2 years of CAN treatment, respectively. JIA-ACR 50/70/90 response rates in the ITT population at 6 months since initiation of CAN were 73.4%, 65.5% and 52.0%, respectively. This level of response was maintained up to 3 years with 54.8%, 53.7% and 49.7% of the patients achieving JIA-ACR 50/70/90 response rates, respectively. When JADAS, fever, and discontinuation rates were analyzed they found significant differences between early and late responders.

Alexeeva E, et al.^[[43]](#endnote-44)^evaluated CAN naïve patients compared switching to CAN from TOC. No differences were found in ACR Pedi 90 and clinical inactive disease between the groups.

Quartier P, et al.^[[44]](#endnote-45)^ published the results of a 2-part phase IIIb/IV open-label, randomized trial. Among the 75 randomized patients, clinical remission was maintained for 24 weeks in 27 (71%) of 38 patients in arm 1 (2 mg/kg every 4 weeks) and 31 (84%) of 37 patients in arm 2 (4 mg/kg every 8 weeks) (p≤0.0001 for arm 1 versus arm 2 among those meeting the 40% threshold). Overall, 25 (33%) of 75 patients discontinued CAN, and clinical remission was maintained for at least 24 weeks in all 25 of these patients. There were no differences at clinical remission at weeks 4, 8 and 27, founding only differences at week 12.

**Non comparative observational or single arms studies included.**

Horneff G, et al.^[[45]](#endnote-46)^ evaluated the long-term efficacy and safety profile of CAN-naïve s-JIA patients with and without s-JIA-associated fever. Data on 122/267 patients, 53 (43%) with and 69 (57%) without s-JIA associated fever, were available for analysis with a median 94-week study duration. At week4, ~75% of both subgroups had responded (≥ ACR JIA 30), increasing to 90% at week 12. At week 2, ~21% of both subgroups had inactive disease; 44% at week 8; 60% at week 20 and then 60-70% for the remainder of the trial. CRM was achieved in about 29% of patients in both subgroups with ~22% maintaining it for ≥12 consecutive months. At baseline, the median JADAS score was 21.5 with 8 (7.5%) and 99 (92.5%) patients meeting the criteria for moderate (JADAS >3.8 and ≤10.5) and high disease activity (JADAS >10.5), respectively. At day 15, the median JADAS was 6.8 and 1.5 at the last assessment. At the last assessment, 53 (48%) patients had inactive disease (JADAS ≤ 1); 10 (9%) with low active disease activity (JADAS >1 and ≤3.8); while 14 (13%) had moderate and 31 (28%) with high disease activity. Infection (0.56 infections/100 patient-days), typically involving upper respiratory tract was the most common type of AE. Fifteen patients discontinued due to an AE and 40 had >1 SAE (mostly infections, MAS, or flare-associated) and no deaths. Eight cases of MAS (0.013 events/100 patient days) were reported.

Ruperto N, et al.^[[46]](#endnote-47)^ published the first trial of CAN in a pediatric arthritis population establishing pharmacodynamics and pharmacokinetics parameters for the use of SC CAN in s-JIA patients. Phase II trial of CAN for the treatment of active s-JIA, was a multicenter open-label study, patients between 4 and 20 years of age with a body weight of at least 12 kg. The study defined s-JIA by the ILAR classification criteria and additionally required at least 6 months of disease duration; they defined active systemic disease as the presence of at least two active joints plus daily spiking fever above 38°C within the week prior to randomization. More than half of patients (15/25) responded to CAN in the first 15-day period, with all responders achieving not just the primary outcome of adapted ACR Pedi 30 but also the secondary outcome of adapted ACR Pedi 50. 4/15 achieved inactive disease status by day 15. Although over the course of every 2-week follow-up, the majority of responders relapsed, most could recapture clinical response with re-dosing. There were two patients who sustained disease inactivity off CAN, one patient after day 15, and the other after one re-dose for relapse. As mentioned above, non-responders during stage 1 had the option of re-enrolling at a higher dose. Authors also informed preliminaries safety data for CAN use in s-JIA patients with active systemic features. Of the 23 patients enrolled, 5 received CAN for more than 1 year and 7 for more than 2 years. During the study period, there were no deaths or MAS events. One participant dies more than 2 years after the last dose of CAN by sepsis. The investigators monitored for any AE which were typically mild. No patients withdrew from the study due to AE, and the most reported events were cough, pyrexia, and gastrointestinal manifestations including abdominal pain, vomiting, and diarrhea. There were two SAE thought to be related to CAN: an infection with EBV as well as a gastroenteritis-type illness with hematoma and prolonged activated partial thromboplastin time.

Ruperto N, et al.^41^ also published the results of the pivotal phase III trials. They demonstrated the efficacy and safety of CAN in s-JIA patients with active systemic features. In trial 1, the authors randomly patients, 2 to 19 years of age, with s-JIA and active systemic features, in a double-blind fashion, to a single SC dose of CAN (4 mg per kilogram) or placebo. In trial 2, after 32 weeks of open-label treatment with CAN, patients who had a response and underwent GC tapering were randomly assigned to continued treatment with CAN or to placebo. At day 15 in trial 1, more patients in the CAN group had an adapted JIA ACR 30 response (84% vs. 10% in the placebo group; p<0.001). In trial 2, among the 100 patients (of 177 in the open-label phase) who underwent randomization in the withdrawal phase, the risk of flare was lower among patients who continued to receive CAN than among those who were switched to placebo (74% of patients in the CAN group had no flare vs. 25% in the placebo group, HR 0.36; p=0.003). The average GC dose was reduced, and GC were discontinued in 33%. The MAS occurred in 7 patients; infections were more frequent with CAN than with placebo. The investigators determined that CAN conferred a 64% RR reduction for s-JIA flare. At the end of the withdrawal phase, 62% of CAN patients had inactive disease compared to 34% of placebo patients. At the start of trial 2, 72% were on concomitant GC therapy. Over the course of this trial, 57 patients were able to taper GC (p<0.001), and 42 of the 128 glucocorticoid-treated patients (33%) were able to discontinue therapy. Regarding safety, between trials 1 and 2, a total of 190 patients were monitored for AE. The most common AE reported were infections, with similar rates between treatment groups. There were no cases of opportunistic infections or tuberculosis. In the withdrawal phase of trial 2, six placebo group patients discontinued the study. Of these six patients, three withdrew due to events classified as non-serious (vomiting, rash, uveitis) while three withdrew due to SAE (s-JIA flare, urosepsis with MAS, measles and pneumonia).

Feist E, et al.^[[47]](#endnote-48)^ described the safety and efficacy of CAN, data pooled from 4 s-JIA studies. Data from patients who had received at least one dose of 4 mg/kg CAN in 3 studies (studies 1, 2 and 3) were pooled and included in the analysis for efficacy outcomes. The efficacy parameters across the 3 age groups were largely comparable. ACR Pedi responses to CAN treatment were rapid in all age groups; at day 15, at least 50% of patients in each age group had ACR ≥70 responses. The response seen at day 15 was maintained or improved over time until at day 85. Similar results were observed for JIA ACR responses. At day 15, at least 50% of patients from each age group (51.4%, 58.9% and 65.5 % for children, young adolescents and older adolescents and young adults, respectively) had ACR ≥70 responses and showed improvements (57.9%, 66.7% and 66.7% for children, young adolescents and older adolescents and young adults, respectively) at day 85. By day 15, absence of intermittent fever was observed in 85.8-96.4% of patients, with minimal difference between age groups. The median CRP levels rapidly decreased from baseline to day 15 and remained low at day 85 in all age groups. In total, 324 patients were analysed for safety including 233 children, 60 young adolescents and 31 older adolescents and young adults. One patient (13-year-old male) from the young adolescents group died because of pulmonary hypertension that was associated with MAS. The two most common SAEs in all 3 age groups were flare of s-JIA and MAS.

Brunner H, et al.^[[48]](#endnote-49)^ studied in an open-label active treatment newly-recruited CAN-naïve s-JIA patients, with and without fever at enrollment. 64.2% of the patients were previously exposed to bDMARDs. JIA-ACR 50/70/90/100 responses at day 15 since CAN initiation were achieved by 60.0%, 48.6%, 37.1%, and 24.3% patients in the fever group. Similar levels of response occurred in the no-fever group. Response rates increased until month 6 post-CAN initiation and were sustained for the initial 1.5 years of the study in both subgroups. The apparent decline in response rates after 1.5 years was due to patients’ loss to follow-up. The exposure-adjusted incidence rate of SAE over the duration of the study was 56/100 PY. Higher incidence rates of SAE (95.7/100 PY) was observed during the initial 6 months of the study, and then this rate decreased to 59.0/100 PY by month 24. Rates for serious infection remained stable over time (month 6: 14.73/100 PY; month 12: 14.83/100 PY; month 24: 12.28/100 PY).

Nishimura K, et al.^[[49]](#endnote-50)^ reported data of CAN treatment in s-JIA patients. Efficacy: 84.2% (16/19) and 94.7% (18/19) of patients achieving ACR Pedi 30 at weeks 2 and 4, respectively. Regarding corticosteroid tapering: of the 19 patients, 73.7% successfully tapered their GC dose, with 10.5% patients being GC-free at week 28. 14.3% patients who successfully tapered their GC dose at week 28 received IV corticosteroid after week 8 due to AE (MAS suspected and s-JIA flare). These 2 patients achieved ACR Pedi 90/100 response by week 2 and their oral GC doses were tapered after week 8. Overall, 26.3% patients were not able to taper GC, including 1 (5.3%) patient who discontinued the study before week 8. Response to treatment over time and inactive disease: more than 80.0% of patients achieved ACR Pedi 30/70 at week 2 and ACR Pedi 90 by week 4. By week 28, 100.0% (16/16), 100.0%, 100.0%, and 56.0% of patients achieved ACR Pedi 30/70/90/100, respectively. These responses were maintained or further increased up to week 48. Inactive disease was achieved in 31.6% (6/19) and 63.2% patients at weeks 2 and 4, respectively, which further increased to 75.0% (12/16) at week 48. The flare criteria were met for 3 patients during the 48 weeks of treatment. Two patients had flares before week 8, and 1 patient experienced flare after the initiation of corticosteroid tapering. No patients had flare after week 28 to week 48. The median duration of exposure to CAN was 337 days and ~65.0% of patients received treatment for ≥48 weeks. Overall, 8 (42.1%) patients experienced 16 SAE during the study. Two (10.5%) patients discontinued treatment due to a SAE. No malignancies or anaphylactic reactions were reported. No deaths occurred during the 48-week study.

**Table.**

| **Author, year,**  **Study methodology**  **Duration** | **Population Description** | **Treatment** | **Results** |
| --- | --- | --- | --- |
| Horneff 2015 ^47^  Opnel label single arm  Duration: 94 weeks | Patients aged 2-20 years with s-JIA with and without s-JIA associated fever | CAN 4 mg/kg every 4 weeks  n=122: 53 with fever and 69 without fever | **Week 4:** with fever: ACR/JIA 30: 75%; without fever: ACR/JIA 30: 75%  **Week 12:** with fever: aACR/JIA 30: 90%; without fever: ACR/JIA 30: 90%  **Week 21:** with fever: inactive disease: 60%; without fever: inactive disease: 70%  **Baseline**  Median JADAS score: 21.5  Moderate (JADAS >3.8 and ≤10.5): 8 (7.5%)  High disease activity (JADAS >10.5): 99 (92.5%)  **Day 15,**  Median JADAS: 6.8 and 1.5 at the last assessment  Inactive disease (JADAS ≤ 1): 53 (48%)  Low (JADAS >1 and ≤3.8): 10 (9%)  Moderate (JADAS >3.8 and ≤10.5): 14 (13%)  High disease activity (JADAS >10.5): 31 (28%)    AE: Infection (0.56 infections/100 patient-days), typically involving upper respiratory tract. Fifteen patients discontinued due to an AE and 40 had >1 SAE (mostly infections, MAS, or flare) and no deaths. Eight cases of MAS (0.013 events/100 patient days) were reported. |
| Ruperto 2012 ^48^  Phase II trial:  Multicenter open-label study  Duration: 56 weeks | s-JIA >4 years of age, had fever, and were receiving ≤0.4 mg/kg/day of GC | Single SC CAN, 0.5-9 mg/kg of body weight, and were redosed upon relapse  n=25 | **Day 15:**  ACR30: 15/25 (60%) / ACR50: 15/25 (60%) / ACR90: 5/25 (20%) / Inactive Disease: 4/25  **1 year:**  Mainteined response: 11/13 / Steroid sparing: 8/11  Discontinued steroids: 4/11 / Non-responders: 4/15  **Any AE:** 22/23 (96%), cough, abdominal pain, pyrexia, vomiting, diarrhea, gastroenteritis  **SAE** were reported in 2 subjects: one had an Epstein-Barr virus infection during stage 1, and the other had a hematoma, prolonged activated partial thromboplastin time, gastroenteritis, and syncope during stage. |
| Ruperto 2012 ^49^  Phase III trial:  Part 1 randomized, doubleblind, placebo-controlled study.  Paart 2 consisted of a two-part withdrawal study.  .  Duration: 2 years | 2-19 years active s-JIA.  Part 2 second phase: Patients were eligible if they had sustained at least an adapted ACR Pedi 30 response and either were not on GC or had successfully tapered and were on a stable dose. | CAN (4 mg/kg every 4 weeks [q4w] subcutaneously) | Part 1:  **ACR Pedi 30:** 36/43 CAN-treated patients (84%) vs 4/41 placebo-treated patients (10%).  **Inactive disease status by day 15:** 13/43 (33%). It was sustained at 29-day period and with 81% (35/43) at the end of the study.  Part 2: the open-label phase, n=177 patients  **ACR Pedi 30:** 135/177 (77%)  **ACR Pedi 50:** 135/177 (77%)  **Inactive disease status by day 15:** 30%  100/177 (56%) sustained ACR pedi30 at the end of the study.  Over the open-label phase: were able to wean steroids: 57/128 patients. Of these patients, 42 (33%) ultimately discontinued steroids.  **AE**: In Part 2 withdrawal phase, CAN showed 64% RR reduction for flare. Two deaths, both in placebo patients. Seven reported MAS events, five probable, two possible. |
|  |  |  |  |
| Brunner 2020 ^51^  Open label single arm  Duration: 2 years | Active s-JIA patients (2- 20 years) | CAN (4mg/kg every 4 weeks [q4w] subcutaneously).  n=123.  Fever=70. No-fever=52. | **Day 15: Fever group**  JIA-ACR 50: 60% / JIA-ACR 70: 48.6% / JIA-ACR 90: 37.1% / JIA ACR 100: 24.3%  **Day 15: no-fever group:**  JIA-ACR 50: 67.3% / JIA-ACR 70: 48.1% / JIA-ACR 90: 34.6% / JIA ACR 100: 19.2%  **Month 6: Fever group:**  CRJADAS/CRACR: 17 (24.3%) / 26 (37.1%)  **Month 6: no-fever group:**  CRJADAS/CRACR: 9 (17.3%) /12 (23.1%)  **AE:** Fever-group mainly infections 10 (14.3%), blood and lymphatic system disorders 9 (12.9%) and musculoskeletal and connective tissue disorders 10 (14.3%). No-fever group mainly musculoskeletal and connective tissue disorders 7 (13.5%), infections 3 (5.8%), blood and lymphatic system disorders 4 (7.7%). |
| Nishimura 2021 ^52^  Open label single arm  Duration: 48 weeks | Active s-JIA patients (2- 20 years) | CAN (4mg/kg every 4 weeks [q4w] subcutaneously)  n=19  15 (78.9%) had previously used TOC. | All patients achieved ACR Pedi 30 at week 8.  73.7% (14/19) successfully tapered GC at week 28.  At week 48, ACR Pedi 50/70/90/100 responses were achieved by 100%/100%/87.5%/68.8% of patients.  **AE**: the most common AE were infections (271.6 PY), 42.1% (8/19) patients had SAE. Two potential cases of MAS were identified. No deaths were reported. |

# INTERFERON GAMMA (INF) - Recombinant interferon (IFN) gamma

**Non comparative observational or single arms studies included.**

Coto C, et al.^[[50]](#endnote-51)^ evaluated interferon gamma in patients with diagnosis of active JIA and were resistant or had severe adverse reactions to previous treatments with NSAID, DMARD, steroids, and cytostatic drugs. Eight of the 10 patients had significant clinical response, 7 of them complete. They did not have signs of the disease. Steroid therapy could be suspended after 2 to 8 months of IFN treatment in 7 of the 8 children who were taking steroids. Two patients did not respond. Relapses occurred in 4 patients, one of them with severe systemic symptoms that required intensive care, one month after abandoning the IFN treatment. The other 3 patients had mild joint inflammation. Intercurrent infections occurred in most patients, but only in one case were considered severe.

**Table 3.**

| **Author, year,**  **Study methodology**  **Duration** | **Population Description** | **Treatment** | **Results** |
| --- | --- | --- | --- |
| Coto 1998 ^53^  Observational study  Duration: 2 years | Children aged 6 to 15 years with active s-JIA (n=6) and were resistant or had severe adverse reactions to previous treatments with NSAID, DMARD, steroids, and cytostatic drugs. | IFN: The dose and schedule of IFN were 50000 IU–kg of weight (up to 1*10^6^ IU), intramuscularly, daily for 4 weeks, then 3 times per week for 3 months and 2 times per week up to 2 years | 3/6 patients had complete clinical response. One treatment suspended because of pregnancy. One abandoned after 10 months.  **AE:** 3/6 pneumonia, 2/6 tonsillitis, 1/6 retropharyngeal abscess, 3/6 low leukocyte counts (<12.0*10^9^ cells/l). |

# RILONACEPT (RILO)

Lovell D, et al.^[[51]](#endnote-52)^ evaluated rilonacept in s-JIA patients. Efficacy and safety of rilonacept were evaluated during 23 months of open-label treatment (3 phases) after a 4-week, double-blind, placebo-controlled phase. Following double-blind treatment with 2.2 mg/kg or 4.4 mg/kg of rilonacept, patients were eligible to receive open-label treatment at their prior dose, with adjustments. 25 patients were screened and 24 were enrolled in the double-blind phase. The study population was predominantly white females, with a median age of 14.0 years. No significant differences in efficacy were observed between the rilonacept- and placebo-treated patients during the double-blind phase, but fever and rash completely resolved by month 3 in all patients during the open-label treatment period and did not recur. ACR Pedi 30, 50, and 70 response rates at 3 months from the start of the study were 78.3%, 60.9%, and 34.8%, respectively; these responses were generally maintained over the study duration. No serious treatment-related AE were observed.

Ilowite N, et al.^[[52]](#endnote-53)^ conducted an initial 4-week double-blind placebo phase was incorporated into a 24-week randomized multicenter design, followed by an open-label phase. Seventy-one children who had active arthritis in >2 joints were randomized (1:1) to the 2 arms of the study. Patients in the rilonacept arm received rilonacept (loading dose 4.4 mg/kg followed by 2.2 mg/kg weekly, subcutaneously) beginning on day 0. Patients in the placebo arm received placebo for 4 weeks followed by a loading dose of rilonacept at week 4 followed by weekly maintenance doses. The primary end point, time to response as defined by time to achieve the composite end point of an ACR Pedi 30 response, absence of fever, and GC taper (for patients receiving GC), was shorter in the rilonacept arm (median 4 weeks; 25th, 75th percentiles 2 weeks, 10 weeks) than in the placebo arm (median 8 weeks; 25th, 75th percentiles 6 weeks). By week 12, 27 (77%) of 35 patients receiving rilonacept continuously from onset of the trial and 20 (59%) of 34 patients receiving placebo for the initial 4 weeks met the primary end point of response. The secondary end point analysis showed that 20 of 35 patients in the rilonacept arm (57%) and 9 of 33 patients in the placebo arm (27%) had a response at week 4. The incidence of infection in the rilonacept group was not higher than that in the placebo group during either phase. The most common SAE was s-JIA flare. The single arm of both studies is described in the table below.

**Table 4.**

| **Author, year,**  **Study methodology**  **Duration** | **Population Description** | **Treatment** | **Results** |
| --- | --- | --- | --- |
| Lovell 2013 ^54^  Phase I: 4 weeks double blinded, randomised, placebo-controled study.  Phase II: open label extension (all active drug) up to 24 months.  Duration:  24 months | Patients aged 4-20 years with active s-JIA. | **Phase I (4 weeks):**  Cohort I: Rilonacept 2.2 mg/kg SC on days 0, 3, 7, 14, and 21.  Cohort 2: Rilonacept 4.4 mg/kg SC on days 0, 3, 7, 14, and 21.  Control: Placebo SC on days 0, 3, 7, 14, and 21.  **Phase II (24 months):**  Rilonacept 2.2 or 4.4 mg/kg/week based on the investigator’s clinical judgment. | **Phase II**  Fever (%): 24 months: 0/23 / Rash (%): 24 months: 0/23  **ACR 30, %:** 3 months: 78.3% / 12 months: 91.3% / 24 months: 69.9%  **ACR 50, %:** 3 months: 60.9% / 12 months: 87% / 24 months: 69.9%  **ACR 70, %:** 3 months: 34.8% / 6 months: 42.9% / 12 months: 82.6% / 24 months: 56.9%  **PGA, median reduction (%):** 3 months: 40%  **Joints with LOM, median reduction (%):** 3 months: 45.3%  For most of the ACR Pediatric core criteria over 3 months, the median reduction was at least 80%, with the hsCRP level showing the greatest reduction (median reduction 96%).  *Safety*  **Any AE**, n (%): 23 (100)  **SAE**, n (%): 3 (13)  Discontinued due to AE, n (%): 3 (13)  Any treatment-related AE, n (%): 16 (69.6)  Death n (%): 0  General disorders and administration site conditions, n (%): 17 (73.9)  **Infections**, n (%): 16 (69.5); **Serious infections**, n (%): 0 |
| Ilowite 2014 ^55^  **Phase I:** 4 week double blinded, randomised, placebo-controled study.  **Phase II:** open label extension (all active drug) up to 24 week.  **Phase III:** open lable extension from 24 week to 21 months.  Duration: 21 months | s-JIA according to ILAR criteria. | **Phase I:**  Intervention: Day 0: rilonacept 4.4 mg/kg  weekly dose: 2.2 mg/kg/day.  Control: Placebo day 0-7-14-21.  **Phase II and III:** Rilonacept 2.2 mg/kg weekly | **Phase II,** 24 weeks  Rash, n (%): 4/57 (7%)  **ACR 30**, n (%): 45/55 (82%)  **ACR 50**, n (%): 43/55 (78%)  **ACR 70**, n (%): 35/55 (64%)  **Inactive disease**, n (%): 11/55 (20%)  N° of joints with active arthritis, median (IQR): 1 (0-7)  N° of joints with limited ROM, median (IQR): 1 (0-7)  Physician’s global assessment of disease, median (IQR): 6 (1-33)  Parent’s global assessment, median (IQR): 7(1-29)  **CHAQ**, median (IQR): 0.13 (0-1)  **CRP** (mg/dL), median (IQR): 0.4 (0.4-1.95)  **ESR** (mm/h), median (IQR): 0.5 (6-22)  Patients with ≥ 1 **AE**, n (%): 53/68 (81%)  Patients with ≥ 1 **SAE**, n (%): 4/68 (6%)  Patients with ≥ 1 **infection**, n (%): 36/68 (53%)  **Phase III (n=40)**  Patients with ≥ 1 **AE**, n (%): 21 months: 28/40 (70%)  Patients with ≥ 1 **SAE**, n (%): 21 months: 6/40 (15%)  Patients with ≥ 1 **infection**, n (%): 21 months: 14/40 (35%) |

IQR: interquartile range; AE: adverse event; SAE: serious adverse event; ESR: erythrocyte sedimentation rate. CRP: C-reactive protein. ROM: range of motion. PGA: parents’ global assessment of well-being; CHAQ: Childhood Health Assessment Questionnaire.

# CYCLOSPORINE (CsA)

**Non comparative observational or single arms studies included.**

Gerloni V, et al.^[[53]](#endnote-54)^ studied 34 patients with s-JIA and chronic anterior uveitis associated with juvenile chronic arthritis. The average initial dose for the population was 4.2 mg/kg per day, with a maximum dose of 5.0 mg/kg per day an average maintenance dose of 3.6 mg/kg per day. The efficacy of treatment was mainly evident in terms of control of fever and reduction of steroid therapy. The benefits respect to arthritis, laboratory parameters and uveitis seemed to be less clear-cut. Side-effects were frequent but usually mild or reversible. Sixty-six percent of the study of the population withdrew from therapy because of inefficacy or side-effects. Eight systemic patients withdrew from therapy owing to complete remission.

Pal P, et al.^[[54]](#endnote-55)^ studied in a prospective observational study 82 children with steroid dependent/refractory (n=15) s-JIA. CsA was used in 15 patients over a time period of 5 years and the average starting dose was 3.1 mg/kg/d (range 1.9 to 4) and showed a favorable response with significant steroid sparing effect and minimal toxicity. Of these, 13 responded to CsA and 2 did not respond. Of the 13 responders, 11 became afebrile within 2 weeks of initiation of CsA, the remaining 2 within a month. Two patients relapsed at 10 and 14 months while on CsA but could be managed with a short course of steroid.

| **Author, year,**  **Study methodology**  **Duration** | **Population Description** | **Treatment** | **Results** |
| --- | --- | --- | --- |
| Gerloni 2001 ^56^  Prospective Observational Study  Duration: 10 years | JIA patients according to Oslo criteria with cDMARDs failure and  active disease (fever, arthritis, corticoid dependency, uveitis) | Cyclosporin, starting dose 4.2 mg/kg/day  n=41; s-JIA=36 | **Defervescence**, n (%): 13/25 (52%)  **Improvement in joint symptoms**, n (%): 12 months: 12/28 (43%)  **Physician´s global evaluation**, n (%): Poor: 16/29 (55%); Moderate: 6/29 (21%); Good: 7/29 (24%)  **ESR improved,** n (%): 12 months: 10/28 (36%)  **Side effects,** n (%): 9/34 (26%)  **Inefficacy,** n (%): 8/34 (23%)  **Disease remission,** n (%): 8/34 (23%)  **Disease flare-up,** n (%): 7/34 (20%) |
| Pal 2019 ^57^  Prospective Observational  Duration: 12 months | s-JIA patients according to 1995 ILAR criteria with repeated flares (3 or more times) or corticoid dependency. | Cyclosporin, starting dose was 3.1 mg/kg/d (range 1.9 to 4).  n= 15 | **Months 12**  **Response to therapy**, n(%): 13/15 (86.6%)  **Defervescence**, n (%): 13/13 (100%)  **Improvement in joint symptoms**, n (%): 5/5 (100%)  **70% reduction of ESR and CRP**, n (%): 13/13 (100%)  **Decrement of the dose of steroid by 50%**, n (%): 13/13 (100%) |

ESR: erythrocyte sedimentation rate. CRP: c-reactive protein.

# STEM CELLS

**Non comparative observational or single arms studies included**.

Brinkman D, et al.^[[55]](#endnote-56)^ reported safety and efficacy of intensive immunosuppression followed by T cell-depleted autologous hematopoietic stem cell transplantation (ASCT) for induction of disease remission in children with refractory progressive s-JIA. Twenty-two patients with progressive refractory JIA were followed up over a median period of 80 months after pretreatment with intensive immunosuppression followed by ASCT in a multicenter, prospective, phase II clinical trial. Hematopoietic stem cells were harvested from the patients’ bone marrow, depleted of T cells, and kept frozen until used for ASCT. Pretreatment of patients consisted of a combination of antithymocyte globulin, cyclophosphamide, and low dose total body irradiation. Patients were followed up for ASCT-related complications, recovery of hematologic and immune system parameters, and disease outcomes. The results showed that reconstitution of hematologic values to normal range was rapid. Recovery of immune system parameters, especially normalization of CD4, CD45RA naive T cells, was delayed, occurring at >6 months after ASCT. The prolonged period of immune deficiency resulted in a large number of viral infections and may have contributed to the development of MAS, leading to death, in 2 patients. After ASCT, 8 of the 20 evaluable patients reached complete clinical remission of their JIA, 7 were partial responders, and 5 experienced a relapse of their disease (occurring 7 years after ASCT in 1 patient). Later during follow-up, 2 of the patients whose disease relapsed died from infections that developed after restarting immunosuppressive medication. The probability of overall survival of the whole population of 22 patients with JIA at 5 years was 82% and the probability of disease-free survival, censored for relapse and death as events, was 36%. Two children died from MAS, which occurred at 18 days post-ASCT in 1 patient and 4 months post ASCT in the other patient, and the deaths were associated with Staphylococcus epidermidis bacteremia and Epstein-Barr virus reactivation.

Silva J, et al.^^[[56]](#endnote-57)^^ reported 16 patients in 5 transplant centers between 2007 and 2016: 11 children with s-JIA and 5 with rheumatoid factor-negative p-JIA; all were either refractory to standard therapy, had developed secondary MAS poorly responsive to treatment, or had failed autologous HSCT. All children received reduced toxicity fludarabine-based conditioning regimens and serotherapy with alemtuzumab. Fourteen of 16 patients are alive with a median follow-up of 29 months (range, 2.8-96 months). All patients had hematological recovery. Three patients had grade II-IV acute graft-versus-host disease. The incidence of viral infections after HSCT was high, likely due to the use of alemtuzumab in already heavily immunosuppressed patients. All patients had significant improvement of arthritis, resolution of MAS, and improved quality of life early following allo-HSCT; most importantly, 11 children achieved complete drug-free remission at the last follow-up. Allo-HSCT using alemtuzumab and reduced toxicity conditioning is a promising therapeutic option for patients with JIA refractory to conventional therapy and/or complicated by MAS.

Swart J, et al.^[[57]](#endnote-58)^ compared the total number of AE before and after mesenchymal stromal cell infusion in refractory JIA and to evaluate its effectiveness. It was a single-centre proof of mechanism Phase Ib, open label intervention study in JIA patients previously failing all biologicals registered for their diagnosis. Six patients received 2 million/kg IV infusions of allogeneic bone marrow derived MSC. In case of ACR Pedi 30 response but subsequent loss of response one and maximal two repeated infusions were allowed. Six JIA patients with 9.2 years median disease duration, still active arthritis and damage were included. All had failed MTX, GCs and median five different biologicals. MSC were administered twice in three patients. No acute infusion reactions were observed and a lower post-treatment than pre-treatment incidence in AE was found. The one s-JIA patient had again an evolving MAS, 9 weeks after TOC discontinuation and 7 weeks post-MSC infusion. Significant decreases were found 8 weeks after one MSC infusion in VAS well-being (75-56), JADAS-71 (24.5-11.0) and cJADAS10 (18.0-10.6). Authors concluded that MSC infusions in six refractory JIA patients were safe, although in s-JIA stopping the ‘failing’ biologic treatment carries a risk of a MAS flare, as the drug might still suppress the systemic features.

**Table 5**

| **Author, year,**  **Study methodology**  **Duration** | **Population Description** | **Treatment** | **Results** |
| --- | --- | --- | --- |
| Brinkman 2007 ^58^  Multicenter, prospective phase II clinical trial  Duration: 1997-2001 | s-JIA based on ACR criteria with active disease. Progressive refractory p-JIA | Intensive immunosuppression followed by T cell– depleted autologous hematopoietic stem cell transplantation (ASCT)  n= 22; 18 patients had s-JIA and 4 had p-JIA. | **Clinical remission after ASCT:** 8 patients (2 p-JIA and 6 s-JIA)  **Partial responders:** 7 patients (2 p-JIA and 5 s-JIA)  **Relapse:** 5 patients  **Deaths:** 2 children died from MAS after ASCT  Probability of survival at 5 years: 82%  Probability of disease-free survival: 36% |
| Silva 2018 ^59^  Retrospective  Duration: 2007-2016 | s-JIA and p-JIA active arthritis refractory to standard therapy, had developed secondary MAS poorly responsive to treatment, or had failed autologous HSCT. | Fludarabine-based conditioning regimens and serotherapy with alemtuzumab. Allogeneic hematopoietic stem cell transplantation  n= 11 patients with s-JIA and 5 p-JIA. | **Month 29**  Hematological recovery: 16/16  Grade II-IV acute graft-versus-host disease: 3/16  Improvement: 16/16  Drug-free remission at last follow up: 11/16 (4 p-JIA and 7 s-JIA)  Continued with treatment at last follow up: 5/16 (1 p-JIA and 4 s-JIA)  Alive: 14/16  Deaths: 2/16 (1 s-JIA and 1 p-JIA) |
| Swart 2019 ^60^  Single-centre proof of mechanism Phase Ib, open label intervention study  Duration: 1997-2001 | s-JIA, p-JIA and extended o-JIA active arthritis and damaged who had failed MTX, GC and median five different biologicals. | 2 million/kg IV infusions of allogeneic bone marrow derived MSC  n=6 (3 p-JIA, 2 o-JIA, 1 s-JIA) | At week 8, decreases in:  VAS well-being: 75-56  JADAS-71: 24.5-11.0  cJADAS10: 18.0-10.6  **AE:** No acute infusion reactions were observed and a lower post-treatment than pre-treatment incidence in AE was found (p=0.36) |

## MACROPHAGE ACTIVATION SYNDROME (MAS)

The evidence of MAS consists in six retrospective studies and the report of ten clinical cases studies, with a total of 520 patients. The drugs studied were Anakinra, Cyclosporine, Etoposide, Steroids, intravenous immunoglobulin and Tocilizumab.

The description of each study is summarized below according to each drug.

# ANAKINRA

| **Author, year,**  **Study methodology** | **Population / Treatment** | **Clinical Cases or Results** |
| --- | --- | --- |
| Bruck 2012^[[58]](#endnote-59)^  2 clinical case report | Two patients met the criteria for the diagnosis of s-JIA as defined by the ILAR and also for the diagnosis of MAS according to the criteria suggested by Ravelli and Magni-Manzoni.  Treatment:  **ANA** 2 mg/kg/day | Case 1: 8-years-old boy with new s-JIA diagnosis developed MAS 10 days after discharged. He received previous treatment with methylprednisolone pulse (30 mg/kg per day/3 days) and treatment with indomethacin and prednisolone (2 mg/kg per day). When he had reached 1 mg/kg/day of prednisone he presented a dramatic increase in in liver transaminases, ferritin, and D-dimers, accompanied by a paradoxical drop in thrombocytes. Physical examination revealed new hepatomegaly. Another 3-day IV methylprednisolone pulse (30 mg/kg per day) was given, followed by oral prednisolone at 2 mg/kg per day. MAS was diagnosed, and treatment with ANA 2 mg/kg per day SC was initiated the same day. Fevers resolved, the patient’s sense of well-being returned, and all laboratory values normalized within 20 days. The prednisolone was tapered over 6 weeks to a dose of 10 mg/day, after another 4 weeks to 5 mg/day and discontinued after another 2.5 months. The indomethacin was discontinued after 6 months and the ANA after 15 months. Eight months later, the patient remains in full remission without medications.  Case 2: 12-year-old white girl who presented with high spiking fevers, a strongly erythematous, easily blanchable rash on buttocks and extremities that worsened with fever spikes, and a greatly reduced ability to move because of severe muscle and joint pains. A bone marrow aspirate showed no evidence of malignancy or hemophagocytosis. The patient defervesced and regained near-normal joint mobility under treatment with ibuprofen and prednisolone (2 mg/kg per day). However, after 2 weeks, when the prednisolone was reduced slightly (1.7 mg/kg per day), the patient developed recurrent fevers and new sonographically confirmed hepatosplenomegaly and cervical and abdominal lymphadenopathy. Laboratory studies revealed a pancytopenia with complete agranulocytosis; highly elevated serum ferritin, D-dimer, lactate dehydrogenase, aspartate aminotransferase, alanine aminotransferase, and F-glutamyl transferase levels; and a paradoxical drop in CRP. A second bone marrow aspirate showed widespread hemophagocytosis. She received treatment with dexamethasone (10 mg/m^2^ per day) for 3 days but did not affect fever or agranulocytosis. After review of the patient’s history, the diagnosis was revised to s-JIA complicated by MAS, and ANA (2 mg/kg per day SC) was started. The patient defervesced within 48 hours and the granulocyte count normalized within 14 days as did nearly all other laboratory indices despite a reduction of dexamethasone to 5 mg/m^2^ per day on day 10. The patient was discharged home on day 14. |
| Phadke 2021 ^[[59]](#endnote-60)^  Retrospective control | Patients with secondary MAS according to Ravelli criteria.  19 patients (10 s-JIA)  Treatment: **ANA** 2 mg/kg and titrated up to a maximum of 100 mg IV every 12 h. | Median age: 13 years  Median therapy duration: 10 days  Initial dose of IV ANA: ranged from 1.7 to 10 mg/kg/day and the maximum dose of IV ANA ranged from 4.2–15.4 mg/kg/day.  *Safety*  Increase transaminases: 1/19  Dead: 5/19 patients died from their underlying disease or complications. |
| Sönmez 2017 ^[[60]](#endnote-61)^  Retrospective cohort  Duration: 2015-2017 | Pediatric MAS patients, who were followed up and treated with ANA (anti-IL1).  All MAS patients secondary to s-JIA or AIDs were treated with ANA after January 2015, no patient was left out. In 2015, Ravelli’s criteria was not published; thus, the diagnosis of MAS was based on expert opinion.  15 patients (13 s-JIA patients)  Treatment:  **ANA** (2 mg/kg/day) was initiated with a median 1 day after hospitalization. ANA dose was increased to 4–6 mg/kg/day in two patients, because there was no response in the ferritin and thrombocyte counts in 12 h.  All patients received pulse methylprednisolone and continued with oral prednisolone.  Overall, seven MAS episodes, patients received steroids and ANA with/without IVIG, and six MAS episodes, patients received steroids + ANA + cyclosporine A with/without IVIG. In the other six MAS episode, patients received plasmapheresis + steroids + ANA + cyclosporine with/without IVIG. Three of the latter group also received a single low dose of etoposide. | 19 MAS episodes in 15 patients  Among 19 MAS episodes all (100%) had hyperferritinemia and thrombocytopenia below 180,000/ mm^3^, 14 (73.6%) had hypofibrinogenemia, 13 (68.4%) had transaminasemia, and 12 (63.1%) had hypertriglyceridemia. Hemophagocytosis in bone marrow aspiration was detected in all of 15 patients.  13 patients achieved clinically and laboratory remission with steroids + ANA +/− cyclosporine A with/without IVIG.  2 patients developed recurrent MAS.  ANA treatment was stopped in five patients at a median of 13 months after being discharged from the hospital. The other eight patients are now clinically and laboratory remission with alternate day anakinra (2 mg/kg).  None of the patients experienced severe injection site reactions. |

# CYCLOSPORINE

| **Author, year,**  **Study methodology** | **Population / Treatment** | **Clinical Cases or Results** |
| --- | --- | --- |
| Kounami 2005 ^[[61]](#endnote-62)^  4 clinical cases report | 4 patients with MAS (according Ravelli criteria) associated with s-JIA.  Treatment: Immunosuppressive treatment  Steroids + Cyclosporine | Patient 1:  Sex: Female  Age at onset of MAS: 4 years.  Previous treatment: mizorbine, tolmetin sodium.  Delay to diagnosis of MAS days: 4  Neurological symptoms: No  ICU-care: Yes.  MAS therapy: MP pulse (30 mg/kg for 3 days), IG (400 mg/kg for 3 days), Cyclosporine 2.5 mg/kg/day  Clinical response: complete.  Patient 2:  Sex: Male  Age at onset of MAS: 11 years.  Previous treatment: prednisolone, aspirin.  Delay to diagnosis of MAS days: 4  Neurological symptoms: No  ICU-care: Yes.  MAS therapy: Prednisolone, Cyclosporine 3 mg/kg/day IV  Clinical response: complete.  Patient 3:  Sex: female  Age at onset of MAS: 14 years.  Previous treatment: none.  Delay to diagnosis of MAS days: 10  Neurological symptoms: No  ICU-care: Yes.  MAS therapy: Cyclosporine 3 mg/kg/day IV  Clinical response: complete.  Patient 4:  Sex: female  Age at onset of MAS: 3 years.  Previous treatment: aspirin.  Delay to diagnosis of MAS days: 4  Neurological symptoms: Yes  ICU-care: Yes.  MAS therapy: Cyclosporine 3 mg/kg/day IV  Clinical response: complete. |
| Bennet 2012 ^[[62]](#endnote-63)^  Retrospective cohort | They identified children who were treated at a PHIS hospital and had International Classification of Diseases, Ninth Revision, Clinical Modification (ICD-9-CM) discharge diagnosis codes for MAS and either SLE or JIA from October 1, 2006 to September 30, 2010.  121 patients (102 JIA)  Treatment:  Immunosuppressive treatment in JIA patients:  Any corticoids: 93%  Cyclosporine: 42%  IVIG: 18%  IL-1 blockade: 15% | A median of 4 patients (range 1–18, interquartile range [IQR] 2–5) per hospital were treated at 28  hospitals over 48 calendar months.  Of the 102 children with JIA, 90 (88%) had a diagnosis code for systemic JIA, 8 (8%) for polyarticular JIA, 3 (3%) for pauciarticular JIA and 1 (1%) for monoarticular JIA.  ICU admission was common (33% of the entire cohort). The median length of hospital stay was 8 days (IQR 5–19 days)  Mortality rate in JIA patients: 6% |
| Lin 2012 ^[[63]](#endnote-64)^  1 Clinical case report | Patients with MAS associated with s-JIA.  Treatment:  Immunosuppressive treatment  Steroids + Cyclosporine | Sex: female  Age at onset of MAS: 15 years.  Neurological symptoms: No  ICU-care: Yes  MAS therapy: MP pulse, Cyclosporine.  Clinical response: complete. She presented two episodes of MAS in adult life. |
| Minoia 2014 ^[[64]](#endnote-65)^  Retrospective cohort | s-JIA and an episode of MAS. The diagnosis of MAS had to be based on the typical clinical and laboratory picture of the syndrome, irrespective of evidence of macrophage hemophagocytosis in the bone marrow.  n=362  Treatments:  Immunosuppressive treatment:  Any corticosteroids: 97.7%  Cyclosporine: 61.2%  Biologic medications: 15.2%  Etoposide: 11.8% | Clinicals features:  Fever: 96.1%, Hepatomegaly: 70%, Splenomegaly: 57.9%, Lymphadenopathy: 51.4%,  Active arthritis: 65%, CNS involvement: 35%, Heat involvement: 25.5%, Lung involvement: 21.9%,  Hemorrhagic manifestations: 20.4%, Kidney involvement: 15.3%.  Platelet count and liver transaminase, ferritin, lactate dehydrogenase, triglyceride, and D-dimer levels were the sole laboratory biomarkers showing a percentage change of >50% between the pre-MAS visit and MAS onset.  Evidence of macrophage hemophagocytosis was found in 60% of the patients who underwent bone marrow aspiration.  MAS occurred most frequently in the setting of active underlying disease, in the absence of a specific trigger 34.9% of the patients required admission to the intensive care unit and the mortality rate was 8%. |
| Boteanu 2014 ^[[65]](#endnote-66)^  2 Clinical case report | 2 Patients with MAS (according Ravelli criteria) associated with s-JIA.  Treatment:  Immunosuppressive treatment  Steroids + Cyclosporine | Patient 1:  Sex: female  Age at onset of MAS: 9 years.  Ongoing treatment at onset of MAS: Prednisone, MTX .  Previous treatment: ANA  Neurological symptoms: No  ICU-care: Yes.  MAS therapy: prednisone (high doses), Cyclosporine.  Additional MAS treatment: -  Clinical response: complete.  Patient 2:  Sex: female  Age at onset of MAS: 14 years.  Ongoing treatment at onset of MAS: TOC  Previous treatment: ANA, ETA, steroids.  Neurological symptoms: No  ICU-care: Yes.  MAS therapy: methylprednisolone, Cyclosporine.  Clinical response: complete. |
| Aytac 2016 ^[[66]](#endnote-67)^  Retrospective cohort | A total of 34 patients with 37 episodes considered as having MAS (associated with s-JIA or SLE) and followed at Pediatric Rheumatology and Hematology Departments, Hacettepe University, Ankara, Turkey, between 2009 and 2015 were retrospectively reviewed.  The diagnosis for MAS was mainly based on expert opinion.  34 patients (28 s-JIA)  Treatment:  Immunosuppressive treatment:  All patients received pulse methylprednisolone for 3-5 days (30 mg/kg/day; max 1000 mg/day) IV and then oral prednisolone (1-2 mg/kg/day)  Cyclosporine: 74.2%  IVIG: 67.7%  ANA: 41.9%  Etoposide: 32.3% | The median age at MAS onset was 11 years.  Clinical characteristics:  Fever: 100%  Rash: 67.7%  Arthralgia/Arthritis: 54.8%  Hepatosplenomegaly: 32.3%  High C-reactive protein and hyperferritinemia were present in all MAS episodes.  The overall mortality rate was 11.8 %. |

MAS: Macrophage Activation Syndrome; IVIG: intravenous immunoglobulin. CNS: central nervous system; MP: Methylprednisolone; p.o: per oral, i.v: intravenous administration. ICU: intensive care unit.

# ETOPOSIDE

| **Author, year,**  **Study methodology** | **Population / Treatment** | **Clinical Cases or Results** |
| --- | --- | --- |
| Kounami 2005 ^[[67]](#endnote-68)^  Clinical case report | 1 Patient with MAS (according Ravelli criteria) associated with s-JIA.  Treatment:  Immunosuppressive treatment  Steroids + Etoposide. | Patient 1:  Sex: female  Age at onset of MAS: 2 years.  Previous treatment: Prednisole + aspirin + MTX  Delay to diagnosis of MAS days: 10  Neurological symptoms: Yes  ICU-care: Yes.  MAS therapy: MP pulse (30 mg/kg for 3 days), IVIG (400 mg/kg/day), etoposide (100 mg/m^2^).  Clinical response: MOF. |
| Palmblad 2021 ^[[68]](#endnote-69)^  Clinical case report | 3 Patients with MAS (according Ravelli criteria) associated with s-JIA.  Treatment:  Immunosuppressive treatment  Etoposide | Patient 1:  Sex: female  Age at onset of MAS: 9 years.  Ongoing treatment at onset of MAS: TOC, MTX .  Previous treatment: Oral steroids, MP pulses, ETA.  Neurological symptoms: Moderate  ICU-care: Yes.  First-lilne MAS therapy: MP-pulses.  Etoposide: 100 mg/m2 x 3 and 150 mg/m2 x 5.  Week on etoposide: 9  Additional MAS treatment: Oral steroids, CsA, RTX.  Clinical response: complete.  Patient 2:  Sex: female.  Age at onset of MAS: 3 years.  Ongoing treatment at onset of MAS: oral steroids, TOC, MTX.  Previous treatment: MP pulses, ETA.  Neurological symptoms: Severe  ICU-care: Yes  First-lilne MAS therapy: MP-pulses  Etoposide: 100 mg/m^2^ x 9  Week on etoposide: 8  Additional MAS treatment: Oral steroids.  Clinical response: complete.  Patient 3:  Sex: male.  Age at onset of MAS: 5 years.  Ongoing treatment at onset of MAS: oral steroids, ANA, CsA.  Previous treatment: oral steroids, IVIG, CsA, ANA, MP pulses.  Neurological symptoms: No.  ICU-care: Yes  First-lilne MAS therapy: MP-pulses, ANA 4 mg/kg.  Etoposide: 50 mg/m^2^ x 2 and 100 mg/m^2^ x 7  Week on etoposide: 10  Additional MAS treatment: Oral steroids, CsA.  Clinical response: complete. |

MAS: Macrophage Activation Syndrome; MTX: methotrexate; MP: methylprednisolone, ICU intensive care unit, CsA cyclosporine A, RTX: rituximab, IVIG: intravenous immunoglobulin. MOF: multiple organ failure.

# Steroids or IVIG

| **Author, year,**  **Study methodology** | **Population / Treatment** | **Clinical Cases or Results** |
| --- | --- | --- |
| Lin 2012 ^[[69]](#endnote-70)^  Clinical case report | Patients with MAS associated with s-JIA.  Treatment:  Immunosuppressive treatment  Oral or IV Steroids.  IVIG. | Patient 1:  Sex: female  Age at onset of MAS: 10 years.  Neurological symptoms: No  ICU-care: Yes  MAS therapy: prednisone (1-2 mg/kg/day)  Clinical response: complete. She presented an episode of MAS in adult life.  Patient 2:  Sex: female  Age at onset of MAS: 15 years.  Neurological symptoms: No  ICU-care: Yes  MAS therapy: prednisone (1-2 mg/kg/day)  Clinical response: complete. She presented an episode of MAS in adult life.  Patient 3:  Sex: female  Age at onset of MAS: 7 years.  Neurological symptoms: Yes  ICU-care: Yes  MAS therapy: IVIG, MP pulse.  Clinical response: MOF |
| Singh 2012 ^[[70]](#endnote-71)^  Clinical case report | Patients with MAS associated with s-JIA.  Treatment:  Immunosuppressive treatment  Oral or IV Steroids.  IVIG. | Patient 1:  Sex: Male  Age at onset of MAS: 5 years.  Previous treatment: MTX, naproxen  Neurological symptoms: No  ICU-care: -  MAS therapy: Oral prednisolone.  Clinical response: complete.  Patient 2:  Sex: Male  Age at onset of MAS: 2.5 years.  Previous treatment: MTX, naproxen  Neurological symptoms: Yes  ICU-care: -  MAS therapy: Oral prednisolone.  Clinical response: complete.  Patient 3:  Sex: Male  Age at onset of MAS: 10 years.  Previous treatment: -  Neurological symptoms: -  ICU-care: -  MAS therapy: MP pulse (30 mg/kg for 3 days).  Clinical response: complete.  Patient 4:  Sex: female  Age at onset of MAS: 8 years.  Previous treatment: -  Neurological symptoms: -  ICU-care: Yes.  MAS therapy: MP pulse (3 mg/kg for 3 days) IVIG (400 mg/kg for 3 days), ventilation.  Clinical response: Expired.  Patient 5:  Sex: Male  Age at onset of MAS: 10 years.  Previous treatment: -  Neurological symptoms: -  ICU-care: no.  MAS therapy: MP pulse (30 mg/kg for 3 days) IVIG (400 mg/kg for 3 days),  Clinical response: complete.  Patient 6:  Sex: Male  Age at onset of MAS: 5 years.  Previous treatment: -  Neurological symptoms: -  ICU-care: -  MAS therapy: MP pulse (30 mg/kg for 3 days).  Clinical response: complete. |
| Grom 2016 ^[[71]](#endnote-72)^  Retrospective cohort | Patients with MAS associated with s-JIA. All patients had been treated with CAN.  Two groups:  Probable MAS: Clinically consistent with MAS with histologic confirmation, laboratory features, or meeting current formal criteria for HLH or clinical and laboratory features consistent with MAS but without histologic confirmation or meeting current formal criteria for HLH.  Possible MAS: Laboratory features consistent with MAS but without clinical features, histologic confirmation, or meeting current formal criteria for HLH.  Treatment:  Probable MAS (n=21):  Corticosteroids: 21  Cyclosporine: 10  IVIG: 6  Etoposide: 2  Possible MAS (n=10):  Corticosteroids: 9 | Probable MAS:  Death, n (%): 2/21 (9.5%)  Possible MAS  Death, n (%): 0/10 |
| Nakagishi 2016 ^[[72]](#endnote-73)^  Clinical case report | Patients with MAS associated with s-JIA.  Treatment:  Immunosuppressive treatment  Dexamethasone palmitate. | Patient 1:  Sex: Female  Age at onset of MAS: 11 years.  Previous treatment: MTX, cyclosporine, TOC. MP pulse (1000 mg/day for 3 days)  Neurological symptoms: No  ICU-care: Yes.  MAS therapy: Dexamethasone 10 mg/day for 3 days.  Maintain treatment: Cyclosporine.  Clinical response: complete.  Patient 2:  Sex: Female  Age at onset of MAS: 4 years.  Previous treatment: TOC. High doses of prednisolone.  Neurological symptoms: No  ICU-care: Yes.  MAS therapy: Dexamethasone 7.5 mg/day for 3 days. Cyclosporine 1mg/kg/day.  Maintain treatment: TOC, prednisolone 5 mg/day  Clinical response: complete.  Patient 3:  Sex: Male  Age at onset of MAS: 1 year.  Previous treatment: TOC. High doses of prednisolone.  Neurological symptoms: No  ICU-care: Yes.  MAS therapy: Dexamethasone 7.5 mg/day for 3 days. Cyclosporine 1mg/kg/day.  Maintain treatment: TOC, prednisolone 5 mg/day  Clinical response: complete. |

MAS: Macrophage Activation Syndrome; MTX: methotrexate; MP: methylprednisolone; ICU: intensive care unit; CsA cyclosporine A; IVIG: intravenous immunoglobulin; MOF: multiple organ failure; TOC: tocilizumab; HLH: hemophagocytic lymphohistiocytosis.

# TOCILIZUMAB

| **Author, year,**  **Study methodology** | **Population / Treatment** | **Clinical Cases or Results** |
| --- | --- | --- |
| Rodionovskaya 2013 ^[[73]](#endnote-74)^  Clinical case report | 2 Patients with MAS associated with s-JIA.    Treatment:  Immunosuppressive treatment  Tocilizumab | Patient 1:  Sex: male  Age at onset of MAS: 10 years.  Previous treatment: MTX, steroids, CsA.  First-lilne MAS therapy: MP-pulses 15 mg/kg x 3 , CsA.  TOC: 10 mg/kg 2 week  Week on tocilizumab: 12  Clinical response: complete.  Patient 2:  Sex: female  Age at onset of MAS: 3 years.  Previous treatment: ANA 2-4 mg/kg.  First-lilne MAS therapy: steroids 1 mg/kg orally.  Neurological symptoms: yes  TOC: 12 mg/kg  Week on tocilizumab: 2  Clinical response: complete. |

MAS: Macrophage Activation Syndrome; MTX: methotrexate; MP: methylprednisolone, CsA: cyclosporine A.

## REFERENCES

1. Picco P, Gattorno M, Buoncompagni A, Pistoia V, Borrone C. 6-methylprednisolone 'mini-pulses': a new modality of glucocorticoid treatment in systemic onset juvenile chronic arthritis. Scand J Rheumatol. 1996; 25(1):24-7. doi: 10.3109/03009749609082663. [↑](#endnote-ref-2)
2. # Woo P, Southwood TR, Prieur AM, Doré CJ, Grainger J, David J, Ryder C, Hasson N, Hall A, Lemelle I. Randomized, placebo-controlled, crossover trial of low-dose oral methotrexate in children with extended oligoarticular or systemic arthritis. Arthritis Rheum. 2004; 3(8):1849-57. doi: 10.1002/1529-0131(200008)43:8<1849::AID-ANR22>3.0.CO;2-F.

   [↑](#endnote-ref-3)
3. Giannini EH, Ilowite NT, Lovell DJ, Wallace CA, Rabinovich CE, Reiff A, Higgins G, Gottlieb B, Singer NG, Chon Y, Lin SL, Baumgartner SW; Pediatric Rheumatology Collaborative Study Group. Long-term safety and effectiveness of etanercept in children with selected categories of juvenile idiopathic arthritis. Arthritis Rheum. 2009; 60(9):2794-804. doi: 10.1002/art.24777. [↑](#endnote-ref-4)
4. Lovell DJ, Reiff A, Ilowite NT, Wallace CA, Chon Y, Lin SL, Baumgartner SW, Giannini EH; Pediatric Rheumatology Collaborative Study Group. Safety and efficacy of up to eight years of continuous etanercept therapy in patients with juvenile rheumatoid arthritis. Arthritis Rheum. 2008; 58(5):1496-504. doi: 10.1002/art.23427. [↑](#endnote-ref-5)
5. Woerner A, Uettwiller F, Melki I, Mouy R, Wouters C, Bader-Meunier B, Quartier P. Biological treatment in systemic juvenile idiopathic arthritis: achievement of inactive disease or clinical remission on a first, second or third biological agent. RMD Open. 2015; 1(1):e000036. doi: 10.1136/rmdopen-2014-000036.   [↑](#endnote-ref-6)
6. Kearsley-Fleet L, Davies R, Lunt M, Southwood TR, Hyrich KL. Factors associated with improvement in disease activity following initiation of etanercept in children and young people with Juvenile Idiopathic Arthritis: results from the British Society for Paediatric and Adolescent Rheumatology Etanercept Cohort Study. Rheumatology (Oxford). 2016; 55(5):840-7. doi: 10.1093/rheumatology/kev434. [↑](#endnote-ref-7)
7. Horneff G, Schulz AC, Klotsche J, Hospach A, Minden K, Foeldvari I, Trauzeddel R, Ganser G, Weller-Heinemann F, Haas JP. Experience with etanercept, tocilizumab and interleukin-1 inhibitors in systemic onset juvenile idiopathic arthritis patients from the BIKER registry. Arthritis Res Ther. 2017; 19(1):256. doi: 10.1186/s13075-017-1462-2. [↑](#endnote-ref-8)
8. Klein A, Klotsche J, Hügle B, Minden K, Hospach A, Weller-Heinemann F, Schwarz T, Dressler F, Trauzeddel R, Hufnagel M, Foeldvari I, Borte M, Kuemmerle-Deschner J, Brunner J, Oommen PT, Föll D, Tenbrock K, Urban A, Horneff G. Long-term surveillance of biologic therapies in systemic-onset juvenile idiopathic arthritis: data from the German BIKER registry. Rheumatology (Oxford). 2020; 59(9):2287-2298. doi: 10.1093/rheumatology/kez577.   [↑](#endnote-ref-9)
9. Armaroli G, Klein A, Ganser G, Ruehlmann MJ, Dressler F, Hospach A, Minden K, Trauzeddel R, Foeldvari I, Kuemmerle-Deschner J, Weller-Heinemann F, Urban A, Horneff G. Long-term safety and effectiveness of etanercept in JIA: an 18-year experience from the BiKeR registry. Arthritis Res Ther. 2020; 22(1):258. doi: 10.1186/s13075-020-02326-5. [↑](#endnote-ref-10)
10. Beukelman T, Lougee A, Matsouaka RA, Collier D, Rumsey DG, Schenfeld J, Stryker S, Twilt M, Kimura Y; CARRA Registry Investigators. Patterns of etanercept use in juvenile idiopathic arthritis in the Childhood Arthritis and Rheumatology Research Alliance Registry. Pediatr Rheumatol Online J. 2021; 19(1):131. doi: 10.1186/s12969-021-00625-y. [↑](#endnote-ref-11)
11. Klotsche J, Klein A, Niewerth M, Hoff P, Windschall D, Foeldvari I, Haas JP, Horneff G, Minden K. Re-treatment with etanercept is as effective as the initial first line treatment in patients with juvenile idiopathic arthritis. Arthritis Res Ther. 2021; 23(1):118. doi: 10.1186/s13075-021-02492-0. [↑](#endnote-ref-12)
12. Ruperto N, Lovell DJ, Quartier P, Paz E, Rubio-Pérez N, Silva CA, Abud-Mendoza C, Burgos-Vargas R, Gerloni V, Melo-Gomes JA, Saad-Magalhães C, Chavez-Corrales J, Huemer C, Kivitz A, Blanco FJ, Foeldvari I, Hofer M, Horneff G, Huppertz HI, Job-Deslandre C, Loy A, Minden K, Punaro M, Nunez AF, Sigal LH, Block AJ, Nys M, Martini A, Giannini EH; Paediatric Rheumatology International Trials Organization and the Pediatric Rheumatology Collaborative Study Group. Long-term safety and efficacy of abatacept in children with juvenile idiopathic arthritis. Arthritis Rheum. 2010; 62(6):1792-802. doi: 10.1002/art.27431. [↑](#endnote-ref-13)
13. Alexeeva EI, Valieva SI, Bzarova TM, Semikina EL, Isaeva KB, Lisitsyn AO, Denisova RV, Chistyakova EG. Efficacy and safety of repeat courses of rituximab treatment in patients with severe refractory juvenile idiopathic arthritis. Clin Rheumatol. 2011; 30(9):1163-72. doi: 10.1007/s10067-011-1720-7. [↑](#endnote-ref-14)
14. Quartier P, Allantaz F, Cimaz R, Pillet P, Messiaen C, Bardin C, Bossuyt X, Boutten A, Bienvenu J, Duquesne A, Richer O, Chaussabel D, Mogenet A, Banchereau J, Treluyer JM, Landais P, Pascual V. A multicentre, randomised, double-blind, placebo-controlled trial with the interleukin-1 receptor antagonist anakinra in patients with systemic-onset juvenile idiopathic arthritis (ANAJIS trial). Ann Rheum Dis. 2011; 70(5):747-54. doi: 10.1136/ard.2010.134254. [↑](#endnote-ref-15)
15. Atemnkeng Ntam V, Klein A, Horneff G. Safety and efficacy of anakinra as first-line or second-line therapy for systemic onset juvenile idiopathic arthritis - data from the German BIKER registry. Expert Opin Drug Saf. 2021; 20(1):93-100. doi: 10.1080/14740338.2021.1843631. [↑](#endnote-ref-16)
16. Zeft A, Hollister R, LaFleur B, Sampath P, Soep J, McNally B, Kunkel G, Schlesinger M, Bohnsack J. Anakinra for systemic juvenile arthritis: the Rocky Mountain experience. J Clin Rheumatol. 2009; 15(4):161-4. doi: 10.1097/RHU.0b013e3181a4f459. [↑](#endnote-ref-17)
17. Nigrovic PA, Mannion M, Prince FH, Zeft A, Rabinovich CE, van Rossum MA, Cortis E, Pardeo M, Miettunen PM, Janow G, Birmingham J, Eggebeen A, Janssen E, Shulman AI, Son MB, Hong S, Jones K, Ilowite NT, Cron RQ, Higgins GC. Anakinra as first-line disease-modifying therapy in systemic juvenile idiopathic arthritis: report of forty-six patients from an international multicenter series. Arthritis Rheum. 2011; 63(2):545-55. doi: 10.1002/art.30128. [↑](#endnote-ref-18)
18. Marvillet I, Penadés IC, Montesinos BL, et al. Anakinra treatment in patients with systemic-onset juvenil idiopathic arthritis: “The Valencia Experience”. Pediatr Rheumatol 9, P71 (2011). doi: 10.1186/1546-0096-9-S1-P71. [↑](#endnote-ref-19)
19. Vastert SJ, de Jager W, Noordman BJ, Holzinger D, Kuis W, Prakken BJ, Wulffraat NM. Effectiveness of first-line treatment with recombinant interleukin-1 receptor antagonist in steroid-naive patients with new-onset systemic juvenile idiopathic arthritis: results of a prospective cohort study. Arthritis Rheumatol. 2014; 66(4):1034-43. doi: 10.1002/art.38296. [↑](#endnote-ref-20)
20. Lainka E, Baehr M, Raszka B, Haas JP, Hügle B, Fischer N, Foell D, Hinze C, Weissbarth-Riedel E, Kallinich T, Horneff G, Windschall D, Lilienthal E, Niehues T, Neudorf U, Berendes R, Küster RM, Oommen PT, Rietschel C, Lutz T, Weller-Heinemann F, Tenbrock K, Heubner GL, Klotsche J, Wittkowski H. Experiences with IL-1 blockade in systemic juvenile idiopathic arthritis - data from the German AID-registry. Pediatr Rheumatol Online J. 2021; 19(1):38. doi: 10.1186/s12969-021-00510-8. [↑](#endnote-ref-21)
21. De Benedetti F, Brunner HI, Ruperto N, Kenwright A, Wright S, Calvo I, Cuttica R, Ravelli A, Schneider R, Woo P, Wouters C, Xavier R, Zemel L, Baildam E, Burgos-Vargas R, Dolezalova P, Garay SM, Merino R, Joos R, Grom A, Wulffraat N, Zuber Z, Zulian F, Lovell D, Martini A; PRINTO; PRCSG. Randomized trial of tocilizumab in systemic juvenile idiopathic arthritis. N Engl J Med. 2012; 367(25):2385-95. doi: 10.1056/NEJMoa1112802. [↑](#endnote-ref-22)
22. Kostik MM, Dubko MF, Masalova VV, Snegireva LS, Kornishina TL, Chikova IA, Isupova EA, Kuchinskaya EM, Glebova NI, Buchinskaya NV, Kalashnikova OV, Chasnyk VG. Successful treatment with tocilizumab every 4 weeks of a low disease activity group who achieve a drug-free remission in patients with systemic-onset juvenile idiopathic arthritis. Pediatr Rheumatol Online J. 2015; 13:4. doi: 10.1186/1546-0096-13-4. [↑](#endnote-ref-23)
23. Yokota S, Imagawa T, Mori M, Miyamae T, Aihara Y, Takei S, Iwata N, Umebayashi H, Murata T, Miyoshi M, Tomiita M, Nishimoto N, Kishimoto T. Efficacy and safety of tocilizumab in patients with systemic-onset juvenile idiopathic arthritis: a randomised, double-blind, placebo-controlled, withdrawal phase III trial. Lancet; 371(9617):998-1006. doi: 10.1016/S0140-6736(08)60454-7. [↑](#endnote-ref-24)
24. Yokota S, Imagawa T, Mori M, Miyamae T, Takei S, Iwata N, Umebayashi H, Murata T, Miyoshi M, Tomiita M, Nishimoto N, Kishimoto T. Long-term treatment of systemic juvenile idiopathic arthritis with tocilizumab: results of an open-label extension study in Japan. Ann Rheum Dis. 2013; 72(4):627-8. doi: 10.1136/annrheumdis-2012-202310. [↑](#endnote-ref-25)
25. Mihailova D, Varbanova B, Stefanov S, et al. PReS-FINAL-2038: Clinical efficacy and tolerability of tocilizumab in patients with persistant systemic JIA. Pediatr Rheumatol 11, P51 (2013). doi: 10.1186/1546-0096-11-S2-P51. [↑](#endnote-ref-26)
26. Kubota T. Changes In Serum IL-18 Level In Systemic Juvenile Idiopathic Arthritis Patients Who Attained Drug-Free Remission By Tocilizumab. [↑](#endnote-ref-27)
27. [De Benedetti](https://www.ncbi.nlm.nih.gov/pubmed/?term=De%20Benedetti%20F%5BAuthor%5D) F, [Ruperto](https://www.ncbi.nlm.nih.gov/pubmed/?term=Ruperto%20N%5BAuthor%5D) N, [Brunner](https://www.ncbi.nlm.nih.gov/pubmed/?term=Brunner%20H%5BAuthor%5D) H, [A Grom](https://www.ncbi.nlm.nih.gov/pubmed/?term=Grom%20A%5BAuthor%5D), [N Wulffraat](https://www.ncbi.nlm.nih.gov/pubmed/?term=Wulffraat%20N%5BAuthor%5D), [M Henrickson](https://www.ncbi.nlm.nih.gov/pubmed/?term=Henrickson%20M%5BAuthor%5D), [R Jerath](https://www.ncbi.nlm.nih.gov/pubmed/?term=Jerath%20R%5BAuthor%5D), [Y Kimura](https://www.ncbi.nlm.nih.gov/pubmed/?term=Kimura%20Y%5BAuthor%5D), [AK Kadva](https://www.ncbi.nlm.nih.gov/pubmed/?term=Kadva%20A%5BAuthor%5D), [J Wang](https://www.ncbi.nlm.nih.gov/pubmed/?term=Wang%20J%5BAuthor%5D), [A Martini](https://www.ncbi.nlm.nih.gov/pubmed/?term=Martini%20A%5BAuthor%5D), [D Lovell](https://www.ncbi.nlm.nih.gov/pubmed/?term=Lovell%20D%5BAuthor%5D). Tapering and withdrawal of tocilizumab in patients with systemic juvenile idiopathic arthritis in inactive disease: results from an alternative dosing regimen in the TENDER study. Pediatr Rheumatol Online J. 2014; 12(Suppl 1): O13. doi: 10.1186/1546-0096-12-S1-O13.  [↑](#endnote-ref-28)
28. Malattia C.Assessment of Radiographic Progression in Patients With Systemic Juvenile Idiopathic Arthritis Treated With Tocilizumab: 2-Year Results From the TENDER Trial. March 2014.66 (S11).  [↑](#endnote-ref-29)
29. Yokota S, Imagawa T, Mori M, Miyamae T, Takei S, Iwata N, Umebayashi H, Murata T, Miyoshi M, Tomiita M, Nishimoto N, Kishimoto T. Long term safety and effectiveness of the anti-interleukin 6 receptor monoclonal antibody tocilizumab in patients with systemic juvenile idiopathic arthritis in Japan. J Rheumatol. 2014; 41(4):759-67. doi:10.3899/jrheum.130690. [↑](#endnote-ref-30)
30. Horneff G, Huppertz I, Haas P, et al. Safety and efficacy of tocilizumab in children with systemic juvenile idiopathic arthritis. Pediatr Rheumatol Online J. 2015;13(Suppl 1):P165. Published 2015 Sep 28. doi:10.1186/1546-0096-13-S1-P165. [↑](#endnote-ref-31)
31. Yokota S, Itoh Y, Morio T, Origasa H, Sumitomo N, Tomobe M, Tanaka K, Minota S. Tocilizumab in systemic juvenile idiopathic arthritis in a real-world clinical setting: results from 1 year of postmarketing surveillance follow-up of 417 patients in Japan. Ann Rheum Dis. 2016; 75(9):1654-60. doi: 10.1136/annrheumdis-2015-207818. [↑](#endnote-ref-32)
32. Pacharapakornpong T, Vallibhakara SA, Lerkvaleekul B, Vilaiyuk S. Comparisons of the outcomes between early and late tocilizumab treatment in systemic juvenile idiopathic arthritis. Rheumatol Int. 2017; 37(2):251-255. doi: 10.1007/s00296-016-3595-z. [↑](#endnote-ref-33)
33. Glazyrina G, Kolyadina NAB0952 Experience of tocilizumab use in treatment of juvenile idiopathic arthritis in chelyabinsk regional children hospital. Annals of the Rheumatic Diseases 2017;76:1388.  [↑](#endnote-ref-34)
34. Roszkiewicz J, Orczyk K, Smolewska E. Tocilizumab in the treatment of systemic-onset juvenile idiopathic arthritis - single-centre experience. Reumatologia. 2018; 56(5):279-284. doi: 10.5114/reum.2018.79497. [↑](#endnote-ref-35)
35. Demir S, Sönmez HE, Arslanoğlu-Aydın E, Özen S, Bilginer Y. Tocilizumab treatment in juvenile idiopathic arthritis patients: A single center experience. Turk J Pediatr. 2019; 61(2):180-185. doi: 10.24953/turkjped.2019.02.005. [↑](#endnote-ref-36)
36. Ruperto N, Chen C, Martini A, et al. FRI0572 DISABILITY AND HEALTH-RELATED QUALITY OF LIFE OUTCOMES IN PATIENTS WITH SYSTEMIC JUVENILE IDIOPATHIC ARTHRITIS TREATED WITH TOCILIZUMAB IN A PHASE 3 RANDOMIZED CONTROLLED TRIAL. Annals of the Rheumatic Diseases 2019;78:981-982. [↑](#endnote-ref-37)
37. Mallalieu NL, Wimalasundera S, Hsu JC, Douglass W, Wells C, Penades IC, Cuttica R, Huppertz HI, Joos R, Kimura Y, Milojevic D, Rosenkranz M, Schikler K, Constantin T, Wouters C. Intravenous dosing of tocilizumab in patients younger than two years of age with systemic juvenile idiopathic arthritis: results from an open-label phase 1 clinical trial. Pediatr Rheumatol Online J. 2019; 17(1):57. doi: 10.1186/s12969-019-0364-z. [↑](#endnote-ref-38)
38. Pardeo M, Wang J, Ruperto N, Alexeeva E, Chasnyk V, Schneider R, Horneff G, Huppertz HI, Minden K, Onel K, Zemel L, Martin A, Kone-Paut I, Siamopoulou-Mavridou A, Silva CA, Porter-Brown B, Bharucha KN, Brunner HI, De Benedetti F; Paediatric Rheumatology International Trials Organization (PRINTO) and the Pediatric Rheumatology Collaborative Study Group (PRCSG). Neutropenia During Tocilizumab Treatment Is Not Associated with Infection Risk in Systemic or Polyarticular-course Juvenile Idiopathic Arthritis. J Rheumatol. 2019; 46(9):1117-1126. doi: 10.3899/jrheum.180795. [↑](#endnote-ref-39)
39. Ruperto N, Brunner HI, Ramanan AV, Horneff G, Cuttica R, Henrickson M, Anton J, Boteanu AL, Penades IC, Minden K, Schmeling H, Hufnagel M, Weiss JE, Pardeo M, Nanda K, Roth J, Rubio-Pérez N, Hsu JC, Wimalasundera S, Wells C, Bharucha K, Douglass W, Bao M, Mallalieu NL, Martini A, Lovell D, Benedetti F; Paediatric Rheumatology INternational Trials Organisation (PRINTO) and the Paediatric Rheumatology Collaborative Study Group (PRCSG). Subcutaneous dosing regimens of tocilizumab in children with systemic or polyarticular juvenile idiopathic arthritis. Rheumatology (Oxford). 2021; 60(10):4568-4580. doi: 10.1093/rheumatology/keab047. [↑](#endnote-ref-40)
40. Yan X, Tang W, Zhang Z, Zhang Y, Luo C, Tang X. Tocilizumab in Systemic Juvenile Idiopathic Arthritis: Response Differs by Disease Duration at Medication Initiation and by Phenotype of Disease. Front Pediatr. 2021; 9:735846. doi: 10.3389/fped.2021.735846. [↑](#endnote-ref-41)
41. Ruperto N, Brunner HI, Quartier P, Constantin T, Wulffraat N, Horneff G, Brik R, McCann L, Kasapcopur O, Rutkowska-Sak L, Schneider R, Berkun Y, Calvo I, Erguven M, Goffin L, Hofer M, Kallinich T, Oliveira SK, Uziel Y, Viola S, Nistala K, Wouters C, Cimaz R, Ferrandiz MA, Flato B, Gamir ML, Kone-Paut I, Grom A, Magnusson B, Ozen S, Sztajnbok F, Lheritier K, Abrams K, Kim D, Martini A, Lovell DJ; PRINTO; PRCSG. Two randomized trials of canakinumab in systemic juvenile idiopathic arthritis. N Engl J Med. 2012; 367(25):2396-406. doi: 10.1056/NEJMoa1205099. [↑](#endnote-ref-42)
42. Ruperto N, Brunner HI, Quartier P, Constantin T, Wulffraat NM, Horneff G, Kasapcopur O, Schneider R, Anton J, Barash J, Berner R, Corona F, Cuttica R, Fouillet-Desjonqueres M, Fischbach M, Foster HE, Foell D, Radominski SC, Ramanan AV, Trauzeddel R, Unsal E, Levy J, Vritzali E, Martini A, Lovell DJ; Paediatric Rheumatology International Trials Organisation (PRINTO) and the Pediatric Rheumatology Collaborative Study Group (PRCSG). Canakinumab in patients with systemic juvenile idiopathic arthritis and active systemic features: results from the 5-year long-term extension of the phase III pivotal trials. Ann Rheum Dis. 2018; 77(12):1710-1719. doi: 10.1136/annrheumdis-2018-213150. [↑](#endnote-ref-43)
43. Alexeeva E, Dvoryakovskaya T, Isaeva K, et al. FRI0553 CANAKINUMAB AS A FIRST-LINE AND SECOND-LINE BIOLOGIC FOR TREATMENT OF SYSTEMIC JUVENILE IDIOPATHIC ARTHRITIS IN CHILDREN UNDER 4 YEARS OF AGE. Annals of the Rheumatic Diseases 2019;78:972. [↑](#endnote-ref-44)
44. Quartier P, Alexeeva E, Constantin T, Chasnyk V, Wulffraat N, Palmblad K, Wouters C, I Brunner H, Marzan K, Schneider R, Horneff G, Martini A, Anton J, Wei X, Slade A, Ruperto N, Abrams K; Paediatric Rheumatology International Trials Organisation and the Pediatric Rheumatology Collaborative Study Group. Tapering Canakinumab Monotherapy in Patients With Systemic Juvenile Idiopathic Arthritis in Clinical Remission: Results From a Phase IIIb/IV Open-Label, Randomized Study. Arthritis Rheumatol. 2021; 73(2):336-346. doi: 10.1002/art.41488. [↑](#endnote-ref-45)
45. Horneff G, Ruperto N, Brunner H, et al. Long term efficacy and safety of canakinumab in children with systemic juvenile idiopathic arthritis with and without fever. Pediatr Rheumatol 13, O83 (2015). doi: 10.1186/1546-0096-13-S1-O83. [↑](#endnote-ref-46)
46. Ruperto N, Quartier P, Wulffraat N, et al. A phase II, multicenter, open-label study evaluating dosing and preliminary safety and efficacy of canakinumab in systemic juvenile idiopathic arthritis with active systemic features. Arthritis Rheum. 2012;64 (2):557–567. [↑](#endnote-ref-47)
47. Feist E, Quartier P, Fautrel B, Schneider R, Sfriso P, Efthimiou P, Cantarini L, Lheritier K, Leon K, Karyekar CS, Speziale A. Efficacy and safety of canakinumab in patients with Still's disease: exposure-response analysis of pooled systemic juvenile idiopathic arthritis data by age groups. Clin Exp Rheumatol. 2018; 36(4):668-675. [↑](#endnote-ref-48)
48. Brunner HI, Quartier P, Alexeeva E, Constantin T, Koné-Paut I, Marzan K, Schneider R, Wulffraat NM, Chasnyk V, Tirosh I, Kallinich T, Kuemmerle-Deschner J, Wouters C, Lauwerys B, Nikishina I, Trachana M, Vougiouka O, Martini A, Lovell DJ, Levy J, Vritzali E, Ruperto N; Paediatric Rheumatology International Trials Organisation, the Pediatric Rheumatology Collaborative Study Group. Efficacy and Safety of Canakinumab in Patients With Systemic Juvenile Idiopathic Arthritis With and Without Fever at Baseline: Results From an Open-Label, Active-Treatment Extension Study. Arthritis Rheumatol. 2020; 72(12):2147-2158. doi: 10.1002/art.41436. [↑](#endnote-ref-49)
49. Nishimura K, Hara R, Umebayashi H, Takei S, Iwata N, Imagawa T, Shimizu M, Tomiita M, Seko N, Kitawaki T, Yokota S. Efficacy and safety of canakinumab in systemic juvenile idiopathic arthritis: 48-week results from an open-label phase III study in Japanese patients. Mod Rheumatol. 2021; 31(1):226-234. doi: 10.1080/14397595.2020.1783163. [↑](#endnote-ref-50)
50. Coto C, Varela G, Hernández V, del Rosario M, López-Saura P. Use of recombinant interferon gamma in pediatric patients with advanced juvenile chronic arthritis. Biotherapy. 1998; 11(1):15-20. doi: 10.1023/a:1007932130188. [↑](#endnote-ref-51)
51. Lovell DJ, Giannini EH, Reiff AO, Kimura Y, Li S, Hashkes PJ, Wallace CA, Onel KB, Foell D, Wu R, Biedermann S, Hamilton JD, Radin AR. Long-term safety and efficacy of rilonacept in patients with systemic juvenile idiopathic arthritis. Arthritis Rheum. 2013; 65(9):2486-96. doi: 10.1002/art.38042. [↑](#endnote-ref-52)
52. Ilowite NT, Prather K, Lokhnygina Y, Schanberg LE, Elder M, Milojevic D, Verbsky JW, Spalding SJ, Kimura Y, Imundo LF, Punaro MG, Sherry DD, Tarvin SE, Zemel LS, Birmingham JD, Gottlieb BS, Miller ML, O'Neil K, Ruth NM, Wallace CA, Singer NG, Sandborg CI. Randomized, double-blind, placebo-controlled trial of the efficacy and safety of rilonacept in the treatment of systemic juvenile idiopathic arthritis. Arthritis Rheumatol. 2014; 66(9):2570-9. doi: 10.1002/art.38699. [↑](#endnote-ref-53)
53. Gerloni V, Cimaz R, Gattinara M, Arnoldi C, Pontikaki I, Fantini F. Efficacy and safety profile of cyclosporin A in the treatment of juvenile chronic (idiopathic) arthritis. Results of a 10-year prospective study. Rheumatology (Oxford). 2001; 40(8):907-13. doi: 10.1093/rheumatology/40.8.907. [↑](#endnote-ref-54)
54. Pal P, Giri PP, Sinha R. Cyclosporine in Resistant Systemic Arthritis - A Cheaper Alternative to Biologics. Indian J Pediatr. 2019; 86(7):590-594. doi: 10.1007/s12098-019-02912-9. [↑](#endnote-ref-55)
55. Brinkman DM, de Kleer IM, ten Cate R, van Rossum MA, Bekkering WP, Fasth A, van Tol MJ, Kuis W, Wulffraat NM, Vossen JM. Autologous stem cell transplantation in children with severe progressive systemic or polyarticular juvenile idiopathic arthritis: long-term follow-up of a prospective clinical trial. Arthritis Rheum. 2007; 56(7):2410-21. doi: 10.1002/art.22656. [↑](#endnote-ref-56)
56. Silva J, Ladomenou F, Carpenter B, Chandra S, Sedlacek P, Formankova R, Grandage V, Friswell M, Cant AJ, Nademi Z, Slatter MA, Gennery AR, Hambleton S, Flood TJ, Lucchini G, Chiesa R, Rao K, Amrolia PJ, Brogan P, Wedderburn LR, Glanville JM, Hough R, Marsh R, Abinun M, Veys P. Allogeneic hematopoietic stem cell transplantation for severe, refractory juvenile idiopathic arthritis. Blood Adv. 2018; 2(7):777-786. doi: 10.1182/bloodadvances.2017014449. [↑](#endnote-ref-57)
57. Swart JF, de Roock S, Nievelstein RAJ, Slaper-Cortenbach ICM, Boelens JJ, Wulffraat NM. Bone-marrow derived mesenchymal stromal cells infusion in therapy refractory juvenile idiopathic arthritis patients. Rheumatology (Oxford). 2019; 58(10):1812-1817. doi: 10.1093/rheumatology/kez157. [↑](#endnote-ref-58)
58. Bruck N, Suttorp M, Kabus M, Heubner G, Gahr M, Pessler F. Rapid and sustained remission of systemic juvenile idiopathic arthritis-associated macrophage activation syndrome through treatment with anakinra and corticosteroids. J Clin Rheumatol. 2011; 17(1):23-7. doi: 10.1097/RHU.0b013e318205092d. [↑](#endnote-ref-59)
59. Phadke O, Rouster-Stevens K, Giannopoulos H, Chandrakasan S, Prahalad S. Intravenous administration of anakinra in children with macrophage activation syndrome. Pediatr Rheumatol Online J. 2021; 19(1):98. doi: 10.1186/s12969-021-00585-3. [↑](#endnote-ref-60)
60. Sönmez HE, Demir S, Bilginer Y, Özen S. Anakinra treatment in macrophage activation syndrome: a single center experience and systemic review of literature. Clin Rheumatol. 2018; 37(12):3329-3335. doi: 10.1007/s10067-018-4095-1. [↑](#endnote-ref-61)
61. Kounami S, Yoshiyama M, Nakayama K, Okuda M, Okuda S, Aoyagi N, Yoshikawa N. Macrophage activation syndrome in children with systemic-onset juvenile chronic arthritis. Acta Haematol. 2005; 113(2):124-9. doi: 10.1159/000083450. [↑](#endnote-ref-62)
62. Bennett TD, Fluchel M, Hersh AO, Hayward KN, Hersh AL, Brogan TV, Srivastava R, Stone BL, Korgenski EK, Mundorff MB, Casper TC, Bratton SL. Macrophage activation syndrome in children with systemic lupus erythematosus and children with juvenile idiopathic arthritis. Arthritis Rheum. 2012; 64(12):4135-42. doi: 10.1002/art.34661. [↑](#endnote-ref-63)
63. Lin CI, Yu HH, Lee JH, Wang LC, Lin YT, Yang YH, Chiang BL. Clinical analysis of macrophage activation syndrome in pediatric patients with autoimmune diseases. Clin Rheumatol. 2012; 31(8):1223-30. doi: 10.1007/s10067-012-1998-0. [↑](#endnote-ref-64)
64. Minoia F, Davì S, Horne A, Demirkaya E, Bovis F, Li C, Lehmberg K, Weitzman S, Insalaco A, Wouters C, Shenoi S, Espada G, Ozen S, Anton J, Khubchandani R, Russo R, Pal P, Kasapcopur O, Miettunen P, Maritsi D, Merino R, Shakoory B, Alessio M, Chasnyk V, Sanner H, Gao YJ, Huasong Z, Kitoh T, Avcin T, Fischbach M, Frosch M, Grom A, Huber A, Jelusic M, Sawhney S, Uziel Y, Ruperto N, Martini A, Cron RQ, Ravelli A; Pediatric Rheumatology International Trials Organization; Childhood Arthritis and Rheumatology Research Alliance; Pediatric Rheumatology Collaborative Study Group; Histiocyte Society. Clinical features, treatment, and outcome of macrophage activation syndrome complicating systemic juvenile idiopathic arthritis: a multinational, multicenter study of 362 patients. Arthritis Rheumatol. 2014; 66(11):3160-9. doi: 10.1002/art.38802. [↑](#endnote-ref-65)
65. Boteanu, A.L., Cañamero, M.A.B., Romero, V.M. et al. Macrophage activation syndrome during treatment with biological therapy in patients with systemic juvenile idiopathic arthritis. Pediatr Rheumatol 12, P214 (2014). <https://doi.org/10.1186/1546-0096-12-S1-P214>. [↑](#endnote-ref-66)
66. Aytaç S, Batu ED, Ünal Ş, Bilginer Y, Çetin M, Tuncer M, Gümrük F, Özen S. Macrophage activation syndrome in children with systemic juvenile idiopathic arthritis and systemic lupus erythematosus. Rheumatol Int. 2016; 36(10):1421-9. doi: 10.1007/s00296-016-3545-9. [↑](#endnote-ref-67)
67. Kounami S, Yoshiyama M, Nakayama K, Okuda M, Okuda S, Aoyagi N, Yoshikawa N. Macrophage activation syndrome in children with systemic-onset juvenile chronic arthritis. Acta Haematol. 2005; 113(2):124-9. doi: 10.1159/000083450. [↑](#endnote-ref-68)
68. Palmblad K, Schierbeck H, Sundberg E, Horne AC, Erlandsson Harris H, Henter JI, Andersson U. Therapeutic administration of etoposide coincides with reduced systemic HMGB1 levels in macrophage activation syndrome. Mol Med. 2021; 27(1):48. doi: 10.1186/s10020. [↑](#endnote-ref-69)
69. Lin CI, Yu HH, Lee JH, Wang LC, Lin YT, Yang YH, Chiang BL. Clinical analysis of macrophage activation syndrome in pediatric patients with autoimmune diseases. Clin Rheumatol. 2012; 31(8):1223-30. doi: 10.1007/s10067-012-1998-0. [↑](#endnote-ref-70)
70. Singh S, Chandrakasan S, Ahluwalia J, Suri D, Rawat A, Ahmed N, Das R, Sachdeva MU, Varma N. Macrophage activation syndrome in children with systemic onset juvenile idiopathic arthritis: clinical experience from northwest India. Rheumatol Int. 2012; 32(4):881-6. doi: 10.1007/s00296-010-1711-z. [↑](#endnote-ref-71)
71. Grom AA, Ilowite NT, Pascual V, Brunner HI, Martini A, Lovell D, Ruperto N; Paediatric Rheumatology International Trials Organisation and the Pediatric Rheumatology Collaborative Study Group, Leon K, Lheritier K, Abrams K. Rate and Clinical Presentation of Macrophage Activation Syndrome in Patients With Systemic Juvenile Idiopathic Arthritis Treated With Canakinumab. Arthritis Rheumatol. 2016; 68(1):218-28. doi: 10.1002/art.39407. [↑](#endnote-ref-72)
72. Nakagishi Y, Shimizu M, Kasai K, Miyoshi M, Yachie A. Successful therapy of macrophage activation syndrome with dexamethasone palmitate. Mod Rheumatol. 2016; 26(4):617-20. doi: 10.3109/14397595.2014.906053. [↑](#endnote-ref-73)
73. Rodionovskaya S, Nikishina.IAB0700 Successful experience with tocilizumab in two patients with macrophage activation syndrome associated with juvenile idiopathic arthritisAnnals of the Rheumatic Diseases 2013;7 2: A1001-A1002.  [↑](#endnote-ref-74)
