# Supplementary material for: Pan American League of Associations for Rheumatology treatment recommendations for systemic juvenile idiopathic arthritis
Source: Rheumatol Adv Pract. 2025 Nov 11;9(4):rkaf087. doi: 10.1093/rap/rkaf087 (PMC12607261; doi:10.1093/rap/rkaf087)
Supplement: rkaf087_Supplementary_Data [file rkaf087_supplementary_data.zip › Supplementary_Data_5._Systemic_JIA_Educative_Appendix.docx]

**Table 1: Drugs**

| **DRUG** | **Doses** |
| --- | --- |
| Methylprednisolone | High Dose IV steroid (pulse therapy )(20–30 mg/kg/day [max. 1000 mg/day) for 3-5 days. Maximum doses for glucocorticoids IV |
| Prednisone | Equivalent 1–2 mg/kg/day (max. 60 mg/day). |
| Methotrexate | 10-15 mg / m2 week (max.25 mg/week) |
| Anakinra | 1-2 mg/kg/day or (max. 100 mg/day) |
| Canakinumab | 4 mg/kg (max. 300 mg q4 weeks) |
| Rilonacept | 4.4 mg/kg (loading dose) SC  2.2 mg/kg weekly SC |
| Tocilizumab | ≥2 years (<30 kg): 12 mg/kg IV q2weeks  ≥2 years (≥30 kg): 8 mg/kg IV q2weeks (max. 800 mg dose)  Infusion over 60 minutes  SC injection  ≥2 years (<30 kg): 162 mg SC once q2weeks  ≥2 years (≥30 kg): 162 mg SC once weekly |
| Cyclosporine A | 3-5mg/kg/day/PO/twice a day. |
| Tofacitinib * | 5-7 kg (2 mg o 2 ml) twice a day  7-10 kg (2,5 mg o 2 ml) twice a day  10-15 kg (3 mg o 3 ml) twice a day  15-25 kg (3,5 mg o 3,5 ml) twice a day  25-40 kg (4 mg o 4 ml) twice a day  40 kg (5 mg o 5 ml) twice a day |
| Adalimumab | ≥2 years (10- <30 kg): 20 mg SC once q2weeks  ≥2 years (≥30 kg):40 mg SC once q2weeks |
| Etanercept | ≥2 years: 0.8 mg/kg SC week (max 50 mg/week) |

* New agents are being developed like Janus Kinase (JAK) inhibitors. However, at the time of writing these recommendations, there were no controlled studies or systematic reviews on these drugs

**References**

1- Beukelman T, Tomlinson G, Nigrovic PA, Dennos A, Del Gaizo V, Jelinek M, et al. First-line options for systemic juvenile idiopathic arthritis treatment: an observational study of Childhood Arthritis and Rheumatology Research Alliance Consensus Treatment Plans. Pediatr Rheumatol Online J [Internet]. 2022;20(1):113. doi: [10.1186/s12969-022-00768-6](http://dx.doi.org/10.1186/s12969-022-00768-6)

2- Asociación Española de Pediatría. Pediamécum. Madrid: Asociación Española de Pediatría. Disponible en: <https://www.pediamecum.es>

3- Verweyen EL, Schulert GS. Interfering with interferons: targeting the JAK-STAT pathway in complications of systemic juvenile idiopathic arthritis (SJIA). Rheumatology (Oxford) [Internet]. 2022;62(5):926–35. doi: 10.1093/rheumatology/kead004
